# Human challenge study with a Shigella bioconjugate vaccine: Analyses of clinical efficacy and correlate of protection

**DOI:** 10.1016/j.ebiom.2021.103310

**Published:** 2021-04-13

**Authors:** Kawsar R. Talaat, Cristina Alaimo, Patricia Martin, A. Louis Bourgeois, Anita M. Dreyer, Robert W. Kaminski, Chad K. Porter, Subhra Chakraborty, Kristen A. Clarkson, Jessica Brubaker, Daniel Elwood, Rahel Frölich, Barbara DeNearing, Hailey Weerts, Brittany L. Feijoo, Jane Halpern, David Sack, Mark S. Riddle, Veronica Gambillara Fonck

**Affiliations:** a Department of International Health, Johns Hopkins Bloomberg School of Public Health, Baltimore, MD, United States; b LimmaTech Biologics AG, Schlieren, Switzerland; c Walter Reed Army Institute of Research, Silver Spring, MD, United States; d Naval Medical Research Center, Silver Spring, MD, United States; e Now at PATH Center for Vaccine Innovation and Access, Washington, DC, United States

**Keywords:** Shigella, Shigella flexneri 2a, Vaccine, Bioconjugate vaccine, Controlled human challenge study

## Abstract

**Background:**

Shigellosis is a major cause of moderate to severe diarrhoea and dysentery in children under 5 years of age in low and middle-income countries. The Flexyn2a vaccine conjugates the *O*-polysaccharide of *Shigella flexneri* 2a to *Pseudomonas aeruginosa* exotoxin *A*. We describe a Phase 2b proof-of-concept challenge study that evaluated safety, immunogenicity, and efficacy of the Flexyn2a vaccine to protect against shigellosis.

**Methods:**

In this randomized, double blind, placebo-controlled trial, healthy adults were randomized 1:1 to receive Flexyn2a (10 µg) or placebo intramuscularly, twice, 4 weeks apart, followed by challenge 4 weeks later with 1500 colony forming units (CFUs) of *S. flexneri* 2a strain 2457T. The primary outcome was vaccine-induced protection. *S. flexneri* 2a lipopolysaccharide (LPS)-specific immune responses were assessed.

**Findings:**

Sixty-seven subjects were enrolled, 34 received vaccine and 33 placebo. The vaccine was well tolerated; the majority of adverse events were mild in nature. Thirty vaccinees and 29 placebo recipients received the *S. flexneri* 2a challenge. Vaccination resulted in a 30.2% reduction in shigellosis compared with placebo (13/30 vs. 18/29; *p* = 0.11; 95% CI -15 to 62.6). Vaccine efficacy was more robust against severe disease, reaching 51.7% (*p* = 0.015, 95% CI 5.3 to 77.9) against moderate/severe diarrhoea or dysentery concurrent with fever or severe enteric symptoms and 72.4% (*p* = 0.07) against more severe diarrhoea (≥10 lose stools or ≥1000 g loose stools/24 h). Vaccinated subjects were less likely to need early antibiotic intervention following challenge (protective efficacy 51.7%, *p* = 0.01; 95% CI 9 to 76.8). In those who developed shigellosis, vaccinated subjects had a lower disease severity score (*p* = 0.002) than placebo-recipients. Additionally, LPS-specific serum IgG responses in Flexyn2a recipients were associated with protection against disease (*p* = 0.0016) and with a decreased shigellosis disease score (*p* = 0.002).

**Interpretation:**

The Flexyn2a bioconjugate vaccine was immunogenic, well tolerated and protected against severe illness after *Shigella* challenge and is a promising *Shigella* vaccine construct. We identified a strong association between anti-*S. flexneri* 2a serum IgG and a reduction in disease outcomes. (Clinicaltrials.gov, NCT02646371.)

**Funding:**

Funding for this study was through a grant from the Wellcome Trust.

**Research in Context**

**Evidence before this study**

There is currently no licensed *Shigella* vaccine. A PubMed search for “shigella” AND “vaccine” unlimited by language, restricted to clinical trials revealed 70 articles about several vaccine constructs, including the bioconjugate vaccine described in this manuscript. No other bioconjugate vaccines are in clinical trials, although there are several other *Shigella* vaccines, including a chemical conjugate vaccine in development. An earlier generation of conjugate vaccine, developed by John Robbins and colleagues was found to be safe, immunogenic and effective in Israeli military adults. When tested in children 1–4 years old, the *S. flexneri* 2a and *S. sonnei* conjugates were safe, and while there were not enough cases of *S. flexneri* 2a to assess efficacy against this pathogen, the *S. sonnei* vaccine did not protect the youngest children against *S. sonnei*. A phase 1 trial with the Shigella bioconjugate Flexyn2a has been published, describing the initial safety and immunogenicity of this vaccine. This Phase 2 study was designed to assess the efficacy of the Flexyn2a vaccine against challenge with *S. flexneri* 2a in a controlled human infection study.

**Added value for this study**

This is the first study investigating the efficacy of the Flexyn2a *Shigella* bioconjugate vaccine in adults. This study utilized the controlled human challenge model to demonstrate the efficacy of the Flexyn2a vaccine against clinical shigellosis, and the vaccine was found to be particularly effective against severe shigellosis. This study also confirmed the safety and immunogenicity observed in the earlier Phase 1 trial. It is also the first study to evaluate efficacy utilizing the recently published consensus endpoints for *Shigella* controlled human challenge studies.

**Implication of all the available evidence**

This study shows that the Flexyn2a vaccine is well tolerated, immunogenic, and is protective against severe shigellosis, although protection against the per-protocol definition of shigellosis did not reach statistical significance. The results identify a potential correlate of immunity for *Shigella*, the LPS-specific serum IgG response and also suggest that a Shigella vaccine impacting on the incidence and severity of Shigellosis can potentially reduce the need for antibiotic treatment. This study is an important step forward toward the eventual licensure of a vaccine against *Shigella*.

Alt-text: Unlabelled box

## 1 Introduction

*Shigella* spp. cause moderate to severe diarrhoea and dysentery predominantly in children under 5 years of age in low to middle income countries [1]. Shigellosis is often characterized by systemic and enteric symptoms and can be life threatening in vulnerable hosts [1], [2], [3]. In addition to the mortality, morbidity and long-term consequences associated with shigellosis (i.e. stunting and wasting) [4], [5], [6], [7], reports of spreading resistance to antibiotics highlight the need for primary prevention [8].

Despite ongoing Shigella vaccine development efforts for almost 100 years [9,10], no licensed vaccine is available. Previously, chemical conjugates were shown to be effective in adults but not in children under 3 years of age [11,12] and protection was associated with the LPS-specific serum IgG response post-vaccination. These early products were never commercially developed. A vaccine that is simple to manufacture and more effective in children is greatly needed, and conjugate vaccines produced with bioconjugation technology have shown great potential [13], [14], [15], [16]. Flexyn2a is a bioconjugate vaccine composed of the *O*-polysaccharide of *Shigella flexneri* 2a enzymatically linked to the exotoxin A of *Pseudomonas aeruginosa* (EPA) using a reproducible and greatly simplified conjugation process [17]. In a recent Phase 1 study, Flexyn2a exhibited a good safety and robust immunogenicity profile [15]. We describe here a proof-of-concept efficacy study performed to demonstrate the ability of Flexyn2a to protect against shigellosis following challenge with *S. flexneri* 2a strain 2457T and to determine if LPS-specific serum IgG correlates with a reduction in disease outcomes. This vaccination strategy has been broadened to create a multivalent *Shigella* vaccine targeting the most relevant circulating strains of *Shigella* in low- and middle-income countries and a phase 1/2 clinical trial in east Africa is currently ongoing (NCT04056117).

## 2 Methods

### 2.1 Clinical trial design

The trial was randomized, double-blinded and placebo-controlled. Two cohorts of 36 healthy adult volunteers were planned to be successively enrolled. In each cohort, a vaccination phase was followed by a challenge phase, with up to 30 volunteers from each cohort to be challenged.

### 2.2 Study oversight

The study was conducted at the Johns Hopkins Bloomberg School of Public Health Center for Immunization Research (CIR). The challenge phase was conducted at the CIR inpatient unit at the Johns Hopkins Bayview Medical Campus. All subjects provided written informed consent. The trial was approved by the Western Institutional Review Board in compliance with all federal regulations governing the protection of human volunteers. LimmaTech served as the sponsor of the study and developed the study design with the investigators. The investigators were responsible for study conduct and management. (Clinicaltrials.gov registration NCT02646371).

### 2.3 Vaccine

The Flexyn2a vaccine is produced *in vivo* in *E. coli* and subsequently purified and formulated as previously described [15,17]. The product has been characterized extensively including assays for content, purity, and structure [17]. Each dose contains 10 μg of *Shigella flexneri* 2a *O*-polysaccharide and approximately 50 μg EPA, the dose found to be well tolerated and immunogenic in the Phase 1 study [15]. The vaccine (or saline placebo) was administered twice, one month apart, via intramuscular injection with a dose volume of 0.5 mL. As the human challenge model is a clinical proof of concept to allow for an indication of protection of a candidate vaccine and the potential correlation with immunological parameters, no dose and schedule finding was performed. Future studies will evaluate these parameters in the target populations.

### 2.4 Challenge strain

The 2457T *Shigella flexneri* 2a challenge strain is a well-characterized *Shigella* strain manufactured under current Good Manufacturing Practice conditions at the Walter Reed Army Institute of Research Pilot BioProduction Facility in Silver Spring, Maryland [18]. The target challenge-dose of 1500 CFU was chosen based on published studies [18,19] as one that could be safely utilized and would yield a sufficiently high shigellosis attack rate (AR). The pre-determined acceptable range for the challenge inoculum was 1500–2000 CFU.

### 2.5 Study population and enrolment criteria

A consensus description of the methods involved in the conduct of a *Shigella* challenge has recently been published [20]. Essentially, volunteers were healthy male and nonpregnant female adults between 18 and 50 years of age recruited from the Mid-Atlantic area. Informed consent was a rigorous and iterative process to ensure comprehension of the trial and their participation. To ensure eligibility criteria were met, medical history, laboratory tests and a complete physical exam were performed. among other requirements, volunteers were eligible if they had no significant medical history or exam findings of inflammatory arthritis, chronic gastrointestinal problems or irritable bowel syndrome, were HLA-B27 negative (to decrease the risk of reactive arthritis after challenge) and had no recent history of traveller's diarrhoea or participation in other *Shigella* trials within 3 years. In addition, eligibility was limited to volunteers with a *S. flexneri* 2a-LPS specific serum IgG ELISA endpoint below 2500^15^ in an attempt to recruit a *Shigella*-naïve population and more closely align with the immunologically naïve infant and young child population that is the main target group for a Shigella vaccine.

### 2.6 Randomization and masking

This study was a randomized, double blind, and placebo-controlled trial. For each cohort, randomization was done in six blocks (randomly ordered) of six subjects each, via the interactive web response system (IWRS) AdvantageEDC by the CRO, the EMMES Corporation, and the treatment key was provided to the pharmacy which dispensed the vaccine. The volunteers, site personnel, laboratory staff and monitors were blinded, as were some representatives of the sponsor. The Investigational pharmacist, other personnel at the CRO and at the sponsor were unblinded but had no access to the volunteers or study data except for the pharmacy and laboratory records. The blinding of the subjects was maintained throughout the entire study.

### 2.7 Safety monitoring and study procedures

Many of the study procedures for a *Shigella* human challenge study have since been published [20], [21], [22]. During the vaccination phase, volunteers were followed as outpatients for safety and completed a surveillance document for 7 days post each vaccination. Solicited and unsolicited adverse events were collected for 28 days after the last vaccination. At the end of the vaccination phase, volunteers who were eligible and willing to proceed with the challenge were admitted to the inpatient unit one day prior to challenge. On the day of challenge, subjects consumed 120 mL of bicarbonate buffer, and immediately after, the freshly prepared challenge inoculum of *S. flexneri* 2a strain 2457T in 30 mL of bicarbonate buffer. Physical assessments were performed daily; vital signs were measured thrice daily. From the day of the challenge and until discharge, all stools passed on the unit were assessed for consistency, weight, and gross blood [19]. Daily stool cultures were performed for qualitative and quantitative measures of the challenge microorganism. Selected colonies were serotyped using commercial agglutination serum (Denka).

Subjects with loose stools were provided oral rehydration and closely monitored for signs and symptoms of hypovolemia and were treated with intravenous fluids as necessary. On or before (if indicated) day 5 post-challenge, subjects were treated with an antibiotic (ciprofloxacin or trimethoprim/sulfamethoxazole) twice daily for 3 days. Subjects were eligible for discharge once they had at least 2 *Shigella*-negative stool cultures and had received at least 2 doses of antibiotics. Volunteers had an outpatient visit one-month post-challenge, and a safety phone call for serious adverse events and adverse events of special interest at 6 months post-challenge. Challenge-related solicited and unsolicited adverse events were collected for 1 month from the day of challenge.

### 2.8 Definitions

The primary clinical endpoint was shigellosis, defined as severe diarrhoea OR moderate diarrhoea with fever or with one or more moderate constitutional or enteric symptom OR dysentery. This definition did not allow for mild diarrhoea. Moderate diarrhoea was defined as 4 to 5 or 401–800 g of loose or watery stools within 24 h. Severe diarrhoea was classified as 6 or more or greater than 800 g of loose or watery stools passed within a 24 h period. More severe diarrhoea was defined as ≥10 or ≥1000 gr of loose or watery stool within a 24 h period. Dysentery was defined as 2 or more loose stools with gross blood (confirmed by hemoccult) within 24 h with any reportable constitutional symptoms. Fever was any confirmed temperature ≥38 °C. Constitutional symptoms include nausea, vomiting, abdominal cramps/pain, myalgia, arthralgia, tenesmus and faecal urgency. Additional definitions are located in the supplement. More severe shigellosis was defined in a post-hoc analysis as at least moderate diarrhoea or dysentery, with fever or severe enteric symptoms. A slight modification of this post-hoc definition was subsequently endorsed for use in *Shigella* CHIMs studies by a convening of experts, held in 2017 [22].

### 2.9 Endpoints

The shigellosis AR after challenge was the primary endpoint. Secondary efficacy endpoints included the number of subjects with moderate to severe diarrhoea, the number with more-severe diarrhoea, the incidence of fever, enteric syndromes of moderate to severe, the number requiring intravenous fluid and early antibiotic treatment, as well as the weight and number of loose stools. In addition, safety and immunogenicity endpoints were assessed (see supplement). The Shigellosis Disease Score described by Porter et al. [23] was used to assess whether the severity of disease experienced by the vaccinees and placebo recipients were comparable. An independent adjudication committee determined the endpoints of each subject.

### 2.10 Immunogenicity assessments

Venous whole blood was collected prior to each vaccination, 7- and 28-days post-vaccination as well as before and 3, 7 and 28 days after challenge. Serum was separated from whole blood and frozen until assayed by ELISA. *S. flexneri* 2a LPS-specific serum IgG antibody titres were determined as previously described [15,24]. A serological responder was defined *a priori* as *a* ≥ 4-fold increase in titre over baseline.

### 2.11 Statistical analysis

Assuming an attack rate for diarrhoea in the placebo group estimated to be 70% and an attack rate (AR) of no higher than 30% in the vaccine group (equivalent to >57% protective efficacy), a total of 28 to 30 subjects per group was chosen to allow for at least 80% power to detect a significant difference (*p*<0.05; lower bound of 95% confidence interval around point estimate of efficacy of > zero) in attack rates between the vaccine and placebo groups. The shigellosis AR was presented as the proportion of subjects per group with shigellosis after challenge. Vaccine efficacy was calculated (VE = (AR_placebo_ – AR_vaccinees_)/(AR_placebo_)*100%) along with exact unconditional 95% confidence intervals (CI). Following the hypothesis that the vaccine reduces the attack rate, at this early stage of the program the effect in only one direction (protective effect) was considered. AR were compared with the unconditional exact 1-sided Barnard test. Analyses were 1-tailed and statistical significance attributed to *p* ≤ 0.05. This was also used to compare the incidence of fever and constitutional/enteric symptoms in the vaccine recipients vs. placebo recipients.

The comparison of the maximum weight of grade 3–5 stools over a 24 h period were conducted by Student's *t*-test of the difference in mean log weight. Comparison of the maximum number of grade 3–5 stools per subject over a 24 h period was by Poisson regression (adjusted for over dispersion). Statistical and data analyses were conducted jointly between investigators from each institution.

The Shigellosis Disease Score was calculated as described previously [23] and was analysed with a 2-sided alpha. Differences in symptoms severity were assessed based on the exact test of equality of row means using modified-ridit scores [25].

Antibody titres were log transformed to normalize the data and summarized as geometric mean titres (GMT) along with 95% confidence intervals. The percentage of subjects reaching a four-fold increase in serum IgG titres compared to baseline was calculated for each group and compared between-groups with Fisher's exact test. Spearman correlation analyses were performed between log_10_-transformed antibody titres and Shigella Disease Score, symptoms, maximum number or weight of loose stools.

### 2.12 Role of the funding source

The funder of the study (The Wellcome Trust) had no role in the study design data collection, data analysis, data interpretation, or writing of the report. The corresponding author had full access to all the data in the study and had final responsibility for the decision to submit for publication.

## 3 Results

### 3.1 Study population

**Fig. 1:**
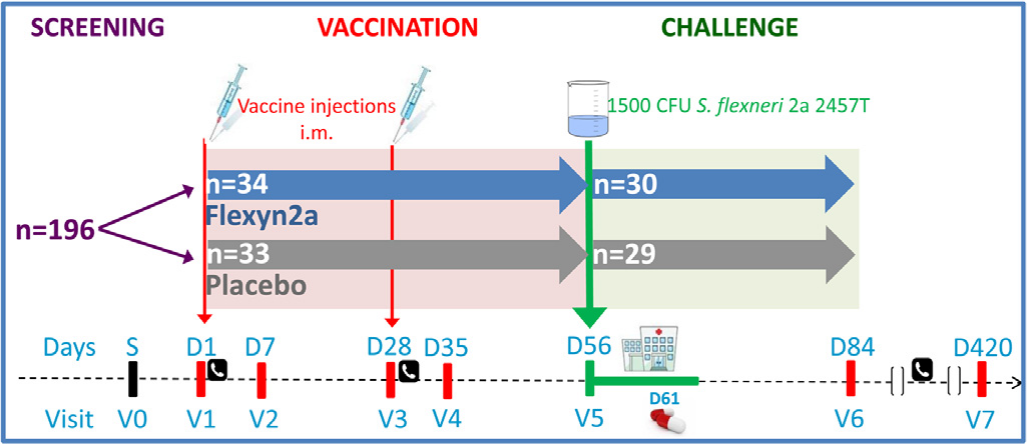
Study Design and Enrolment. Volunteers received 2 doses of the Flexyn2a vaccine or placebo 28 days apart followed by challenge with *S. flexneri* 2a 28 days after the second dose. Immunological assessments were done before and after vaccination and after challenge.

**Fig. 2:**
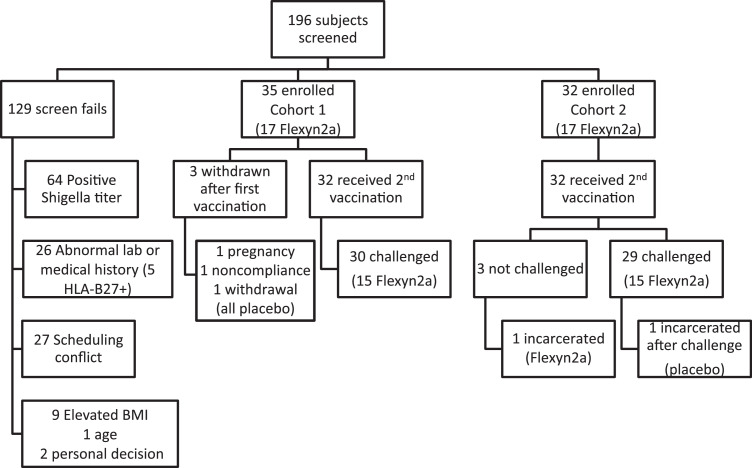
Consort Diagram Study enrolment and subject disposition. BMI= body mass index.

One hundred ninety-six potential volunteers were screened, and 67 volunteers were enrolled (Fig. 1, Fig. 2, Table 1). The most common reason for exclusion was a *S. flexneri* 2a LPS-specific serum IgG titre  ≥2500 at screening (64 volunteers). Following randomization, 34 volunteers received Flexyn2a and 33 received placebo. Out of the planned 60 subjects, 59 were challenged: 30 vaccinees and 29 placebo recipients (Figs. 1,2). Thirty-one percent of the volunteers were women and 80.6% were black or African-American. The median age of participants was 34.8 years (Table 1). The first cohort of 35 received their first vaccination on December 15, 2015, and the second cohort of 32 their first vaccination on March 23, 2016.

**Table 1:** Demographics of enrolled volunteers.

		Vaccination Phase - Safety Population			Challenge Phase - ITT Population		
		Flexyn2a *N* = 34	Placebo *N* = 33	Total *N* = 67	Flexyn2a *N* = 30	Placebo *N* = 29	Total *N* = 59
Gender - *N* (%)	Female	10 (29.4)	11 (33.3)	21 (31.3)	9 (30.0)	8 (27.6)	17 (28.8)
	Male	24 (70.6)	22 (66.7)	46 (68.7)	21 (70.0)	21 (72.4)	42 (71.2)
Age (yrs)	Mean	36.6	34.4	35.5	36.5	34.3	35.4
	Median	35.1	34.5	34.8	35.1	34.5	34.8
	Range	23.5–49.8	22.3–50.3	22.3–50.3	23.5–49.8	22.3–50.3	22.3–50.3
Race - N (%)	American Indian/Alaskan Native, Black or African American*	3 (8.8)	1 (3.0)	4 (6.0)	3 (10.0)	0 (0.0)	3 (5.1)
	Asian	0 (0.0)	1 (3.0)	1 (1.5)	0 (0.0)	1 (3.4)	1 (1.7)
	Black or African American	26 (76.5)	28 (84.8)	54 (80.6)	22 (73.3)	25 (86.2)	47 (79.7)
	Hispanic	1 (2.9)	0 (0.0)	1 (1.5)	1 (3.3)	0 (0.0)	1 (1.7)
	White	4 (11.8)	3 (9.1)	7 (10.4)	4 (13.3)	3 (10.3)	7 (11.9)

*N*= number;%= percent; ITT= intention to treat; yrs.= years.

*These subjects reported mixed race.

### 3.2 Safety

The Flexyn2a vaccine was well-tolerated and the safety data collected generally confirmed the Phase 1 study observations [15]. The most commonly reported adverse events were headache 5/34, 14.7% (95% CI: 5–31.1%) in vaccinees and 10/34, 30.3% (95% CI: 15.6–48.7%) in placebo recipients and pain at the injection site 9/34, 26.5% (95% CI: 12.9–44.4%) in vaccinees and 6/33, 18.2% (95% CI: 7–35.5%) in placebo recipients (Table 2). The majority (75.9%) of the adverse events were of mild intensity. No serious adverse events (SAEs) occurred during this study, and no subjects discontinued participation due to adverse events.

**Table 2:** Adverse events after vaccination (both first and second dose).

Adverse event	Flexyn2a (*n* = 34) Number (%)			Placebo (*n* = 33) Number (%)		
	mild	mod	any	mild	mod	any
Any AEs	14 (41.2)	3 (8.8)	17 (50.0)	8 (24.2)	4 (12.1)	12 (36.4)
Pain/Tenderness at injection site	8 (23.5)	1 (2.9)	9 (26.5)	5 (15.2)	1 (3.0)	6 (18.2)
Erythema/redness at injection site	2 (5.9)	0 (0.0)	2 (5.9)	3 (9.1)	0 (0.0)	3 (9.1)
Induration/swelling at injection site	2 (5.9)	0 (0.0)	2 (5.9)	2 (6.1)	0 (0.0)	2 (6.1)
Headache	4 (11.8)	1 (2.9)	5 (14.7)	6 (18.2)	4 (12.1)	10 (30.3)
Vomiting	1 (2.9)	0 (0.0)	1 (2.9)	2 (6.1)	1 (3.0)	3 (9.1)
Myalgia	2 (5.9)	1 (2.9)	3 (8.8)	0 (0.0)	3 (9.1)	3 (9.1)
Arthralgia	0 (0.0)	0 (0.0)	0 (0.0)	1 (3.0)	1 (3.0)	2 (6.1)
Nausea	3 (8.8)	0 (0.0)	3 (8.8)	0 (0.0)	1 (3.0)	1 (3.0)
Abdominal pain	1 (2.9)	1 (2.9)	2 (5.9)	1 (3.0)	1 (3.0)	2 (6.1)
Chills	2 (5.9)	0 (0.0)	2 (5.9)	1 (3.0)	3 (9.1)	4 (12.1)
Sweats	1 (2.9)	0 (0.0)	1 (2.9)	0 (0.0)	3 (9.1)	3 (9.1)

AE= Adverse event; mod= moderate;%= percent.

### 3.3 Efficacy data

The *S. flexneri* 2a strain 2457T challenge dose administered to the first and the second cohort of volunteers was 1510 and 1707 CFUs, respectively. The shigellosis AR in placebo recipients in cohorts 1 and 2 was similar (60.0% and 64.3%, respectively), with a cumulative AR of 62.1%, consistent with what has been seen in other studies.

**Fig. 3:**
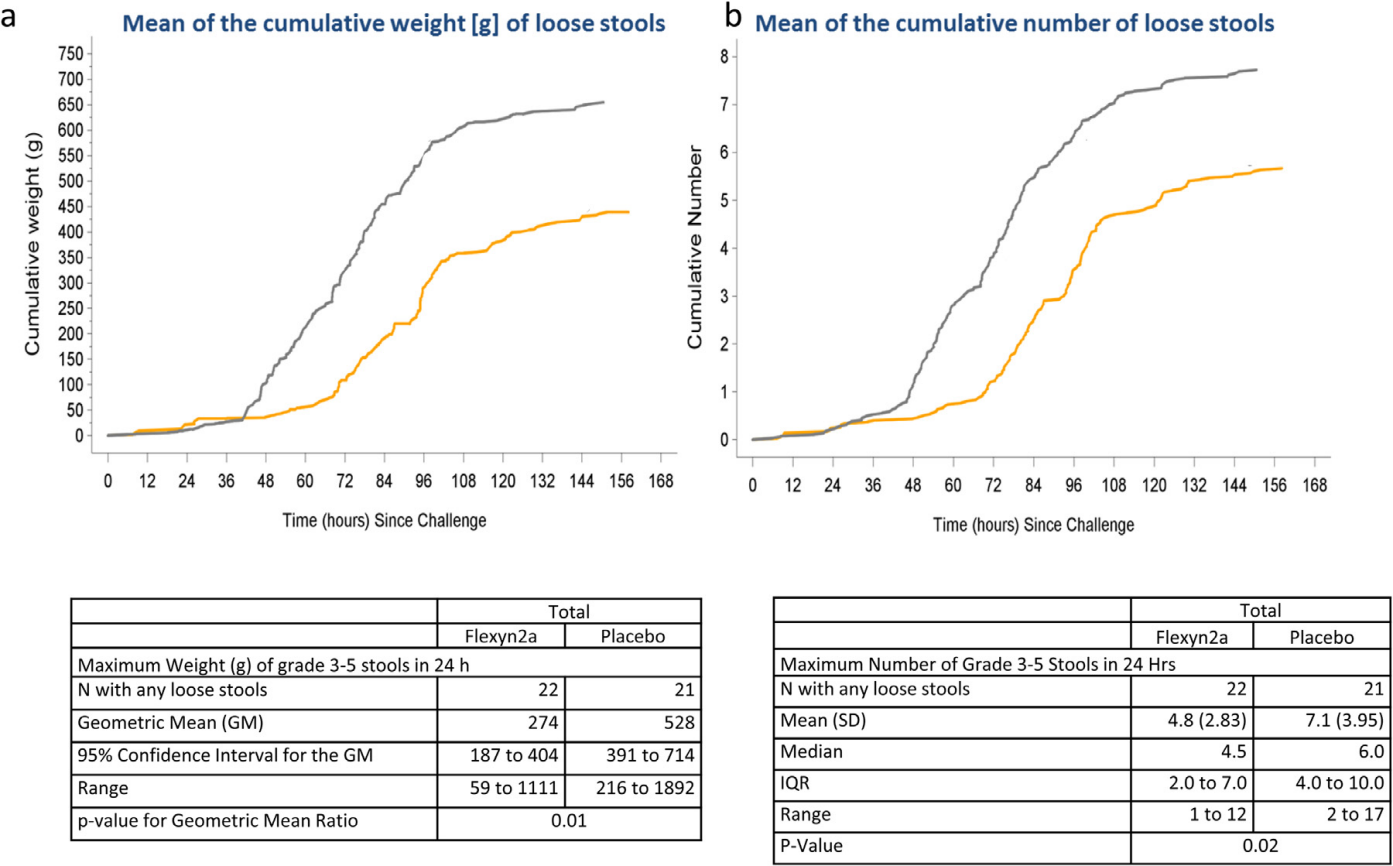
The mean of the cumulative weight (Panel a) and number (Panel b) of loose stools after challenge in the Flexyn2a recipients (yellow line) and the placebo recipients (grey line). The table reports the maximum weight and number of loose stools within any 24 h period after challenge. *N* = number; *g* = grams; h = hours; GM = Geometric mean; IQR = interquartile range; SD = standard deviation (For interpretation of the references to color in this figure legend, the reader is referred to the web version of this article.).

**Fig. 4:**
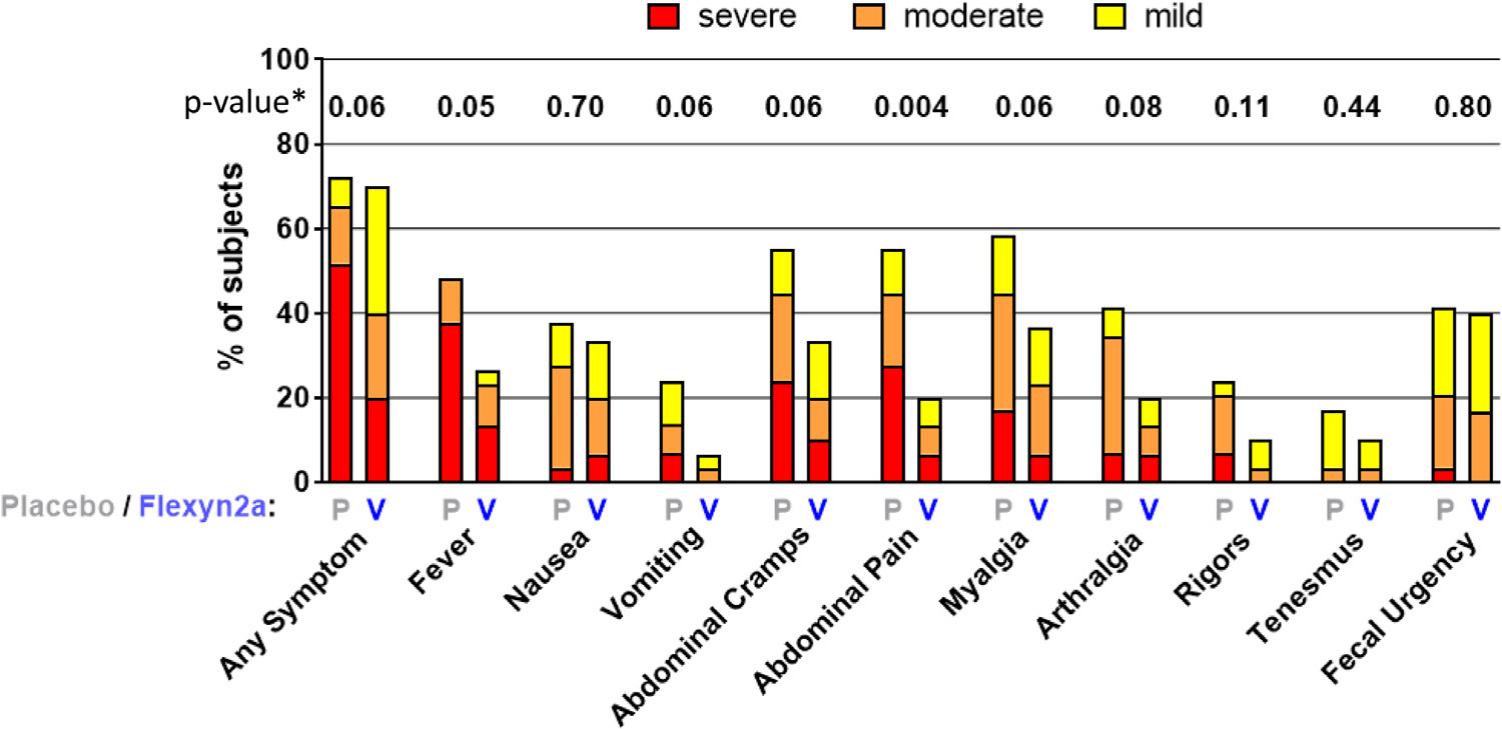
Constitutional and enteric symptoms experienced by volunteers after *Shigella flexneri* 2a challenge. *P*= placebo recipients (*n* = 29); *V*= vaccine recipients (*n* = 30). Colour indicates severity. The p-value for each symptom is along the top and reflects the difference in severity utilizing the modified Ridit Score.

**Fig. 5:**
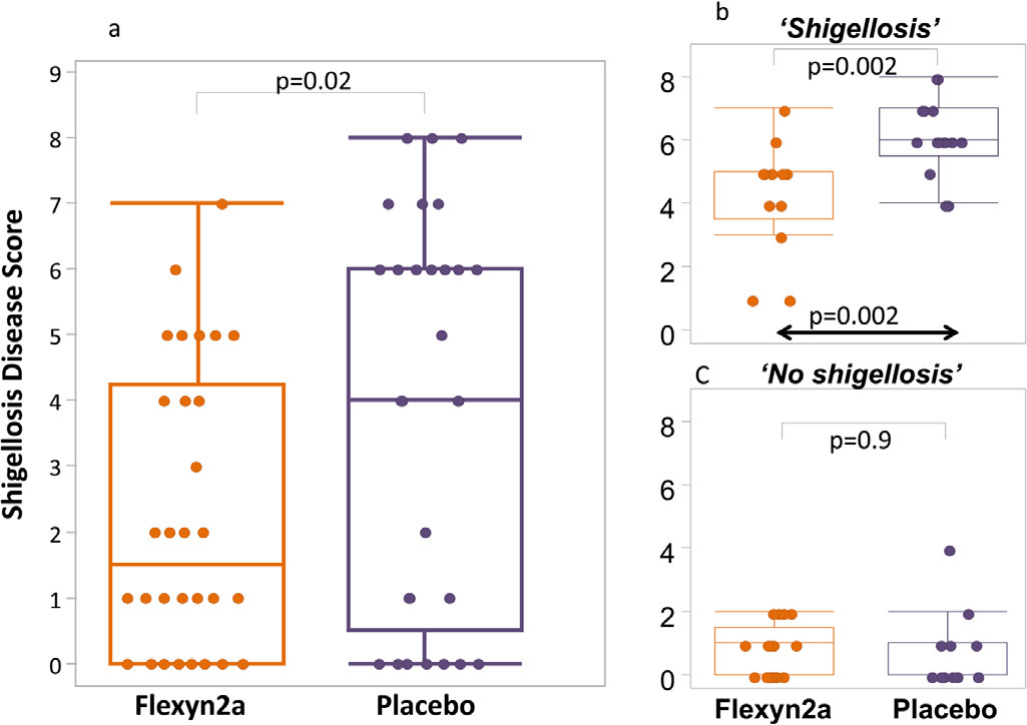
Shigellosis disease severity score in challenged volunteers by Vaccinee or placebo recipient (Panel a), only those that developed shigellosis by vaccinee or placebo recipient (Panel b) and those that didn't develop shigellosis by vaccinee or placebo recipient status (Panel c).

VE was 30.2% against the primary definition of shigellosis (Table 3) (13/30 vs. 18/29; *p* = 0.11; 95% CI −15 to 62.6) and increased to 51.7% against more severe shigellosis (8/30 vs 16/29; *p* = 0.02 95%CI 5.3 to 77.9). In addition, Flexyn2a protected vaccinees against more severe diarrhoea (VE=72%; 2/30 vs 7/29; *p* = 0.07; 95%CI −9.5 to 64.3) and reduced the need for early antibiotic treatment (VE=51.7%; 9/30 vs 18/29; *p* = *p* = 0.01; 95% CI 9 to 76.8) and intravenous fluid administration (VE=47.9%; 7/30 vs 13/29; *p* = 0.05; 95% CI −11.8 to 78.3) (Table 3). Vaccine recipients had lower maximum weight of loose stools in any 24 h period (geometric mean (GM) 274 gm. vs. 528 gm., *p* = 0.009) as well as lower maximum number of loose stools in any 24 h period (mean 4.8 vs. 7.1, *p* = 0.022) (Fig. 3). The incidence of fever or any enteric symptoms was significantly lower in the Flexyn2a group compared with placebo (for any symptom of at least moderate, *p* = 0.044, or severe or greater intensity *p* = 0.011). The severity of key symptoms such as fever, vomiting, abdominal cramps and myalgia (but not nausea, arthralgia, rigors, tenesmus or faecal urgency), was significantly lower among vaccinees (Fig. 4). There was a significant difference (*p* = 0.02) in the Shigellosis Disease Score [23] between the vaccinees (median: 1.6; IQR: 0–4.3) and placebo recipients (median: 4.2; IQQR: 0.5–6.0), even if they met the primary endpoint (Fig. 5a). In addition, vaccinees that met the primary definition of shigellosis had lower disease severity scores than placebo recipients (median score 5, IQR 3.5–5 vs. median score 6, IQR 5.5–7; *p* = 0.002 Fig. 5b). No difference in disease severity score was observed between groups of vaccinees and placebo recipients that did not show symptoms of shigellosis (Fig. 5c).

**Table 3:** Effectiveness of the Flexyn2a vaccine against endpoints.

	Attack Rate N(%)		Vaccine Efficacy	
	Flexyn2a *N* = 30	Placebo *N* = 29	(%)(95%CI)§	p-value*
Shigellosis (primary definition)	13 (43.3)	18 (62.1)	30.2 (−15 to 62.6)	0.11
More Severe Shigellosis (post-hoc definition)	8 (27.6)	16 (53.3)	51.7 (5.3 to 77.9)	0.015
Shigellosis (post-hoc Consensus paper [23] definition)	11 (36.7)	17 (58.6)	37.5 (−9.6 to 64.3)	0.07
Secondary Endpoints				
More Severe diarrhea	2 (6.7)	7 (24.1)	72.4	0.065
Received Early Administration of Antibiotics	9 (30.0)	18 (62.1)	51.7 (9 to 76.8)	0.0093
Received IV Fluids	7 (23.3)	13 (44.8)	47.9 (−11.8 to 78.3)	0.053
Number of subjects with moderate-severe diarrhea	15 (50.0)	17 (58.6)	14.7	0.34
Number of subjects with diarrhea of any severity	17 (56.7)	21 (72.4)	21.7	0.16

Shigellosis: severe diarrhea OR moderate diarrhoea with [fever (oral temperature ≥38 °C) or with one or more moderate constitutional or enteric symptom] OR [dysentery].

More severe shigellosis: defined in a post-hoc analysis as at least moderate diarrhea or dysentery, with fever or severe enteric symptoms.

More severe diarrhea: ≥10 or ≥1000 g loose stools within 24 h.

Severe diarrhea: ≥ 6 or >800 g loose stools within 24 h.

Moderate diarrhea: 4 to 5 or 401–800 g loose stools within 24 h.

Dysentery: at least 2 loose stools with gross blood (confirmed by hemoccult) within 24 h and any reportable constitutional symptom.

Constitutional/Enteric Symptoms: nausea, vomiting, abdominal cramps/pain, myalgia, arthralgia, rigors, tenesmus and faecal urgency.

§ Exact unconditional 95% confidence interval for vaccine efficacy.

⁎ Unconditional exact 1-sided Bernard test; analyses were 1-tailed and statistical significance attributed to *p* ≤ 0.05. Abbreviations: *N*= Number, CI= Confidence interval.

### 3.4 Challenge strain shedding

Forty-three of 59 volunteers shed the *S. flexneri* 2a 2457T challenge strain: 22 (73%) placebo recipients and 21 (72%) Flexyn2a recipients. The CFUs detected in the stools of shedding subjects was similar in both groups, with a mean value of 6.20 (95% CI 5.68 to 6.72) and 5.79 (5.27 to 6.31) log_10_CFU/g of stool in Flexyn2a and placebo, respectively (Table 4). The 13 vaccinees and the 18 placebo recipients adjudicated as having shigellosis all shed the challenge organism with similar levels (about 7 and 6 log_10_ *Shigella* CFU/g of stool, respectively) except for 1 vaccinee with no shedding detected.

**Table 4:** *S. flexneri* 2a shedding after challenge in stools.

	Cohort 1		Cohort 2		Total	
	Flexyn2a *N* = 15	Placebo *N* = 15	Flexyn2a *N* = 15	Placebo *N* = 14	Flexyn2a *N* = 30	Placebo *N* = 29
Number (%) of subjects with detectable CFU	9 (60)	10 (67)	12 (80)	10 (71)	21 (70)	20 (69)
Mean (SD) log_10_ cfu/g	6.89 (0.74)	5.63 (1.80)	5.69 (1.88)	5.96 (1.36)	6.20 (1.59)	5.79 (1.56)
95% Confidence Interval for the Mean	6.32 to 7.46	4.34 to 6.91	4.49 to 6.88	4.98 to 6.93	5.48 to 6.92	5.06 to 6.52

Postchallenge stools were cultured daily until challenge organism was cleared.^20^ For quantitative cultures, measured amounts of stool are serially diluted in sterile saline and plated on MacConkey agar and a selective media to determine the CFU's of the challenge strain per gram of stool.

*N*=number;%= percent; CFU= colony forming unit; SD= standard deviation.

### 3.5 Serological responses

**Fig. 6:**
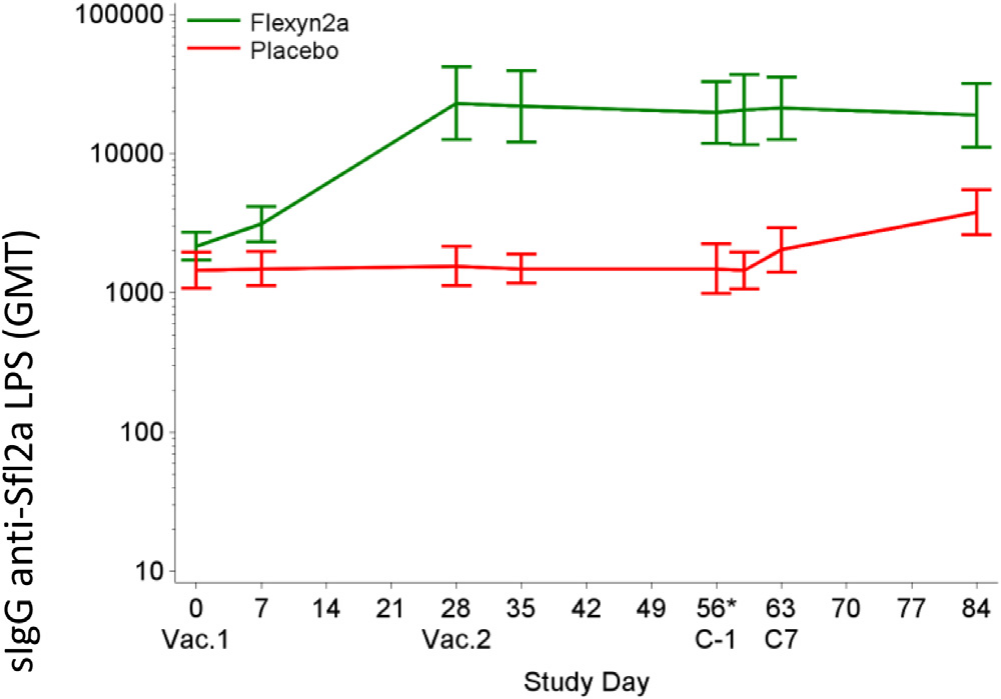
Shows the anti-*S. flexneri* 2a LPS serum IgG antibody titres by study day. Vac= Vaccination. *C*-1 = 1 day prior to challenge. C7= 7 days post-challenge.

A 4-fold or greater rise in serum IgG titres directed to *S. flexneri* 2a LPS was seen in 76.5% of vaccinees after the first dose, which increased to 81.8% after the second vaccination (Fig. 6, Table 5). Despite randomization, the baseline serum IgG titres directed to *S. flexneri* 2a LPS were slightly higher (1.5-fold, *p* = 0.04) in the Flexyn2a recipients than in the placebo recipients (Fig. 6, Table 5). *S. flexneri* 2a LPS-specific serum IgG responses increased from a baseline GMT of 2172 (95% CI 1722–2740) to 23,119 (95% CI 12,704–42,073) on day 28 after the first vaccination with Flexyn2a (Fig. 6, Table 5). Neither the second vaccination (GMT 19,896 on day 55; 95% CI 11,951–33,124), nor the subsequent challenge with *S. flexneri* 2a (GMT 18,958 on day 84; 95% CI 11,164–32,192) increased the LPS-specific serum IgG titre. Placebo recipients had baseline levels of S. *flexneri* 2a LPS-specific serum IgG prior to challenge with a GMT of 1459 (95% CI 1079–1973). The LPS-specific serum IgG increased to 3805 (2609–5551) one month after challenge. The level of *S. flexneri* 2a LPS-specific serum IgG elicited following vaccination was 5.0-fold higher than placebo recipients following challenge (*p*< 0.0001). A similar response was observed for *S. flexneri* 2a LPS-specific serum IgA responses following vaccination, confirming the Phase 1 study results [15] and data are included in the accompanying Clarkson, et al. manuscript which focuses on the immunological results.

**Table 5:** Anti-LPS Serum IgG titres in vaccine and placebo recipients.

	**Flexyn2a**				Placebo			Ratio GM Flexyn2a/Placebo	
Study Day	N	Median (Range)	GM (95% CI)	% Responder (95% CI)	N	Median (Range)	GM[5] (95% CI)	Ratio (95% CI)	P-Value
Vaccination Phase									
Day 0	34	3200 (800–6400)	2172 (1722 – 2740)	N/A	30	1600 (200 - 6400)	1459 (1079 - 1973)	1.5 (1.0 - 2.2)	0.04
Day 7	33	3200 (800 - 25,600)	3133 (2341 - 4193)		30	1600 (200 - 6400)	1493 (1125 - 1981)	2.1 (1.4 - 3.1)	0.0004
Day 28	34	25,600 (800 - 409,600)	23,119 (12,704 - 42,073)	76.5% (58.83% – 89.25%)	30	1600 (200 - 6400)	1563 (1125 - 2173)	14.8 (7.5 - 29.0)	<0.0001
Day 35	32	25,600 (1600 - 409,600)	21,998 (12,180 - 39,731)		30	1600 (400 - 6400)	1493 (1176 - 1896)	14.7 (7.8 - 27.7)	<0.0001
Day 55 (prechallenge)	33	25,600 (800 - 204,800)	19,896 (11,951 - 33,124)	81.8% (64..54 — 93.02)	30	1600 (200 - 102,400)	1493 (991 - 2249)	13.3 (7.0 - 25.3)	<0.0001
Challenge Phase									
Day 59 (3 days postchallenge)	30	25,600 (800 - 409,600)	20,794 (11,627 - 37,188)		29	1600 (200 - 6400)	1454 (1072 - 1973)	14.3 (7.5 - 27.3)	<0.0001
Day 63 (7 days postchallenge)	30	25,600 (1600 - 409,600)	21,280 (12,628 - 35,857)		28	1600 (200 - 6400)	2049 (1419 - 2959)	10.4 (5.6 - 19.4)	<0.0001
Day 84	30	25,600 (1600 - 409,600)	18,958 (11,164 - 32,192)		28	3200 (800 - 25,600)	3805 (2609 - 5551)	5.0 (2.6 - 9.4)	<0.0001

The anti-LPS serum IgG titres (median, range, geometric mean (GM) and 95% confidence interval (95% CI) by vaccinee or placebo recipient status, and the ratio of the titre of the vaccinees/placebo recipients. The percent (%) responder by serum IgG (4-fold or greater increase in serum IgG) titre after vaccination. *N*=number.

The ratios of GMT and corresponding 95% CI's between treatment groups were compared by Student's *t*-test of log_10_-transformed values.

### 3.6 Correlate of protection analyses

**Fig. 7:**
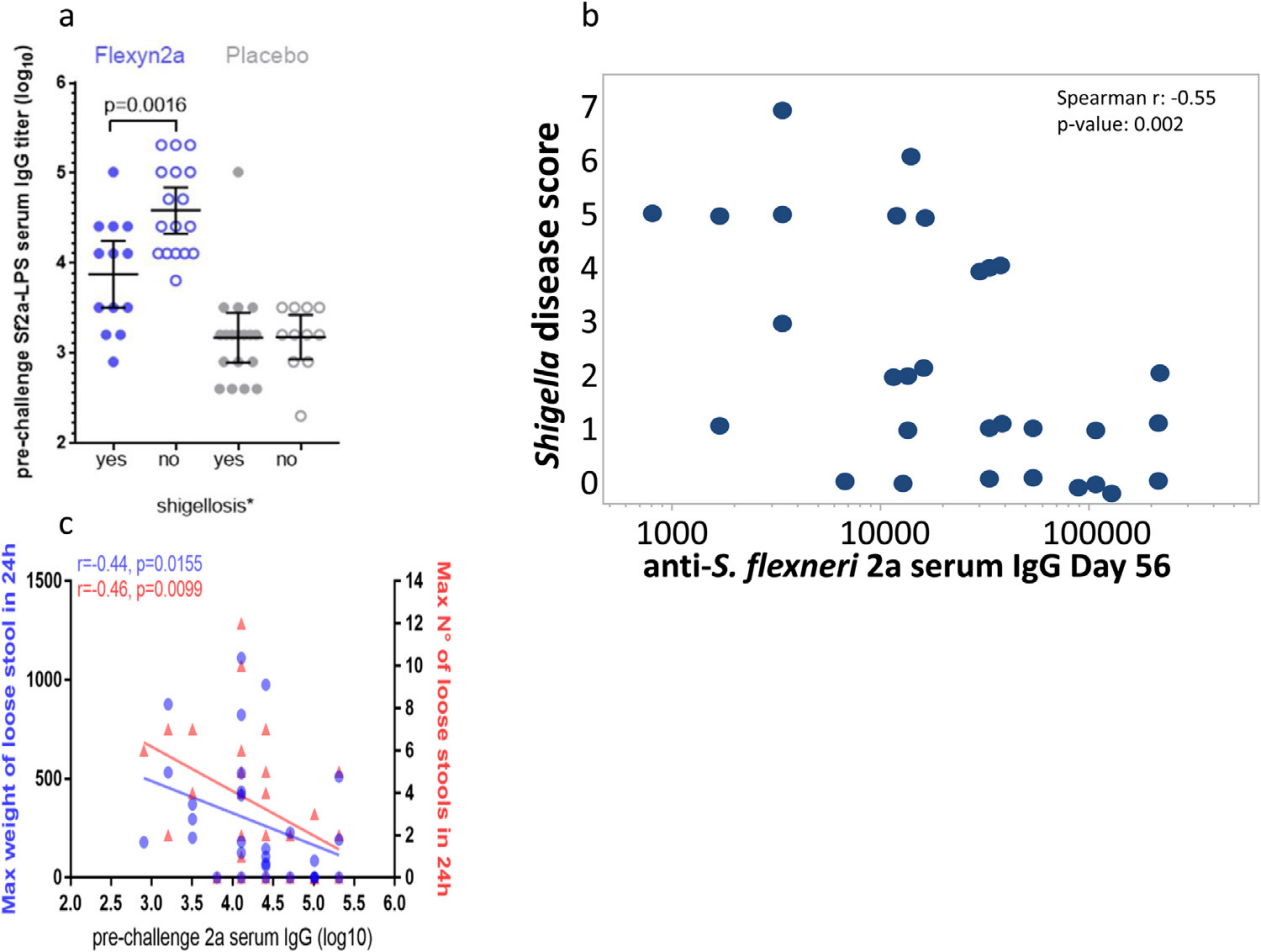
Panel a shows the prechallenge serum IgG to *S. flexneri* 2a LPS in the vaccine and placebo recipients broken down by whether they met the primary objective of Shigellosis. Panel b, the relationship between pre-challenge anti-LPS serum IgG to Shigella disease severity score is demonstrated for the recipients of the Flexyn2a vaccine. Panel c demonstrates the relationship of the pre-challenge anti-LPS serum IgG to maximum stool weight (blue circles) and number (red triangles) in 24 h (For interpretation of the references to color in this figure legend, the reader is referred to the web version of this article.).

In the Flexyn2a group, vaccinees protected from shigellosis had 5.1-fold higher *S. flexneri* 2a -LPS-specific serum IgG GMTs at time of challenge compared to vaccinees developing shigellosis (*p* = 0.002) (Fig. 7a).

Consistent with these findings, subjects with increasing LPS-specific serum IgG titres at the time of challenge were inversely correlated with the Shigellosis Disease Score (Spearman *R*=−0.55, 95% CI −0.76 to −0.23; *p* = 0.002) (Fig. 7b), loose stool weight (Spearman *R*=−0.44, 95% CI −0.69 to −0.08; *p* = 0.016) and number of loose stools (Spearman *R*=−0.46, −0.70 to −0.12; *p* = 0.01) (Fig. 7c).

## 4 Discussion

The results of our trial indicate that the bioconjugate Flexyn2a candidate vaccine is safe, induces a robust serologic response to *S. flexneri* 2a LPS and provides partial protection against shigellosis in a controlled human infection model. Although the target vaccine efficacy of 57% percent was not achieved in the per protocol analysis, the vaccine did significantly ameliorate symptoms and disease. In this setting, Flexyn2a is more efficacious against severe shigellosis. Following a recent publication by McLennan et al.*,* [22] VE was also calculated using the published “shigellosis-consensus-definition in challenge trials”. This definition included moderate or severe diarrhoea or dysentery with fever or a moderate or severe enteric symptom. Using this definition in a post-hoc analysis, the VE was 37.5% (11/30 vs 17/29; *p* = 0.07; 95%CI −9.6 to 64.3). This definition was not available at the time of this study design, and in fact, this study was used in the development of the consensus definition.

In addition, to the Flexyn2a vaccine being more effective at preventing severe shigellosis, placebo recipients had higher diarrheal stool outputs, more severe and frequent clinical symptoms including fever, were treated earlier with antibiotics and tended to have greater needs for intravenous fluids for dehydration than did the Flexyn2a group. The Flexyn2a recipients also had a lower overall shigellosis disease severity score than the placebo recipients, even if they developed shigellosis, indicating that the breakthrough cases in vaccinees were milder.

As previously demonstrated with other conjugate vaccines, parenteral vaccination can be efficacious against invasive disease and has the potential to protect against mucosal pathogens [26,27]. Also, in this human challenge model, the degree of vaccine efficacy is greater in more severe outcomes compared to milder infections; the latter may require a more robust immune response. When considering a vaccine for travellers, or a vaccine to prevent hospitalization of children, prevention of severe disease is an important outcome and may lead, as shown in this study, to a reduced need for antibiotic intervention, which would add greater public health benefit to vaccine use.

Previous studies have demonstrated that challenge with *Shigella* elicits an immune response that confers protection against a subsequent homologous challenge [28], [29], [30]. These results highlight the potential of the LPS-specific serum IgG immune response in achieving protection and reducing severity of disease, which is consistent with previous reports of the relevance of LPS-specific serum IgG as a marker of protection against shigellosis [31], [32], [33], [34]. A number of other mucosal and systemic immunity measures correlated with LPS-specific serum IgG responses and protection in this study. Those results are presented in the accompanying Clarkson et al. manuscript.

This study, like others utilizing human challenges, has both strength, as well as limitations. The limitations include a limited sample size, and that the study population (eg, immunologically naïve adults) is not representative of the target population, namely, children in low and middle income countries. Similarly, volunteers differ from the general population in regards to race and sex. Despite these limitations, this study afforded an early efficacy evaluation of the Flexyn2a vaccine against *S. flexneri* 2a. It also allowed for in depth microbiological and immunological assessments that are impractical in a field study, and especially in the target population.

This study presents proof-of-concept that the Flexyn2a bioconjugate vaccine is able to protect against severe shigellosis outcomes following challenge, induce mucosal immunity and elicit a robust LPS-specific serum IgG immune response which correlates with protection against shigellosis. For a broad impact on public health, the *Shigella* vaccine will have to protect against the most prevalent *Shigella* serotypes causing disease in young children in low to middle income countries. A tetravalent bioconjugate is currently being tested in a phase 1/2 clinical trial in Kenya (NCT04056117). This study is investigating the safety and immunogenicity of the tetravalent vaccine in an age de-escalating manner down to nine months of age.

## Declaration of Competing Interest

CA, PM, AMD, RF and VGF were employees of LimmaTech Biologics at the time of the study. KRT, CA, PM, ALB, AMD, RWK, SC, KAC, JB, RF, BD, HW, BF, JH, DS, VGF received grant support from the Wellcome Trust. The authors declare no other competing interests.

## Appendix Supplementary materials

Image, application 1

Image, application 2

Image, application 3
