# Supplementary material for: Human challenge study with a Shigella bioconjugate vaccine: Analyses of clinical efficacy and correlate of protection
Source: eBioMedicine. 2021 Apr 13;66:103310. doi: 10.1016/j.ebiom.2021.103310 (PMC8054157; doi:10.1016/j.ebiom.2021.103310)
Supplement: Supplementary file 1 [file mmc1.docx]

Supplemental Material

Table of Contents

[List of Investigators: 2](#_Toc63373089)

[Definitions 3](#_Toc63373090)

[Secondary endpoints 3](#_Toc63373091)

[Inclusion and Exclusion Criteria 4](#_Toc63373092)

# List of Investigators:

Principal Investigator: Kawsar Talaat, MD

Study Manager: Cristina Alaimo

Study Director: Patricia Martin, PhD

Epidemiology: Chad Porter, PhD, MPH

Immunology (WRAIR): Robert Kaminski, PhD

Kristen Clarkson

Sub-Investigators JHU: Jane Halpern, MD

Daniel Elwood, RN, CCRC

Brittany Feijoo, MSN, FNP-BC

Barbara DeNearing, RN, BSN, CCRC

Microbiology and Immunology (JHU): Subhra Chakraborty, PhD

1. Louis Bourgeois, PhD, MPH

David Sack, MD

Jessica Brubaker

Subinvestigators LimmaTech: Veronica Gambillara Fonck, PhD

Anita Dreyer

Rahel Fröhlich

CRO: The Emmes Corporation

Research Monitor: Capt. Mark S. Riddle, MD DrPH

# Definitions

**Shigellosis** was defined as (severe diarrhea) OR (moderate diarrhea with (fever or with one or more moderate constitutional or enteric symptom) OR (dysentery).

**More severe diarrhea**: ≥10 or ≥1000g loose stools within 24 hours.

**Severe diarrhea**: ≥ 6 or >800g loose stools within 24 hours.

**Moderate diarrhea**: 4 to 5 or 401-800g loose stools within 24 hours.

**Mild diarrhea** was defined as ≥2 loose stools weighing ≥200g within 48 hours or 1 loose stool weighing ≥300g and <401g.

**Dysentery**: at least two loose stools with gross blood (confirmed by hemoccult) within 24 hours and any reportable constitutional symptom.

**Fever**: a measured oral temperature ≥38°C confirmed within 20 min.

**Constitutional/Enteric Symptoms**: nausea, vomiting, abdominal cramps/pain, myalgia, arthralgia, rigors, tenesmus and fecal urgency.

**More severe shigellosis was defined in a post-hoc analysis as at least moderate diarrhea or dysentery, with fever or severe enteric symptoms.**

# Secondary endpoints

**Efficacy endpoints:**

Shigellosis Disease score

Number of subjects with moderate to severe diarrhea

Number of subjects with more-severe diarrhea (defined as ≥10 loose (grade 3-5) stools within 24h or ≥1000 gr loose (grade 3-5) stools within 24h)

Number of subjects with fever

Number of subjects with diarrhea of any severity

Mean and medians of total weight of grade 3-5 stools passed per subject

Mean and median number of grade 3-5 stools per subject

Number of subjects with any constitutional or enteric symptoms rated as moderate to severe

Number of subjects who indicate they would have reduced their daily activity if they had been vacationing or traveling for business because of their Shigella illness

Mean and median time from challenge to onset of shigellosis

Number of cfu’s of the challenge strain per gram of stool

Number of subjects requiring early antibiotic treatment

Number of subjects requiring IV fluids

Mean and median duration of diarrhea

Highest recorded febrile temperature

Number of stools with blood

**Immunogenicity endpoint:**

To further assess the systemic and mucosal immunogenicity of the vaccine the level of anti 2a-serum IgG and anti 2a-stools IgA will be compared between vaccine and placebo.

# Inclusion and Exclusion Criteria

**Inclusion Criteria:**

1. Male or female age 18-50 years (inclusive)

2. Good health, without clinically significant medical history or physical examination findings.

3. Negative serum pregnancy test at screening, and negative urine before each vaccination and before challenge for female subjects of childbearing potential.

4. Females of childbearing potential must agree to avoid pregnancy by use of effective contraception. Abstinence is not acceptable as effective contraception. Female subjects unable to bear children must have this documented (e.g. tubal ligation or hysterectomy).

5. Willingness to participate in the study after all aspects of the protocol have been explained and written informed consent obtained.

6. Completion of a training session and demonstrated comprehension of the protocol procedures, knowledge of Shigella‐associated illness, and by passing score of 70% or better on a written examination (comprehension test).

7. Availability for the study duration, including all planned follow‐up visits.

8. Willingness to refrain from participating in other studies of investigational products until completion of the last study visit.

**Exclusion Criteria:**

1. Women currently nursing.

2. Presence of a significant medical or psychiatric condition which in the opinion of the investigator precludes participation in the study.

3. Clinically significant abnormalities in screening hematology or serum chemistry as determined by PI or PI in consultation with the research monitor and sponsor.

4. Presence in the serum of HIV antibody, HBs‐Ag, or HCV antibody (if confirmed positive by HepC confirmatory test, i.e. RIBA, PCR)

5. Evidence of IgA deficiency (serum IgA < 7 mg/dl or limit of detection of assay).

6. Evidence of current excessive alcohol consumption or drug dependence.

7. Evidence of impaired immune function.

8. BMI <19 and ≥35

9. Recent vaccination or receipt of an investigational product (within 30 days before vaccination or until last study visit)

10. Personal history of an inflammatory arthritis.

11. Positive blood test for HLA‐B27 antigen.

12. Personal history of irritable bowel syndrome as defined by Rome III criteria.

13. Treatment with immunoglobulins or blood products within 3 months from first candidate vaccine injection.

14. Regularly abnormal stool pattern (fewer than 3 per week or more than 3 per day) or loose or liquid stools

15. Regular use of laxatives, antacids, or other agents to lower stomach acidity.

16. Use of any medication known to affect the immune function (e.g., systemic steroids) within 30 days preceding the first vaccination or planned use during the entire study period.

17. Known allergy to any of the following antibiotics: ciprofloxacin, trimethoprim-sulfamethoxazole or penicillin.

18. Symptoms consistent with Traveler’s Diarrhea concurrent with travel to countries where Shigella infection is endemic (most of the developing world) within two years prior to dosing, OR planned travel to endemic countries during the length of the study.

19. Vaccination for or ingestion of Shigella within 3 years prior to vaccination

20. Use of antibiotics during the 7 days before vaccination and challenge

21. Use of proton pump inhibitors, H2 blockers or antacids within 48 hours prior to challenge.

22. Serum IgG titer to *Shigella flexneri* 2a LPS ≥ 2500

23. Current occupation involving handling of Shigella bacteria

24. History of allergy to any vaccine or to soy

25. Any other criteria which, in the investigator’s opinion, would compromise the ability of the subject to participate in the study, the safety of the study, or the results of the study
