# Supplementary material for: Human challenge study with a Shigella bioconjugate vaccine: Analyses of clinical efficacy and correlate of protection
Source: eBioMedicine. 2021 Apr 13;66:103310. doi: 10.1016/j.ebiom.2021.103310 (PMC8054157; doi:10.1016/j.ebiom.2021.103310)
Supplement: Supplementary file 2 [file mmc2.pdf]

1.0 TITLE PAGE

T R I A L   P R O T O C O L

Title	Phase 2b, double-blind, placebo-controlled efficacy challenge study with a candidate bioconjugate vaccine against *Shigella flexneri* 2a
Sponsor	LimmaTech Biologics AG
Sponsor's Address	Grabenstrasse 3, CH-8952 Schlieren, Switzerland
Study Director	Patricia Martin, PhD<br>Clinical Director<br>LimmaTech Biologics AG<br>Phone: +41 44 733 85 63<br>Fax: +41 44 733 85 74<br>patricia.martin@lmtbio.com
Principal Investigator	Kawsar R. Talaat, M.D.<br>Assistant Scientist, International Health<br>Center For Immunization Research<br>Johns Hopkins Bloomberg School of Public Health<br>624 North Broadway, Room 249<br>Baltimore, MD 21205<br>Phone: +1- 410-502-9627<br>Fax: +1- 410-502-6898<br>ktalaat@jhu.edu
Phase	2b
IND Number	16242
Date of Current Version	V5 of 7 February 2017
Previous version	V4 of 25 January 2016

CONFIDENTIAL

This document contains information which is the property of LimmaTech Biologics AG, Switzerland and therefore is provided to you in confidence for review by you, your staff, an applicable Institutional Ethics Committee / Institutional Review Board and Regulatory Authorities. It is understood that this information will not be disclosed to others without written approval from LimmaTech Biologics Ag, except to the extent necessary to obtain informed consent from those persons (or their parents / legal guardian) to whom the drug may be administered.

1.1 General administrative information

Sponsor:	**LimmaTech Biologics AG**<br>Grabenstrasse 3<br>CH-8952 Schlieren, Switzerland<br>Phone: +41-44-733-8585<br>Fax: +41-44-733-8576
Study Manager	**Cristina Alaimo**<br>Clinical Project Manager<br>LimmaTech Biologics AG<br>Grabenstrasse 3<br>CH-8952 Schlieren, Switzerland<br>Phone: +41-44-733-8577<br>Fax: +41-44-733-8576<br>Email: cristina.alaimo@lmtbio.com
Study Director	**Patricia Martin, PhD**<br>Clinical Director<br>LimmaTech Biologics AG<br>Phone: +41 44 733 85 63<br>Fax: +41 44 733 85 74<br>Email: patricia.martin@lmtbio.com
CRO	The **EMMES Corporation**<br>401 North Washington St. Suite 700<br>Rockville, MD 20850<br>United -States<br>Phone: +1-301-251-1161<br>Fax: +1-301-251-1355<br>Email: limma@emmes.com
Principal Investigator	**Kawsar R. Talaat, M.D.**<br>Assistant Scientist, International Health<br>Center For Immunization Research<br>Johns Hopkins Bloomberg School of Public Health<br>624 North Broadway, Room 249<br>Baltimore, MD 21205<br>Phone: +1- 410-502-9627<br>Fax: +1- 410-502-6898<br>Email: ktalaat@jhu.edu
Clinical Trial Sites	Center for Immunization Research (CIR) Isolation Unit<br>301 Building, 301 Mason Lord Drive Suite 4300<br>Baltimore, MD 21224<br><br>CIR Outpatient Clinic<br>624 N. Broadway, Hampton House Rm. 117<br>Baltimore, MD 21205

Safety Laboratory	**Quest Diagnostics**<br>1901 Sulphur Spring Road<br>Baltimore, MD 21227<br>410-247-0001 (phone)<br><br>Johns Hopkins Medical Laboratories<br>600 N. Wolfe Street / Meyer B-130<br>Baltimore, MD 21287-7070<br>Fax: (410) 614-0455<br><br>Johns Hopkins Bayview Medical Center Lab<br>4940 Eastern Ave. Baltimore, MD, 21224
Immunology Laboratory	**Robert Kaminski, PhD**<br>Immunology Group, Department of Subunit Enteric Vaccine and Immunology (SEVI)<br>Bacterial Diseases Branch (BDB)<br>Walter Reed Army Institute of Research (WRAIR)<br>Phone: +1-301-319-9803<br>Fax: +1-301-319-9801<br>Email : robert.w.kaminski.civ@mail.mil
Epidemiology	**Chad Porter, PhD, MPH**<br>Head, Clinical Studies and Epidemiology Branch<br>Enteric Diseases Department<br>Naval Medical Research Center<br>Office: 301-319-7505<br>Mobile: 240-997-5728<br>Fax: 301-319-7679<br>Email: chad.k.porter2.civ@mail.mil
Site Investigational Product Accountability (Pharmacy)	**Johns Hopkins Hospital Research Pharmacy**<br>600 N. Wolfe St., Osler 100<br>Baltimore, MD 21287<br>fax # 410-614-8074; phone# 410-955-6337<br>email: hkim7@jhmi.edu
Alternative Pharmacy	**Johns Hopkins Bayview Medical Research**<br>4940 Eastern Ave. Baltimore, MD 21224<br>AA Building, Room 149<br>Telephone: 410-550-2747<br>Email: abeselm1@jhmi.edu
Site Investigational Product Accountability (Challenge Strain)/ Microbiology lab	CIR/GDEC Enterics Research Laboratory (JHSPH)<br>615 N. Wolfe St. / W5620/5609/5614<br>Baltimore, MD 21287

List of Sub-investigators

CIR, Johns Hopkins Bloomberg School of Public Health (JHBSPH), 624 North Broadway, Baltimore, MD 21205

Jane Halpern, MD	Investigator<br>E-mail: jhjp@comcast.net
Dan Elwood, RN, CCRC	Senior Research Nurse, Coordinator<br>Phone: 410-908-1419<br>Email: delwood@jhu.edu
Brittany Feijoo FNP-BC	Nurse Practitioner<br>Phone: 443-478-6668<br>Email: bjohns71@jhu.edu
Barbara DeNearing, RN, BSN, CCRC	Senior Research Nurse<br>Phone: 410-935-6938<br>E-mail: bdenear1@jhu.edu

Enteric Microbiology and Immunology Laboratory, Global Disease Epidemiology and Control Program, Rm W 5620, JHBSPH, 615 N. Wolfe Street, Baltimore MD 21205

Subhra Chakraborty; PhD	Assistant Scientist<br>Phone: 410-502-1622<br>Fax: 410-502-6733<br>E-mail: Schakr11@jhu.edu
A. Louis Bourgeois, PhD, MPH	Senior Research Scientist<br>Phone: +1-410-614-1860<br>Fax: +1-410-502-6733<br>E-mail: Lbourge1@jhu.edu
David Sack, MD	Professor<br>Phone: +1-443-287-8795<br>Fax: +1-410-502-6733<br>Email: dsack@jhu.edu

Research monitor

Enteric Disease Department (EDD), Naval Medical Research Center (NMRC)
503 Robert Grant Avenue, Silver Spring, MD 20910

**CAPT Mark S. Riddle, MD DrPH**	Phone: +1-301-319-7686<br>Fax: +1-301-319-7679<br>Email: mark.s.riddle10.mil@mail.mil

1.2 Signature pages

Principal Investigator Signature Page

I, the undersigned, have reviewed this protocol, including all Appendices and I will conduct the clinical study as described and will adhere to GCP/ICH and all the ethical and regulatory considerations stated. I confirm to have read and understood the contents of the Investigator Brochure of the investigational product.

Kawsar Talaat

Digitally signed by Kawsar Talaat
DN: cn=Kawsar Talaat, o=Johns Hopkins Bloomberg School of Public Health, ou=Center for Immunization Research, email=ktalaat@jhu.edu, c=US
Date: 2017.03.10 10:07:11 -05'00'

Kawsar R. Talaat, M.D.
Principal Investigator

10-MAR-2017

Date of signature (DD-MMM-YYYY)

Sponsor Signature Page

I have read/written the protocol and confirm that the protocol follows the current GCP guidelines.

____

Patricia Martin, PhD
Clinical Director

____

Date of signature (DD-MMM-YYYY)

____

Cristina Alaimo, PhD
Senior Clinical Project Manager

____

Date of signature (DD-MMM-YYYY)

2.0 SYNOPSIS

**Protocol Title**	Phase 2b, double-blind, placebo-controlled efficacy challenge study with a candidate bioconjugate vaccine against Shigella flexneri 2a
**Trial Phase**	Phase 2b
**Name of the investigational product**	*Shigella flexneri* 2a bioconjugate vaccine: Flexyn2a
**Study Site**	Center for Immunization Research (CIR)<br>Department of International Health (DIH)<br>Johns Hopkins Bloomberg School of Public Health (JHSPH)<br>624 N. Broadway, Hampton House, Room 117<br>Baltimore, MD 21205<br><br>Center for Immunization Research (CIR) Isolation Unit<br>301 Building, 301 Mason Lord Drive Suite 4300<br>Baltimore, MD 21224
**Principal Investigator**	Dr. Kawsar Talaat, MD
**Investigational Product description**	O antigen-polysaccharide of *S. flexneri* 2a conjugated to the recombinant Pseudomonas aeruginosa Exoprotein A, rEPA
**Study Timelines**	Planned screening/enrollment: 3 months per cohort<br>Vaccination period: 2 months<br>Challenge period: approximately 10 days in-patient observation followed by about 1 month outpatient follow up<br>Post-study safety-follow up call: 6 months from challenge<br>Total study duration: about 8 to 10 months
**Vaccine dose and administration**	The vaccine (0.5 ml per injection) will be administered using a dose of 10 ug polysaccharide antigen and 50 ug EPA. The placebo group subjects will receive saline solution (0.5 ml per injection).<br>Each subject will receive a total of two intramuscular injections, one on D0 and one on D28.
**Study Population**	Healthy subjects (male or female) of all ethnicities, aged 18-50 years (inclusive)
**STUDY DESIGN**	Single-center, double-blind, placebo-controlled, phase 2b vaccination and challenge study designed to assess the protective efficacy of *Shigella flexneri* 2a-EPA bioconjugate candidate vaccine, as well as collect additional safety and immunogenicity data.<br>Two groups of about 36 subjects will be enrolled successively, and up to 30 subjects from each group will be consecutively challenged.<br><br>In each group two phases will be carried out: an initial vaccination phase followed by a challenge phase.
**Vaccination phase**	In the vaccination phase, subjects will be randomized 1:1 to receive either the investigational vaccine or placebo on an out-patient basis. Vaccine and placebo preparations will be given intramuscularly on Days 0 and about 28 (visit V1, V3). After each vaccination, subjects will be followed as out-patients for safety using memory aid surveillance.<br><br>In the subsequent challenge phase each group (with up to 30 subjects per group) will be admitted at the challenge-unit for a period of about 10 days.
**Challenge phase (in-patient)**	The day after admission, each subject will be challenged with approximately 1500 cfu of the fully virulent *S. flexneri* 2a 2457T strain. The challenge dose will be administered orally approximately 28 days after the second vaccination dose.

	<u>Clinical Monitoring</u><br>After challenge, subjects will be monitored for about 9 days for shigellosis by daily medical checks, vital sign determinations, grading and weighing of all stools. A research monitor will be available to support the team. Monitoring for fecal shedding of the challenge *S. flexneri* 2a 2457T strain will occur daily after challenge by standard microbiological and molecular assays (detailed description given in separate SSP).<br>Mucosal and systemic antibody responses to the vaccine as well as to the *S. flexneri 2a* 2457T challenge strain will be assessed throughout the study, as shown in the schedule of events.<br><u>Antibiotic treatment</u><br>- Routine antibiotic treatment will commence for all subjects at about 120 hours post-challenge. All subjects will be treated with ciprofloxacin (500 mg orally twice daily for three days), or alternatively with trimethoprim 160 mg / sulfamethoxazole 800 mg, orally twice daily for three days, or amoxicillin (500 mg) orally three times daily for three days.<br>Early antibiotic treatment after challenge, may commence when any of the following criteria are identified and a study physician considers it to be warranted:<br>- When volunteers met the primary endpoint (clinical definition of shigellosis)<br>Or<br>- Oral temperature ≥ 39°C<br>Or<br>- Any other reason warranting the early treatment in the physician's opinion<br>If, because of illness, a subject is unable to take oral antibiotics, intravenous antibiotics may be given at an appropriate dose based on weight and clinical status.<br>All subjects will be discharged from the inpatient unit when they are well and have had at least two consecutive stool cultures negative for the challenge strain.<br>A follow-up call 6 months after challenge (about D240) will be performed to inquire about the occurrence of any new chronic health conditions, serious health events, or hospitalizations and to complete a functional bowel survey to assess for new onset chronic bowel problems.
**Study Procedures**	Starting 180 days before enrollment, subjects could be pre-screened using an IRB-approved screening protocol. During this visit, sera will be collected for the analysis of the *S. flexneri* 2a-IgG threshold. Only subjects who are *S. flexneri* 2a-naïve, as determined by absence of specific antibodies in the serum (2a-IgG titer <2500), will be selected for the study specific screening visit.<br>Subjects will be injected with vaccine or placebo at D0 and at D28 (± 4 days).<br>All subjects will have about 6 out-patients visits: Study-specific screening, D0, D7, D28, D35 and D84 or D56, depending if subjects will be challenged or not.<br>Persistency of the immune-response will be tested as a post-study follow-up about one year after vaccination.<br>Subjects will receive three follow-up phone calls on D1, D29 and approximately 6 months after challenge, or if not challenged after the last injection.<br>During the last follow-up call, a final safety review of adverse events (AEs) with a late onset will be performed. Investigators will ask about the general health status of the subject, as well as about significant symptoms the subjects experienced. In particular:

	- Recording of new diagnoses (e.g. reactive arthritis or IBS for the subjects challenged), hospitalizations or new chronic illnesses occurring after last study visit<br>- Recording of SAEs<br>An independent Adjudication Board will review attack rates and study results with regards to the efficacy endpoints.<br>A detailed description of the activities and sampling performed at each visits is given in the schedule of events.
**Primary Objective**	To provide an indication that the Flexyn2a vaccine reduces Shigellosis incidence induced by a wild type *S. flexneri* 2a 2457T challenge.<br><br>Shigellosis is clinically defined as:<br><br>- severe diarrhea<br>or<br>- moderate diarrhea with fever or with one or more moderate constitutional or enteric symptom<br>or<br>- dysentery
**Primary Endpoint**	To show after challenge a lower attack rate of shigellosis in the arm injected with candidate-vaccine compared with the arm administered with placebo
**Secondary and Exploratory Endpoints**	**Efficacy endpoints:** The secondary efficacy endpoints are chosen to support the primary endpoint in determining if 2a-EPA has any protective efficacy. This will be done by assessing:<br>Number of subjects with moderate to severe diarrhea<br>Number of subjects with more-severe diarrhea<br>Number of subjects with fever<br><br>**Safety endpoint:**<br>Safety and tolerability of vaccination with *S.flexneri* 2a-EPA will be assessed by comparison of adverse events (AEs) profiles, incidence rates and clinical laboratory data, between the active and placebo groups for one month following each vaccination (until last visit before challenge).<br>After challenge, causality of all the AEs collected until last study visit will be additionally assessed toward challenge.<br>All SAEs will be recorded any time after enrollment and during the whole study.<br><br>**Immunogenicity and microbiology endpoint:**<br>To further assess the systemic and mucosal immunogenicity of the vaccine the level of anti 2a-serum IgG and anti 2a-stools IgA will be compared between vaccine and placebo (secondary endpoint). In addition, as exploratory endpoint, it will be considered if any of the immune parameters measured predict protection and may serve, therefore, as potential immune correlate of protective immunity in future studies.<br>To compare the immune response induced by the vaccine with the immune response induced by wild type challenge in placebo group, level of 2a antibodies in vaccine and placebo will be compared<br>To compare intestinal colonization by the challenge strain among vaccinees and controls, quantitative stool cultures will be done on multiple time points after challenge (and number or colonies from vaccine and placebo groups be compared at both time points.<br>Blood and stools samples for immune assays will be collected according to schedule described in section 2.2.
**Definitions**	More severe diarrhea: ≥10 loose (grade 3-5) stools within 24h or ≥1000 gr loose (grade 3-5) stools within 24h

	Severe diarrhea: ≥6 loose (grade 3-5) stools within 24h or >800gr loose (grade 3-5) stools within 24h<br>Moderate diarrhea: 4 to 5 loose (grade 3-5) stools within 24h or 401-800gr loose (grade 3-5) stools within 24h<br>Mild Diarrhea: ≥2 loose (grade 3-5) stools weighing ≥200gr within 48h or 1 loose stool weighing ≥300gr, not meeting the definition for moderate or severe<br>Fever: measured oral temperature ≥ 38°C confirmed within about 20 min<br>Dysentery: at least two loose (grade 3-5) stools with gross blood within any 24 hours (confirmed by hemoccult) and reportable constitutional symptoms<br>Constitutional/Enteric Symptom: Nausea, Vomiting, Abdominal Cramps/Pain, Myalgia, Arthralgia, Rigors, Tenesmus and Fecal urgency
**Eligibility Criteria**	**Inclusion criteria:**<br>1. Male or female age 18-50 years (inclusive)<br>2. Good health, without clinically significant medical history or physical examination findings.<br>3. Negative serum pregnancy test at screening, and negative urine before each vaccination and before challenge for female subjects of childbearing potential.<br>4. Females of childbearing potential must agree to avoid pregnancy by use of effective contraception. Abstinence is not acceptable as effective contraception. Female subjects unable to bear children must have this documented (e.g. tubal ligation or hysterectomy).<br>5. Willingness to participate in the study after all aspects of the protocol have been explained and written informed consent obtained.<br>6. Completion of a training session and demonstrated comprehension of the protocol procedures, knowledge of Shigella-associated illness, and by passing score of 70% or better on a written examination (comprehension test).<br>7. Availability for the study duration, including all planned follow-up visits.<br>8. Willingness to refrain from participating in other studies of investigational products until completion of the last study visit.<br>**Exclusion criteria:**<br>1. Women currently nursing.<br>2. Presence of a significant medical or psychiatric condition which in the opinion of the investigator precludes participation in the study.<br>3. Clinically significant abnormalities in screening hematology or serum chemistry as determined by PI or PI in consultation with the research monitor and sponsor.<br>4. Presence in the serum of HIV antibody, HBs-Ag, or HCV antibody (if confirmed positive by HepC confirmatory test, i.e. RIBA, PCR)<br>5. Evidence of IgA deficiency (serum IgA < 7 mg/dl or limit of detection of assay).<br>6. Evidence of current excessive alcohol consumption or drug dependence.<br>7. Evidence of impaired immune function.<br>8. BMI <19 and ≥35<br>9. Recent vaccination or receipt of an investigational product (within 30 days before vaccination or until last study visit)<br>10. Personal history of an inflammatory arthritis.<br>11. Positive blood test for HLA-B27 antigen.<br>12. Personal history of irritable bowel syndrome as defined by Rome III criteria.<br>13. Treatment with immunoglobulins or blood products within 3 months from first candidate vaccine injection.<br>14. Regularly abnormal stool pattern (fewer than 3 per week or more than 3 per day) or loose or liquid stools<br>15. Regular use of laxatives, antacids, or other agents to lower stomach acidity.<br>16. Use of any medication known to affect the immune function (e.g., systemic steroids) within 30 days preceding the first vaccination or planned use during the entire study period.<br>17. Known allergy to any of the following antibiotics: ciprofloxacin, trimethoprim-sulfamethoxazole or penicillin.<br>18. Symptoms consistent with Traveler's Diarrhea concurrent with travel to

	countries where Shigella infection is endemic (most of the developing world) within two years prior to dosing, OR planned travel to endemic countries during the length of the study.<br>19. Vaccination for or ingestion of Shigella within 3 years prior to vaccination<br>20. Use of antibiotics during the 7 days before vaccination and challenge<br>21. Use of proton pump inhibitors, H2 blockers or antacids within 48 hours prior to challenge.<br>22. Serum IgG titer to *Shigella flexneri* 2a LPS ≥ 2500<br>23. Current occupation involving handling of Shigella bacteria<br>24. History of allergy to any vaccine or to soy<br>25. Any other criteria which, in the investigator's opinion, would compromise the ability of the subject to participate in the study, the safety of the study, or the results of the study
**Sample Size**	Up to 72 subjects (about 36 per group) will be randomized for the outpatient vaccination stage, and up to 60 (approximately 30 per group) will be admitted for the subsequent challenge stage. The null hypothesis for the study is that the incidence of shigellosis in the vaccine and placebo groups is the same or higher for the vaccine group; the alternative being that the incidence of shigellosis will be lower in the vaccine recipients. Assuming an attack rate for diarrhea in the placebo group estimated to be 70% and an attack rate of no higher than 30% in the vaccine group (equivalent to >57% protective efficacy), a total of 28 to 30 subjects per group will allow for at least 80% power to detect a significant difference (p<0.05; lower bound of 95% confidence interval around point estimate of efficacy of > zero) in attack rates between the vaccine and placebo groups.
**Statistical methods and analysis**	Descriptive statistics (n, mean, standard deviation, median and ranges for continuous variables, frequencies and percentages for categorical variables) will be provided by treatment group and, where applicable, visit. All subjects receiving placebo will be pooled to form the placebo treatment group. If a notable difference between the two groups is observed, an appropriate statistical test might be performed for exploratory purposes.

2.1 Trial design diagram

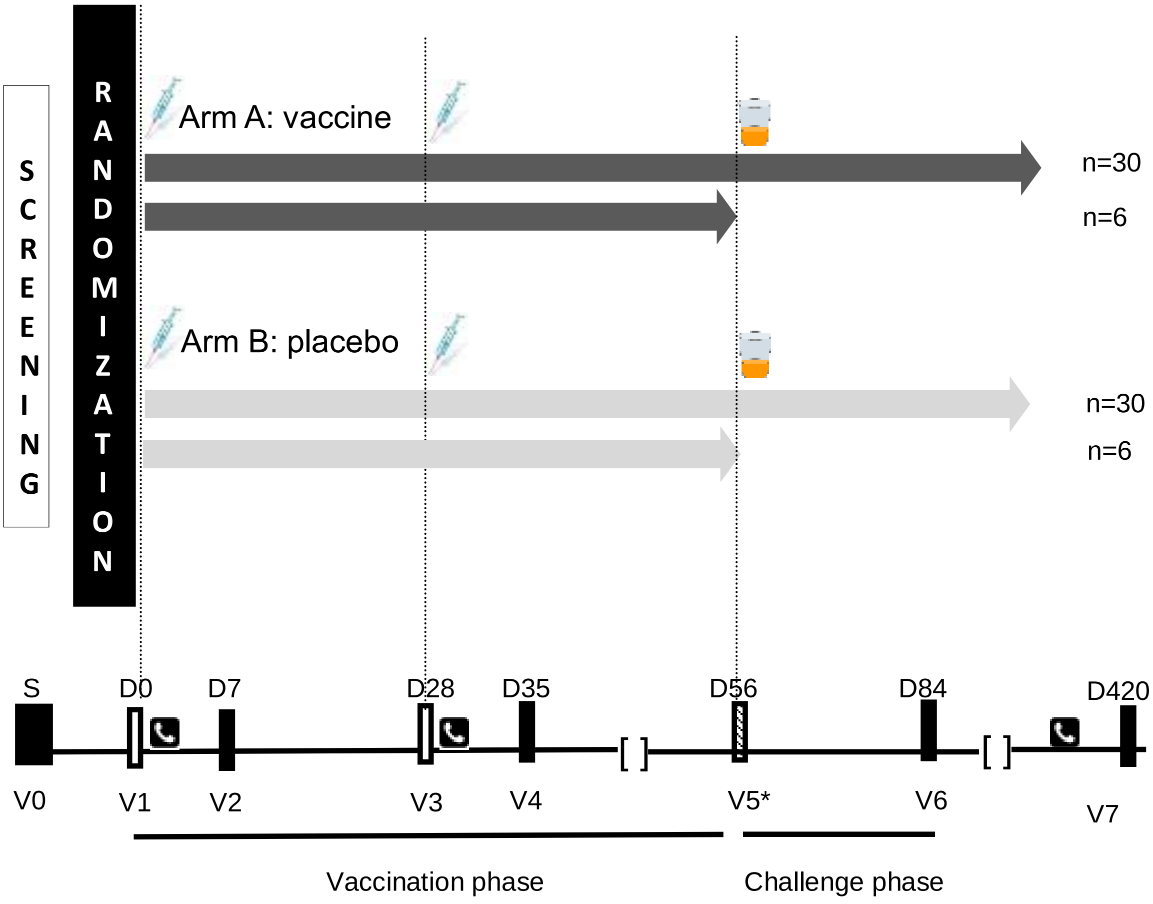

Phone calls at Day1 (P1), Day29 (P2) and Day240 (P3)

* Last study visit at Day 56 for subject vaccinated but not challenged

2.2 Trial schedule

	Screening		Outpatient Visit						Inpatient										Outpatient			
Study Event	Pre-screening	Study-screening	V1	P1	V2	V3	P2	V4	V5										V5§	V6	P3	V7
Study day	-180 to -2	-30 to -2	0	1	7	28	29	35	C-1 (55)	C0	C1	C2	C3	C4	C5	C6	C7	C8	56§	84	240	420
Compliance Ranges			--	--	±1d	±4d	--	±1d	±14d	--	--	--	--	--	--	--	--		±4d	±7d	(208§) ±14d	±80d
Informed Consent (screening) [A]	X	X																				
Comprehension Assessment [A]	X	X																				
Eligibility[B]			X			X			X	X												
2a-LPS IgG Titer (Shigella prior exposure test)	X																					
Medical History [C]	X	X																				
Physical Examination [D]	(X)	X	X			X			X	X	X	X	X	X	X	X	X	X	(X)	(X)		
Chemistry & CBC w/ diff [E]	*	X	X		X	X		X	X										X			X
Anti-HIV-1, Anti-HCV, HBsAg		X																				X
Total IgA, HLA B27		X																				
ABO blood type																						X
Urine tox screen[F]	*	X							(X)													

	Screening		Outpatient Visit						Inpatient										Outpatient			
Study Event	Pre-screening	Study-screening	V1	P1	V2	V3	P2	V4	V5										V5§	V6	P3	V7
Study day	-180 to -2	-30 to -2	0	1	7	28	29	35	C-1	C0	C1	C2	C3	C4	C5	C6	C7	C8	56§	84	240 (208§)	420
Pregnancy test [G]		X	X			X			X										X	X		X
Functional bowel survey		X																			X	
Study-drug injection			X			X																
Clinical Check Vitals (BP, HR,	X	X	X		X	X		X	X	X	X	X	X	X	X	X	X	X	X	X		X
AEs, con meds collection[I]			X	X	X	X	X	X	X	X	X	X	X	X	X	X	X	X	X	X	(X)	
Phone Call				X			X														X	
Memory aid distribution[J]			X			X																
Memory aid review[J]					X			X														
Admission to the inpatient unit									X													
Challenge										X												
Stool weighing, grading [L]										X	X	X	X	X	X	X	X	X				
Quantitative stool culture[Q]										(X)	(X)	(X)	(X)	(X)	(X)							
Qualitative stool culture									(X)	(X)	X	X	X	X	X	X	X	X				
Hemoccult [M]										X	X	X	X	X	X	X	X					

	Screening		Outpatient Visit						Inpatient											Outpatient		
Study Event	Pre-screening	Study-screening	V1	P1	V2	V3	P2	V4	V5									V5§		V6	P3	V7
Study day	-180 to -2	-30 to -2	0	1	7	28	29	35	C-1 (55)	C0	C1	C2	C3	C4	C5	C6	C7	C8	56 §	84	240 (208§)	420
Collection of stools for immunoassays (IgG, IgA, Cytokines)		X	X			X			X	X	X	X	X	X	X	X	X		X	X		(X)
Serology (IgG, IgA, Cytokines)[K]			X		X	X		X	X				X				X		X	X		X
Serology for immunoassays (i.e. BCA)[K]			X			X		X	X								X		X			X
Blood for PBMCs[K]			X		X	X		X	X				X				X		X			X
Memory B Cells[K]			X						X								X		X	X		X
T cells responses[K]			X		X	X		X	X				X				X		X			
Serum CRP[K]									X				X				X					
Start antibiotic therapy[N]															X							
Planned discharge[O]																		X				
Last study visit																			X	X		
End of study contact[P]																					X	
Approximate Blood Volume (ml)	5	35	112.5	0	72.5	82.5	0	82.5	115.5	0	0	0	68	0	0	0	115.5	0	112.5	35		450

[A] Pre-screening (-180 - -2) done under the JH200 screening consent; screening (-30 - -2) with study specific ICF. Comprehension assessment will be completed with each consent obtained. An ELISA to screen potential subjects for prior exposure to *S. flexneri* 2a will be performed on serum collected during this period and tested as specified in written SSPs

[B] After screening subject continuing eligibility must be confirmed by asking and reassessing relevant inclusion and exclusion criteria before each vaccination and before challenge on either day of admission or challenge day.

[C] Medical history will be obtained during screening and updated as needed.

[D] Complete Physical Examination will be completed during screening and focused Physical Exams will be performed as indicated. Physical Examination (PE) to include: HEENT (Head; Ears; Eyes; Nose; Throat), skin, respiratory (lung), cardiovascular (heart), abdomen, neurological and musculoskeletal system. Weight and height will be measured at screening only for determination of BMI.

[E] CBC w/diff and Chemistry: Serum Chemistry will include: Serum transaminases (ALT/AST), Na+, K+, CL-, HCO3-, glucose, Blood urea nitrogen (BUN) and creatinine. Samples to determine eligibility need to be obtained between - 30 and - 2. Follow up sample to be taken if clinically significant abnormalities are seen. Hematology and serum chemistry on admission day prior to challenge is done to evaluate safety, but NOT to establish ongoing eligibility before challenge (results do not have to be available before challenge). Clinically relevant laboratory abnormalities will be recorded as MH if obtained before 1st injection and as AE if obtained after 1st injection. Not clinically significant laboratory abnormalities can be recorded on the MH if deemed necessary by the PI.
A total IgA (Serum IgA < 7 mg/dL or limit of detection of assay) will be considered exclusionary.
If indicated, subjects may have additional blood draws taken for monitoring for other safety reasons.
*Samples may be completed during pre- screening but are not required.

[F] Urine toxicology, for the presence of amphetamine, barbiturates, opiates, phencyclidine, cocaine, benzodiazepine, methadone and propoxyphene screening at the discretion of the study clinician. May be done during pre-screening but not required. Tests must be completed -30 - -2. Urine will be collected and saved on admission to the inpatient unit for toxicology and potential antibiotic testing if indicated. Additionally urine toxicology not limited to above may be done at the PI's discretion.

[G] Serum pregnancy test will be completed during screening between -30 - -2 and prior to challenge. Urine pregnancy tests are to be performed prior to injections and on the last scheduled follow-up visit to the clinic. Pregnancy test pre challenge may be done on admission day or the day of challenge prior to challenge. If the serum pregnancy test results are not available by the time of challenge a urine pregnancy test will be done.

[H] Vital Signs (VS) will include heart rate, blood pressure, and oral temperature. Vital signs are obtained from study subjects at protocol-directed time points throughout the study. There will be instances when a vital sign needs to be repeated. Standard practice will be to repeat the vital sign within approximately 30 minutes of the original reading. Only the vital sign that needs to be repeated will be repeated. Both the original and repeat measurements will be recorded in the study source documents. If, in the judgment of the PI or his designee, the repeat measurement is a more appropriate reflection of the subject's vital sign, the repeat measurement will be recorded in the CRF field for that measurement. The non-repeated vital signs will be recorded in their respective eCRF with the time, the repeated vital sign will be entered even though the vital sign was taken at a little later time point, if it is felt that the repeat vital sign is the more accurate reflection.
Vital signs will be documented in the subject's source documents:

- Sometime during the screening visit
- On days of vaccination, prior to vaccination
- On follow up days during vaccination phase
- Sometime during the day of admission to challenge
- Before and after challenge
- At least 3 times daily during in-patient period
- At the day 84 visit

A grade 1 bradycardia, or other grade 1 abnormalities will not be considered to be exclusionary at screening or an AE for the study, unless judged to be clinically significant by the PI.

[I] AE collection will be initiated after first injection and throughout the study until the last scheduled follow-up visit at the clinical center. ConMed will be collected until last study visit (V6).

[J] A symptom memory aid will be used by subjects to record systemic and local injection site AEs, as well as any AEs and ConMed. Before discharge after each injection, the volunteer will be provided with supplies including a thermometer and a Memory Aid; instructed in their use; and provided with written materials, follow-up visit information, and ID card with contact telephone numbers for study staff. The memory aid will be reviewed during follow up visits with study staff. The memory aid entries will be used to complete pertinent source documents.

[L] Stool weighing and grading. During the in-patient period all stool samples are collected, weighed and graded. If a subject is discharged early, no further stool samples will be collected for culture, weighing or grading.

Stool sample or rectal swab will be collected for microbiology and molecular assays described in written SSPs. Qualitative culture from stools collected at C-1 and C0 will be performed only when a baseline culture is required.

[M] Stool for Hemoccult will be performed only when blood in the stool is suspected as per written SSP.

[K] Blood and stools for immunoassays will be collected and processed as specified in written SSPs.

[N] All subjects will be treated with 6 doses of ciprofloxacin (cipro) 500 mg BID, to start five days after challenge for three days, or suitable alternative trimethoprim-sulfamethoxazole (1 DS tablet twice daily for three days) or amoxicillin (500 mg three times daily for 3 days); unless subject meets criteria for early antibiotic treatment.

[O] Criteria for discharge from the unit: Subjects can be discharged from the inpatient unit when they have completed a minimum of 2 doses of Abx, clinical symptoms are resolved or resolving and 2 consecutive stool cultures are negative for Shigella. Subjects will be required to complete their Abx as outpatients. It is expected that most subjects will be discharged on days seven or eight. If a subject does not fulfill criteria for discharge he/she may be required to stay on the unit until all criteria have been fulfilled. If a subject meets eligibility criteria for early discharge they will not require continued inpatient monitoring and collection of specimens and may leave the unit

[P] At about 6 months after challenge (about Day 240) or 6 months after last vaccination (for subjects not challenged, Day 208), phone calls will take place to all subjects to inquire about the occurrence of any serious new health events, new chronic illnesses or hospitalizations. Subjects challenged will also answer questions regarding the occurrence of reactive arthritis symptoms and their gastrointestinal history over the past three months; the functional bowel survey will be completed.

[Q] If available, 1 stool per day per subject may be used for quantitative culture from challenge to the initiation of antibiotic therapy.

[§] applicable to subjects enrolled but not challenged

(X) denotes an optional assay or procedure at that given time-point

3.0 TABLE OF CONTENTS

1.0 TITLE PAGE 1
1.1 GENERAL ADMINISTRATIVE INFORMATION 2
1.2 SIGNATURE PAGES 5
2.0 SYNOPSIS 7
2.1 TRIAL DESIGN DIAGRAM 12
2.2 TRIAL SCHEDULE 13
3.0 TABLE OF CONTENTS 19
3.1 LIST OF TABLES 21
3.2 LIST OF APPENDICES 21
4.0 LIST OF ABBREVIATIONS 21
5.0 INTRODUCTION 23
5.1 BACKGROUND ON SHIGELLA EPIDEMIOLOGY 23
5.2 STUDY RATIONALE 24
5.3 SUMMARY OF NON-CLINICAL DATA 26
5.4 SUMMARY OF PHASE I CLINICAL DATA 27
5.5 CHALLENGE STRAIN 28
5.6 POTENTIAL BENEFITS AND RISKS 28
5.7 DOSE AND ADMINISTRATION RATIONALE 30
6.0 TRIAL OBJECTIVE AND ENDPOINTS 32
6.1 PRIMARY ENDPOINT 32
6.2 SECONDARY ENDPOINTS 32
6.3 EXPLORATORY OBJECTIVES 33
7.0 INVESTIGATIONAL PLAN TRIAL DESIGN 34
7.1 TRIAL DESIGN 34
7.3 TRIAL POPULATION 35
7.4 TRIAL SCHEDULE 38
8.0 STUDY DRUG ADMINISTRATION 38
8.1 FLEXYN2A CANDIDATE VACCINE 38
8.2 STUDY DRUG FORMULATIONS AND SUPPLY 38
8.3 STUDY DRUG PREPARATION 39
8.4 STUDY DRUG ADMINISTRATION 39
8.5 STUDY DRUG STORAGE 39
8.6 REPLACEMENT OF UNUSABLE VIALS 39
8.7 LABELING AND PACKAGING 40
9.0 TRIAL CONDUCT 40
9.1 TRIAL VISITS 40
9.2 TRIAL PROCEDURES 50

9.3 STUDY DISCONTINUATION 54

10.0 SAFETY ASSESSMENTS 57

10.1 DEFINITION OF TERMS 58
10.2 EVALUATION OF AE AND SAE 59
10.3 ASSESSMENT OF INTENSITY 61
10.4 ASSESSMENT OF CAUSALITY 62
10.5 FOLLOW UP OF AEs AND SAEs AND ASSESSMENT OF OUTCOME 64
10.6 AEs REPORTING REQUIREMENTS 64
10.7 SAEs REPORTING REQUIREMENTS 65

11.0 DATA ANALYSIS 67

11.1 ANALYSIS POPULATIONS 67
11.2 STATISTICAL ANALYSIS 68
11.3 EFFICACY ANALYSIS 68
11.4 SAFETY ANALYSIS 69
11.5 IMMUNOGENICITY ANALYSIS 70
11.6 PROTOCOL DEVIATIONS 71
11.7 SAMPLE SIZE CALCULATION 71
11.8 ACCOUNTING FOR MISSING, UNUSED, AND SPURIOUS DATA 71
11.9 ADJUDICATION BOARD 72

12.0 ADHERENCE TO ETHICAL, REGULATORY, AND ADMINISTRATIVE CONSIDERATIONS 73

12.1 INDEPENDENT ETHICS COMMITTEE OR INSTITUTIONAL REVIEW BOARD 73
12.2 SUBJECT INFORMATION AND CONSENT 73
12.3 SUBJECT ID CONTACT CARD 74
12.4 COMPENSATION FOR PARTICIPATION 74
12.5 REGISTRATION OF THE TRIAL 75
12.6 DATA COLLECTION FORMS 75
12.7 ELECTRONIC CASE REPORT FORMS FOR RANDOMIZED SUBJECTS 75
12.8 DATA COLLECTION FOR SCREENING FAILURE SUBJECTS 76

13.0 ADMINISTRATIVE MATTERS 76

13.1 SPONSOR 76
13.2 INVESTIGATOR 76
13.3 CLINICAL STUDY REPORT 77
13.4 CLINICAL RESEARCH ORGANIZATION 77
13.5 MONITORING 77
13.6 QUALITY ASSURANCE AUDIT 78
13.7 ACCESS TO ORIGINAL STUDY DATA 78
13.8 DATA COLLECTION AND RECORD RETENTION 78
13.9 INSURANCE FOR CANDIDATE VACCINE-INDUCED INJURY 78
13.10 EARLY TRIAL TERMINATION 79
13.11 PUBLICATIONS AND OTHER RIGHTS 80
13.12 TRIAL DOCUMENTS AND RECORDS RETENTION 80

14.0 REFERENCES 81

3.1 List of tables

Table 1 Injection formulation for each treatment arm

Table 2 Solicited Adverse Events

Table 3 Classification of intensity of local AEs

Table 4 Assessment of severity of general AEs

3.2 List of appendices

Appendix A Laboratory Values Guidance

4.0 LIST OF ABBREVIATIONS

AE(s)	Adverse Event(s)
ALT	Alanine Aminotransferase
AST	Aspartate Aminotransferase
BUN	Blood Urea Nitrogen
CBC	Complete blood count
CIR	Center for Immunization Research
CRO	Contract Research Organization
DALY	Disability Adjusted Life Year
EC-4V	*Escherichia coli* 4-valent bioconjugate vaccine
eCRF	Electronic Case Report Form
ELISA	Enzyme-linked Immunosorbent Assay
EPA	recombinantly expressed *Pseudomonas aeruginosa* ExoProtein A
ER	Emergency Room
FDA	Food and Drug Administration
GCP	Good Clinical Practice
GEMS	Global Enteric Multicenter Study
HIV	Human immunodeficiency Virus
HBsAG	Hepatitis B Virus surface antigen
HCV	Hepatitis C Virus
HLA-B27	Human Leucocyte Antigen B27
HPLC	High-Performance Liquid Chromatography
IB	Investigator’s Brochure
IBS	Irritable Bowel Syndrome

ICF	Informed Consent Form
ICH	International Conference on Harmonization
IDES	Internet Data Entry System
Ig A/G/M	Immunoglobulin A/G/M
IRB/IEC	Institutional Review Board / Independent Ethics Committee
LPS	Lipopolysaccharide
MH	Medical History
MSD	Moderate-to-Severe Diarrhea
NMR	Nuclear Magnetic Resonance
NMRC	Naval Medical Research Center
MS	Mass Spectrometry
PBMC	Peripheral Blood Mononuclear Cell
PCR	Polymerase Chain Reaction
PE	Physical Examination
PI	Principal Investigator
q-PCR	quantitative-Polymerase Chain Reaction
RA	Reactive Arthritis
SAE(s)	Serious Adverse Event(s)
SAP	Statistical Analysis Plan
SBA	Serum Bactericidal Assay
SOP	Standard Operating Procedure
SSP	Study Specific Procedure
SUSAR	Suspected Unexpected Serious Adverse Reaction
ULN	Upper limit of normal
WBC	Whole Blood Cells
WNL	Within Normal Limits
WRAIR	Walter Reed Army Institute of Research
YLD	Years Lived with Disability

5.0 INTRODUCTION

5.1 Background on Shigella epidemiology

Shigellosis, also known as bacillary dysentery, is a serious and relatively common disease in tropical endemic areas that is caused by infection with *Shigella* bacteria. In addition to causing watery diarrhea, *Shigella* is a major cause of dysentery, which is characterized by fever, cramps, and blood and/or mucus in the stool. Humans and non-human primates are the only natural hosts for these bacterial microorganisms, with children below the age of 5 years and elderly representing the population groups particularly vulnerable to suffer more severe consequences of such a disease. It is estimated that shigellosis causes worldwide at least 80 million cases of bloody diarrhea and 700,000 deaths each year. Ninety-nine percent of infections caused by *Shigella* occur in developing countries, and the majority of cases (~70%), and of deaths (~60%), occur among children less than five years of age (31).

In addition to mortality estimates, in the Institute for Health Metrics and Evaluation recent global burden of disease study, shigellosis associated Disability-Adjusted Life Years (DALYS) were estimated at 7 million (~8% of all diarrhea DALYS) and Years Lived with Disability (YLDs) were estimated at 744,000 (~9% of all diarrhea YLDs) (18, 18, 18, 29). Travelers and deploying military service members also frequently suffer from shigellosis and these cases and their sequelae also contribute to the overall global burden of disease.

Recent studies in sub-Saharan Africa and South Asia conducted under the Global Enteric Multicenter Study (GEMS) have reaffirmed the continuing importance of Shigella as a major cause of moderate-to-severe diarrhea (MSD) among children less than five years of age, with Shigella among the top four causes of potentially life-threatening diarrheal illness in both regions. GEMS data also indicate that MSD among children living in these low-resource areas remains associated with a significantly increased risk of mortality, cognitive defects, and stunting.

Due to the technical difficulties and lack of sensitivity associated with the direct culture methods used to identify Shigella, disease incidence and morbidity figures for this pathogen are likely to be underestimated. Recent polymerase chain reaction (PCR)-based studies indicate that Shigella may be responsible for many of the diarrhea cases that typically remain undiagnosed when traditional culture methods are used. Similarly, in a recent evaluation of quantitative-PCR (q-PCR) as a potentially more sensitive method for the diagnosis of shigellosis, using samples from GEMS, investigators found that use of q-PCR almost doubled the number of MSD cases attributable to Shigella (from 9.6 percent by traditional culture methods to 17.6 percent by q-PCR) (15).

Since a very low inoculum (10 microorganisms) is sufficient to cause disease, *Shigellae* disseminate easily in settings where there is a high population density and insufficient medical care. Overcrowded situations combined with reduced personal hygiene and inadequate sanitation, as is typically the case for people living in the poorest areas of low and middle income countries, as well as travellers or armed forces deployed to less industrialized countries, represents an ideal setting for *Shigella* outbreaks (8).

Shigella is an antigenically polymorphic pathogen whose official taxonomy encompasses the following four groups: *S. dysenteriae* (group A with 15 serotypes), *S. flexneri* (group B with 15 serotypes), *S. boydii* (group C with 20 serotypes) and *S.*

sonnei (group D with a single serotype). While *S. boydii* is uncommonly associated with diarrheal diseases, all other groups have serotypes considered major agents of endemic or epidemic shigellosis around the world. *S. flexneri* serotypes have been associated with endemic shigellosis among children in developing countries, such as China, South and Southeast Asia, Egypt, Kenya, Peru, and Israel, where up to 90% of cases are attributable to this *Shigella* group (1, 4, 13). *S. sonnei* is the predominant serotype that causes shigellosis in industrialized countries, including the United States, and it is also an important agent of travelers' diarrhea. The *S. dysenteriae* type 1 is uncommonly endemic, but can cause disease with severe complications and is historically associated with devastating pandemics with high case-fatality rates in all age groups, as was the case in outbreaks in Central America, Central Africa and Southeast Asia (5),(22), (27).

In most cases, infections with human enteropathogenic bacteria resolve after treatment with antibiotics. However, the same pathogen can cause a different outcome in developing countries, where there are inadequate hygiene settings and limited access to standard of care. Moreover, even if antibiotics are globally the standard of care for bacterial diseases, the increasing widespread prevalence of resistant strains, particularly among Shigella strains, is reducing the effectiveness of this therapeutic option. As therapeutic options narrow, the need for safe and effective vaccines becomes a priority.

In order to deliver the broadest possible protection against the most common serotypes, a *Shigella* vaccine should be at least tetravalent and contain antigens from *S. sonnei*, *S. flexneri 2a*, *3a* and *6*, so as to cover approximately 85% of currently circulating strains (14). The addition of the *S. dysenteriae* type 1 would be an asset for this multivalent vaccine, as it would also be useful to prevent future pandemic outbreaks.

5.2 Study rationale

Several attempts have been made so far toward the development of a functional vaccine against shigellosis and several strategies for vaccine production have been exploited.

Live-attenuated whole cell vaccines were shown to induce a robust immune response (24), but this approach carries a high risk of reactogenicity and reversion. The safer approach of using inactivated whole-cell vaccines has been shown to be protective in animal models (19) and to be safe and immunogenic, when administered orally in volunteers at repeated high doses (17). However, the need for a high oral inoculum represents a continuous challenge for the further development of this category of vaccines (28).

Different strategies have taken advantage of the lipopolysaccharides (LPS) present on the surface of *Shigella* bacteria, which has been shown to elicit a serotype-specific immune response (9), (26) detected in association with convalescence from shigellosis.

Vaccine formulations where the LPS was complexed to protein components to form proteasomes or simply added to conserved bacterial proteins to form macromolecular complexes, have been shown to elicit a LPS-mediated serotype-specific protection in animals and to be safe when administered intra-nasally in healthy volunteers (10), (25).

Alternatively, conjugate vaccines, where the serotype-specific O-antigen component of the LPS was chemically linked *in vitro* to a detoxified recombinantly expressed *Pseudomonas aeruginosa exoprotein* A (EPA), have been tested in clinical trials. The most advanced conjugate vaccines include *S. flexneri 2a* and *S. sonnei* EPA conjugates

(6, 20). These conjugates have been shown to be safe and immunogenic in adults and young Israeli children, as well as in Israeli soldiers in field trials (6, 20) and lead to a significant level of seroconversion for 2 years (21). In addition, they have shown encouraging protective efficacy in limited field trials in Israel (2, 7, 21).
However, current strategies for the development and production of a multivalent Shigella vaccine are complex and expensive, hindering the further development of cost-effective vaccines.
The Flexyn2a candidate vaccine is a further step in the development of a safe and immunogenic vaccine, as it combines the advantages of the strong immune-response that conjugate vaccines induce with the homogeneous and consistent manufacturing process offered by the bioconjugation technology (for details see Investigator Brochure Flexyn2a). This technology allows the production of specific bioconjugates which can be used as novel vaccines (Fig.1). While polysaccharides themselves are poorly immunogenic and do not provide a long-lasting memory, conjugate vaccines are characterized by the induction of a T-cell dependent immune response, and thus lead to protective immunity, even in infants (3, 30)(16).

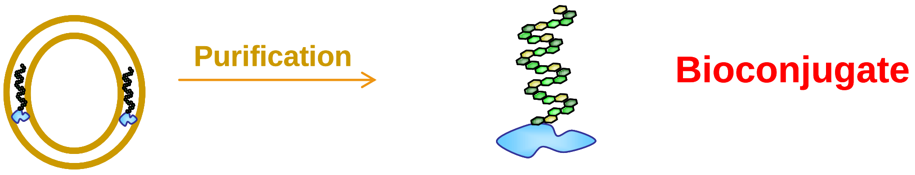

Figure 1*: Production of Bioconjugate in E. coli cells. Diverse polysaccharides can be conjugated to specific residues of any protein carrier using an enzymatic process. The Bioconjugate is extracted from the periplasm and purified by column chromatography to high purity. This novel production process is highly reproducible. The enzymatic conjugation simplifies the production process, improves the yie*ld significantly and enables the conjugation of antigens that c*annot easily be produced by chemical methods.*

Likely due to the difficulties of the chemical conjugation technologies (in regards to reproducibility and consistency of the products) or because of the safety concerns raised by attenuated vaccines, despite the numerous candidate Shigella vaccines so far tested in early clinical phases, only a limited number of product have been brought to efficacy studies. Field trials, done in Israeli recruits with a *S. sonnei* conjugate and in 1-4 year-old children with a *S. sonnei* and *S. flexneri* conjugate (7, 21), have provided indication of protective efficacy which was age-dependent and correlated to the magnitude of the vaccine-induced immune-response. Alternatively, several hybrids and attenuated vaccine candidates have been tested for efficacy in challenge studies with *S. flexneri*, where experimentally induced higher attack rates allows for testing efficacy on a smaller sample size. However, because of their limited clinical acceptability profile (symptoms of Shigella illness observed after vaccination) or low efficacy, the development of those candidate vaccines has not yet reached market authorization.

The good safety and immunogenicity profile shown in phase I with the Flexyn2a candidate vaccine justifies testing its efficacy against experimentally induced Shigella infection.

This phase 2b vaccine-challenge study is an essential step in obtaining evidence that the conjugate vaccine is able to induce a protective immune response before going into large complex field studies in children. In addition, it will support the development of a

multivalent vaccine against the most important *Shigella* serotypes so as to prevent a disease that represents an unmet medical need in developing countries and among travelers to endemic regions.

5.3 Summary of non-clinical data

The Flexyn2a candidate vaccine has been tested in laboratory tests, safety pharmacology and toxicology programs in order to generate data to allow estimation of risk prior to administration in humans. For any additional information on non-clinical tests, refer to the Investigator Brochure of Flexyn2a.

5.3.1 Quality data

QC testing and QA release of intermediate products, purified bulk substance and final product have been performed according to the International Conference on Harmonization (ICH) quality guidelines Topic Q6B: specifications, test procedures & acceptance criteria for biotechnological/biological products.

Specifications have been written & testing performed on the following:

- Master/working seed,
- Fermentation product,
- Purified Bulk Drug Substance (Active Pharmaceutical Ingredients),
- Product which includes the candidate vaccine with and without adjuvant

Stability studies on the drug substance as well as drug product have been conducted to determine the shelf-life of the product.

5.3.2 Non clinical pharmacodynamics

Based on data from clinical studies, it has been observed that the protection in adults may be related to antibody level response (32). Therefore, a defined range of animal tests has been performed to evaluate the antigenic property of Flexyn2a. Different animal species, with and without adjuvant, as well as different antigen doses have been tested. Details on the animal tests can be found in the Flexyn2a Investigator Brochure. In addition, toxicity and local tolerance of Flexyn2a vaccine formulated without and with adjuvant aluminum hydroxide following three intramuscular administrations in Sprague Dawley rats has been previously assessed and results are included into the Investigator Brochure.

5.4 Summary of phase I clinical data

The phase I study conducted with Flexyn2a has shown that the product is well tolerated and immunogenic.
Flexyn2a has been administered in a dose of 10 µg of polysaccharide with and without the adjuvant Alhydrogel, and two intramuscular injections. More details about the safety and immunogenicity data of the vaccine can be found in the Flexyn2a Investigator Brochure.
Briefly, Flexyn2a was able to induce a robust and statistically significant increase of *S. flexneri* 2a-specific serum IgG and IgA within subjects vaccinated with Flexyn2a either alone or adjuvanted with Alhydrogel, whereas no effect was observed in the placebo group.
Flexyn2a alone was able to induce at least a 4-fold increase (compared to pre-vaccination level) in the titer of *S. flexneri* 2a-serum IgG in 91.7% subjects (11 out of 12 subjects) and at least an 8-fold increase in 66.7% (8 out of 12 subjects) after the first injection. No change was observed after the second injection. A similar trend was observed for the serum IgA *S. flexneri* 2a-titers, even though the second injection reached a higher proportion of subjects (83.3%) that achieved at least an 8-fold increase in antibody level.
A between-group comparison showed that the addition of the adjuvant did not result in a statistically significant increase of the *S. flexneri* 2a-antibody levels, with average values that remained below the corresponding value of the unadjuvanted Flexyn2a group, regardless of antibody serotypes analyzed or number of injections.
A positive effect of adjuvant on the immunogenicity of the vaccine could not be seen neither by the analysis of the *S. flexneri* 2a-specific IgG and IgA produced by circulating peripheral blood mononuclear cells (PBMCs) isolated shortly after injection.

Flexyn2a was well tolerated and did not generate any safety concerns. The incidence and severity of the observed local and general adverse events were comparable to the safety profiles of licensed conjugate vaccines. Among the different study arms, a slightly higher incidence of local adverse events (e.g., pain and tenderness) was observed in the group receiving the adjuvanted Flexyn2a candidate vaccine. Compared to Flexyn2a alone, the overall incidence of any adverse events was slightly higher in the group of subjects receiving adjuvanted Flexyn2a (83.3%, vs. 75.0%), but all subjects in the placebo group experienced at least one adverse event (100%). In conclusion, the administration of repeated doses of Flexyn2a, alone or in combination with the adjuvant aluminium hydroxide,.did not raise any safety concern and showed a very good tolerability.

Although the phase I trial with Flexyn2a was the first-in-man trial with this bioconjugate, it was not the first time that an EPA-conjugated vaccine synthesized *in vivo* has been administrated to humans. In 2010, GlycoVaxyn successfully completed a similar phase I trial, where a *Shigella* dysenteriae bioconjugate candidate vaccine, consisting of the O1 polysaccharide antigen of *Shigella dysenteriae* conjugated *in vivo* to EPA (GVXN SD133), was administered to humans (12). In addition, in 2014-2015 GlycoVaxyn conducted a phase I study with a 4-valent conjugate vaccine (EC-4V) made of uropathogenic *E.coli* O-polysaccharides conjugated to EPA, in which more than 90 women were administrated the candidate vaccine (clinical study report in preparation). Here, as for the *S. dysenteriae* conjugate, not only was the technology used to link the polysaccharide antigens to the carrier protein comparable to the process used to

manufacture Flexyn2a, but also the same protein carrier (EPA) was used. Both the SD133 and the EC-4V studies have shown a good safety profile and immunogenicity of the respective candidate vaccine.

In conclusion, in line with the results obtained with the first tested O1-EPA and EC-4V bioconjugates, also Flexyn2a has demonstrated to be safe and able to elicit a robust immune response. The data obtained qualify Flexyn2a as a valuable candidate vaccine whose ability to confer protective immunity against *Shigella flexneri* 2a will be evaluated in this phase 2b challenge study.

In 2015, GlycoVaxyn AG was acquired by GlaxoSmithKline and all the activities related to the clinical development of Flexyn2a have been shifted under the sponsorship of LimmaTech Biologics AG.

5.5 Challenge Strain

The 2457T *S. flexneri* 2a strain that will be utilized in this study as a challenge strain is an essential component of this Phase 2b study and is a well-characterized Shigella strain manufactured under current Good Manufacturing Practice condition at the WRAIR Pilot BioProduction Facility in Silver Spring, Maryland. This strain was originally isolated from a clinically ill patient in Japan in the early 1950's and has most frequently been given to volunteers participating in IND studies evaluating treatment or preventive intervention for the control and clinical management of Shigellosis (23, 23). The 2457 strain is also covered by its own manufacturing master file registered with the FDA (BB-MF-3408). In all, more than 400 people have been challenged with the strain in the published literature, and of the Shigella strains used in challenge studies, this is the least likely to cause dysentery. The 2457T strain has been used in 14 published trials at doses ranging from 100 to 10^8 c.f.u. (23) and additional challenge studies carried out by the CIR in recent years that have yet to be published. In total the CIR has done 4 inpatient challenge studies with the 2457T strain since 2001, with the most recent being in 2008. Diarrhea attack rates are somewhat dose-related under 1400 c.f.u, but plateau at higher doses to approximately 60 to 80% (23). This strain is susceptible to the antibiotics that will be used for treatment (ciprofloxacin, trimethoprim-sulfamethoxazole, amoxicillin).

5.6 Potential benefits and risks

This study will not be associated with any potential benefit to study subjects. It is hoped that information gained in this study will contribute to the development of a safe and effective vaccine against Shigella for use in at-risk travelers, infants and young children in developing countries.

Risks to the subjects are associated with vaccination, challenge, venipuncture, and isolation. The risks from study-related procedures are outlined below. Female subjects will be cautioned of the unknown risk of study vaccines to the fetus and must agree to use effective birth control methods for the duration of the study.

<u>Risks associated with vaccination:</u>
The risks that may be anticipated in this clinical study are considered to be consistent with those associated with conjugate vaccines already available on the market; local

reactions at the injection site, such as pain, redness, induration and swelling; transient general symptoms, such as fatigue, headache, diarrhea, fever, muscle pain, arthralgia, malaise, erythema, nausea or vomiting, abdominal pain, and chills.

The following events were observed in the previous phase I study with the Flexyn2a candidate vaccine:

- Pain and swelling at the injection site (local reaction)
- General symptoms like, headache, muscle and joint aches, and diarrhea

Based on clinical experience with other conjugate vaccines and with the results of the phase I with Flexyn2a, no SAEs judged to be likely related to the bioconjugate are expected.

<u>Risks associated with challenge:</u>
Illness caused by Shigella organisms ranges from mild-to-severe watery diarrhea that may contain mucus or blood, with nausea, vomiting, abdominal cramps/pain, headache, excessive flatulence, anorexia, fever, generalized muscle and/or joint aches, malaise, fatigue, fecal urgency, rigors or tenesmus. For most adults the illness is not life threatening but often leads to mild to moderate dehydration, transient protein malabsorption and significant inconvenience associated with loss of sleep and activity. Bacteremia is rare.
Potential risks associated with Shigella infection/challenge are not limited to the acute effects of disease, but might extend to post-infectious conditions, like reactive arthritis (characterized by symptoms including inflamed joints and inflammation of the genital, urinary, or gastrointestinal systems) or irritable bowel syndrome (IBS) (23). In order to limit the risk of post-infection conditions, history of autoimmune disease or inflammatory arthritis and positive test for HLA-B27 (marker associated with reactive arthritis) are checked at screening as exclusion criteria. In addition, a functional bowel disorder survey will be conducted at screening and at Day 240. Subjects with reported prior history of abnormal bowel patterns who might be at higher risk of this post infectious sequela will be excluded.

Study facilities will have experienced personnel and resources capable of effectively managing diarrheal illness and potential complications. Fluids, both oral and intravenous will be used to prevent and/or treat dehydration. All subjects will receive treatment with antibiotics; subjects with severe symptoms will be treated with antibiotics early based on pre-established criteria and the discretion of the clinician. Subjects will be discharged from the inpatient unit only when they are no longer shedding the challenge bacteria.

Side effects to the antibiotics (ciprofloxacin, trimethoprim-sulfamethoxazole or amoxicillin) used to treat the *Shigella* infection are possible. Antibiotics for use in this study are licensed approved medications that have been used extensively and shown to be very safe with only rare side effects. The Shigella challenge strain being used in this trial is highly sensitive to these antibiotics and it has been used to effectively treat subjects in several previous Shigella challenge studies. The most commonly reported side effects are gastrointestinal symptoms (i.e. nausea, vomiting, and diarrhea), skin rash, restlessness, or yeast infection (in women). Ciprofloxacin is not recommended for

use in pregnancy. Pregnancy is exclusionary for study participation and is documented through testing prior to receipt of investigational products.

Hand-washing and sanitary disposal of feces (including pretreatment with bleach) are the main elements of personal hygiene and will minimize the spread by person-to person infection; hand-washing will be emphasized to the volunteers and volunteers will be instructed not to share food or beverages. Volunteers and staff will be trained in proper techniques of hand-washing. Volunteers will be instructed as to the importance of completing the 3-day course of antibiotics.

Risk of secondary transmission is highly unlikely due to antibiotic treatment and because two confirmed negative stool samples are required prior to discharge.

Risks associated with Venipuncture
Risks occasionally associated with venipuncture include pain and bruising at the site of venipuncture, bleeding, infection, light-headedness, and syncope (rarely). Good venipuncture practices are performed during blood draws, which minimizes the risk to the volunteer.
Risks associated with Confinement
Use of the live bacterial challenge carries with it a potential risk to the community of transmission from study subjects to others. To mitigate this risk, subjects will be confined to an isolation facility during the time that they may be infectious to others. Subjects must agree to stay in the Isolation Unit in order to participate in the study. Confinement to the facility may be stressful as well as boring. Subjects will be screened by the study staff for compatibility with the requirements of the study and given time to acclimatize to the study environment before challenge is administered. Television, internet connections, and a variety of entertainment options will be available to study subjects during their inpatient stay.

In addition, risks associated with study participation are mitigated by the following:

- The study will be conducted at a well-equipped clinical site with clinical staff very experienced in the conduct of phase 2b trials with vaccines and with challenge.
- Clinical staff is trained on the bioconjugate product and is very experienced in the detection and accurate reporting of adverse events in a timely manner.

5.7 Dose and administration rationale

Historically the rationale used to define the best dosage of the licensed conjugate vaccines was to use the highest dose of polysaccharide permitted based on safety considerations pertaining to the carrier protein as a potential limiting factor. There is evidence that recovery from shigellosis correlates well with the serum level of pathogen-specific LPS antibodies (28). Therefore, the dose of polysaccharide is relevant, since the sugar moiety of the conjugate vaccine is the target of the protective immune response against Shigella. In this trial, 10 µg of polysaccharide will be administered, as this dose and regimen has been shown in the phase I to be safe and able to elicit a specific immune response. This dose does correspond to approximately 50 µg EPA, a very well characterized amount of carrier protein without safety concerns identified in previous clinical studies.

Indeed, *Shigella* O-antigens chemically conjugated to EPA have already been tested in clinical trials at doses of 25 µg of polysaccharide and an EPA burden of 50-75 µg. This dosage induced a significant immune-response and led to some protection in adults and young children with no safety concerns reported even in infants (6, 21).
Moreover, a dose of 10 µg of the *Shigella dysenteriae* O1-EPA bioconjugate vaccine, which contained 50 µg EPA as carrier protein, has been already tested in a phase I study (see section 5.4) and was found to be well-tolerated and to induce an adequate immune response.

At this stage of development, a dose-finding study is not planned, since the Flexyn2a bioconjugate is only one component of the final product in the development plan of a *Shigella* multivalent vaccine. Both O1-EPA and Flexyn2a are the first two components of a pentavalent vaccine that aims to protect against the *Shigella* serotypes that are most frequently involved in shigellosis worldwide. Once the clinical proof of concept is obtained and initial protection data are available from this phase 2b study, the final multivalent product will enter clinical development investigating dose-response.

The choice of an administration route is mainly based on previous experience with conjugate vaccines. Conjugate vaccines have been generally administered intramuscularly instead of subcutaneously to avoid marked local irritation, induration and skin discoloration at the injection site. For most intramuscular vaccine administrations the deltoid muscle of the upper arm is the preferred injection site (11). In the phase I conducted with Flexyn2a, the intramuscular administration route was chosen and the same route will be used for the phase 2b. The appropriate route of vaccine administration is critical for its effectiveness. Due to the nature of the candidate vaccines so far tested in challenge studies (hybrids or inactivated vaccines), the oral or the nasal route have been chosen. This study will be the first Shigella challenge study performed after administration of a parenteral vaccination. Previous indications that high level of Shigella-LPS serum antibodies may protect against shigellosis (28) and the positive result of field trials with conjugate vaccines further support the intramuscular administration route. In addition, the activation of specific, vaccine-driven mucosal immunity will be evaluated during this trial.

Although administration of one dose of Flexyn 2a has already shown to induce antibody response and no statistically significant difference has been observed with the level reached after the second injection, an effect of the second injection, helping to reach a more effective and prolonged immune response, could not be excluded due to the limited number of subjects tested in the phase I study. Moreover, based on the data obtained in the phase 1 study, the addition of the adjuvant did not show a beneficial effect on the induced 2a-specific antibody levels, but rather a higher incidence of AEs. Therefore, in the phase 2b study, two doses of the not-adjuvanted Flexyn2a bioconjugate candidate vaccine at an interval of approximately 1 month by intramuscular route will be evaluated.

6.0 TRIAL OBJECTIVE AND ENDPOINTS

To provide an indication that the Flexyn2a vaccine reduces Shigellosis incidence induced by a wild type *S. flexneri* 2a 2457T challenge.
Shigellosis is clinically defined as:

- severe diarrhea

or

- moderate diarrhea with fever or with one or more moderate constitutional or enteric symptom

or

- dysentery

Definitions
Severe diarrhea: ≥6 loose (grade 3-5) stools within 24h or >800gr loose (grade 3-5) stools within 24h

Moderate diarrhea: 4 to 5 loose (grade 3-5) stools within 24h or 401-800gr loose (grade 3-5) stools within 24h
Mild Diarrhea: ≥2 loose (grade 3-5) stools weighing ≥200gr within 48h or 1 loose stool weighing ≥300gr, not meeting the definition for moderate or severe

Fever: measured oral temperature ≥ 38°C confirmed within about 20 min

Dysentery: at least two loose (grade 3-5) stools with gross blood within 24 hours (confirmed by hemoccult) and reportable constitutional symptoms

Constitutional/Enteric Symptom: Nausea, Vomiting, Abdominal Cramps/Pain, Myalgia, Arthralgia, Rigors, Tenesmus and Fecal urgency (for definition of intensity see table 4, section 10.3).

6.1 Primary Endpoint

To show after challenge a lower attack rate of shigellosis in the arm injected with candidate-vaccine compared with the arm administered with placebo

6.2 Secondary Endpoints

6.2.1 Efficacy endpoints

The secondary efficacy endpoints are chosen to support the primary endpoint in determining if Flexyn2a has any protective efficacy. A complete list of all secondary endpoints will be provided in the statistical analysis plan. Following endpoints will be included:

Number of subjects with moderate to severe diarrhea
Number of subjects with more-severe diarrhea (defined as ≥10 loose (grade 3-5) stools within 24h or ≥1000 gr loose (grade 3-5) stools within 24h)
Number of subjects with fever

Additional endpoints are listed in section 11.3

6.2.2 Safety objectives

Safety and tolerability of vaccination with Flexyn2a will be assessed by comparison in incidence, severity, relationship and duration of solicited and unsolicited AEs and SAEs after injection of the candidate vaccine versus placebo during the vaccination period (from V1 to last visit pre-challenge). Furthermore, safety endpoints will be changes in haematological and biochemical parameters, comparing values before and after each vaccination (D0 and D28 vs. D7 and D35, respectively) and at the end of the vaccination period.

After challenge, causality of all the AEs collected until last study visit will be additionally assessed toward challenge.

All SAEs will be recorded any time after enrollment and during the whole study.

6.2.3 Immunogenicity objectives

Immunogenicity of the Flexyn2a candidate vaccine at the proposed dosage has been already shown in the phase 1 trial. During this phase 2b, the immunogenicity of the candidate vaccine will be further confirmed, by comparison of the 2a antigen specific serum-IgG and stool-IgA level between baseline (D0) and after each injection (D28 and D56) in the vaccine and placebo group.

6.3 Exploratory objectives

In this phase 2b, additional investigations might be performed to evaluate the systemic and as well mucosal antibody responses to the candidate vaccine.

Samplings will be done at different time points during the study period (see schedule in section 2.2).

In addition, the immune response induced by the vaccine will be compared with the immune response following challenge in both the vaccine and the placebo group (samplings during both vaccination and challenge phase are described in the schedule of events, section 2.2).

Shedding of the 2457T challenge organism might be compared between vaccine and placebo group from stools collected during the inpatient period. In addition to classical bacterial cultures, shedding will be documented by qPCR analysis from stool samples frozen at -80C. If required, analysis of baseline samples might be extended to stools collected at C-1 and/or C0.
As exploratory test, lactoferrin might be investigated in post-challenge stools from vaccine and placebo groups, since this relates to presence of WBC and is therefore considered as a potential marker of inflammation.
Such an exploratory analysis, including qPCR and quantitative stool cultures, aims to identify if these tests could be used as predictors of illness in the challenge model.
Functionality of the 2a-specific antibodies induced by vaccination will be evaluated and the possibility to obtain a candidate correlate of protection from the immune parameters analyzed will be investigated.

7.0 INVESTIGATIONAL PLAN TRIAL DESIGN

7.1 Trial design

This Phase 2b trial will be conducted as a randomized, double-blind, single-center, repeated dose study to evaluate the efficacy of the Flexyn2a bioconjugate candidate vaccine in healthy naïve subjects of 18-50 years of age.

Study participants will be recruited by phone, email or public advertisements.
All study-related activities will be started only after subject's consent is documented by signature on the Informed Consent Form (ICF). Some procedures may be done under the JH200 screening consent. The study-specific screening of potential subjects will be conducted as early as 30 days before start of enrolment.

Two phases will be carried out: an initial vaccination phase followed by a challenge phase. Two groups of 36 subjects will be enrolled successively, and up to 30 subjects from each group will be consecutively challenged.

In the vaccination phase, subjects will be administered the candidate vaccine or placebo on Day 0 and Day 28. All vaccinations will be given by intramuscular injection in the deltoid muscle.
About 1 month after the second administration, the vaccination phase will be closed and followed by the challenge phase.
All subjects not challenged will have a final study visit at the end of the vaccination phase at about Day 56 (defined as outpatient V5 in section 2.2).
Up to 30 subjects per group will be admitted to the in-patient unit and challenged with the fully virulent *S. flexneri* 2a 2457T strain. After challenge, subjects will be monitored for 5 to 10 days for diarrhea and other signs or symptoms of enteric illness and will receive all the necessary treatments and clinical care by the study team.
The last study visit will occur about 1 month following challenge (defined as outpatient V6 in section 2.2). Approximately 6 months after challenge, a post-study follow-up call will occur in order to capture any new chronic health conditions, serious health events or hospitalizations and evaluation of bowel symptoms using the Rome III criteria. Subjects who did not proceed to challenge will be contacted about 6 months after their last vaccination to capture any new chronic health conditions, serious health events or hospitalizations.

A post-study follow-up visit will be performed to test antibody persistence at about one year following vaccination. However, participation in this visit will be considered optional and if this visit is missed or done outside of the window it will not be considered a protocol deviation.

Sixty subjects are considered an adequate number for an initial efficacy evaluation of protection. The trial is double-blinded so that unbiased reporting of safety-relevant events from the subjects as well as unbiased assessments from investigators can be ensured.

7.2 Beginning and end of the trial

Each subject is considered to be enrolled in the trial when he/she meets all eligibility criteria, is randomized and receives at least one dose of study treatment.

Subjects who sign the informed consent, but never receive the vaccine treatment are considered a screening failure.
Each subject is considered to have ended participation in the trial when he/she has completed the last protocol-specified contact (phone call) or prematurely discontinued the study. The end of the study is defined as the date on which the last patient completes the last follow up phone call.
A subject is considered to have discontinued after he/she has withdrawn consent or has been discontinued under the conditions specified in Section 9.3.3.
A subject is considered to have been lost to follow-up if he/she is unable to be contacted by the investigator and come to the clinic. The end of participation for a subject lost to follow-up is the last known contact (e.g., visit or telephone contact).
The overall trial begins when the first subject has received the first injection. The overall trial ends when the last remaining subject has ended participation in the trial, by completing the trial, being discontinued from the trial, or being lost to follow-up.

Each subject will be monitored for the occurrence of AEs immediately after receiving the first dose of investigational product or placebo and up to the last study visit. Detailed follow-up procedures are given in section 10.5, 10.8 (for AEs with late onset) and 10.9 (for pregnancy).

A subject is considered to have completed study drug dosing, if the subject has received the second injection.

Subject’s trial-participation will range from approximately 8 to 10 months, from the time the subject signs the ICF for pre-screening, through the final follow-up phone call.

7.3 Trial Population

In this trial, only healthy adult volunteers (male or female) will be included.

7.3.1 Subject inclusion criteria

A subject must meet **all** criteria listed below to be included in the study:

1. Male or female age 18-50 years (inclusive)
2. Good health, without clinically significant medical history or physical examination findings.
3. Negative serum pregnancy test at screening, and negative urine before each vaccination and before challenge for female subjects of childbearing potential.
4. Females of childbearing potential must agree to avoid pregnancy by use of effective contraception. Abstinence is not acceptable as effective contraception. Female subjects unable to bear children must have this documented (e.g. tubal ligation or hysterectomy).
5. Willingness to participate in the study after all aspects of the protocol have been explained and written informed consent obtained.
6. Completion of a training session and demonstrated comprehension of the protocol procedures, knowledge of Shigella-associated illness, and a passing score of 70% or better on a written examination (comprehension test, described in section 9.1.1).
7. Availability for the study duration, including all planned follow-up visits.

8. Willingness to refrain from participating in other studies of investigational products until completion of the last study visit.

7.3.2 Subject exclusion criteria

A subject meeting **any** of the exclusion criteria listed below must be excluded from participating in the trial:

1. Women currently nursing.
2. Presence of a significant medical or psychiatric condition which in the opinion of the investigator precludes participation in the study.
3. Clinically significant abnormalities in screening hematology or serum chemistry as determined by PI or PI in consultation with the research monitor and sponsor.
4. Presence in the serum of HIV antibody, HBs-Ag, or HCV antibody (if confirmed positive by HepC confirmatory test, i.e. RIBA, PCR)
5. Evidence of IgA deficiency (serum IgA < 7 mg/dl or limit of detection of assay).
6. Evidence of current excessive alcohol consumption or drug dependence.
7. Evidence of impaired immune function.
8. BMI <19 and ≥35
9. Recent vaccination or receipt of an investigational product (within 30 days before vaccination and until last study visit).
10. Personal history of an inflammatory arthritis.
11. Positive blood test for HLA-B27.
12. Personal history of irritable bowel syndrome as defined by Rome III criteria.
13. Treatment with immunoglobulins or blood products within 3 months from first candidate vaccine injection.
14. Regularly abnormal stool pattern (fewer than 3 per week or more than 3 per day) or loose or liquid stools
15. Regular use of laxatives, antacids, or other agents to lower stomach acidity.
16. Use of any medication known to affect the immune function (e.g., systemic steroids) within 30 days preceding the first vaccination or planned use during the entire study period.
17. Known allergy to any of the following antibiotics: ciprofloxacin, trimethoprim-sulfamethoxazole or penicillin.
18. Symptoms consistent with Traveler’s Diarrhea concurrent with travel to countries where Shigella infection is endemic (most of the developing world) within two years prior to dosing, OR planned travel to endemic countries during the length of the study.
19. Vaccination for or ingestion of Shigella within 3 years prior to vaccination.
20. Use of antibiotics during the 7 days before vaccination and challenge
21. Use of proton pump inhibitors, H2 blockers or antacids within 48 hours prior to challenge.
22. Serum IgG titer to Shigella flexneri 2a LPS ≥ 2500
23. Current occupation involving handling of Shigella bacteria
24. History of allergy to any vaccine or to soy

25. Any other criteria which, in the investigator's opinion, would compromise the ability of the subject to participate in the study, the safety of the study, or the results of the study

7.3.3 Subject Replacement

Subjects who sign the ICF, but withdraw study participation before the first administration, whether or not they have been randomized, will not be followed up. These subjects will be replaced.
Subjects will not be replaced if they were administered study product (vaccine or placebo) before they withdrew consent to participation.

7.3.4 Eligibility for injection

At screening and at visit 1 (Day 0) subjects' inclusion and exclusion criteria will be checked (including at V1 laboratory results obtained from screening analysis) and eligibility of the subjects to participate in the trial confirmed.
Women of child bearing potential have to agree to use effective contraception for the duration of the study.

7.3.5 Treatment allocation

The study is double-blind. Healthy volunteers, site personnel and monitors will be blinded to the treatment being given. The investigational pharmacist, pharmacy delegated staff and the sponsor will be unblinded.
A treatment key, listing the specific formulation corresponding to each randomization number, will be provided by the Contract Research Organization (CRO) to the pharmacy and kept in a safe location with restricted access at the pharmacy. Blinding of the subjects will be maintained throughout the whole duration of the study (until the last follow-up call at day 240).

7.3.6 Breaking the blinding

According to international guidelines, the treating physician (investigator) is responsible for the medical care of the individual trial subject (Declaration of Helsinki and ICH E6 (R1) 4.3). The blinding can be broken by the research pharmacist upon request of the principal investigator or of the independent Research Monitor. If the blind is prematurely broken, it is the responsibility of the investigator to promptly document and explain any unblinding to the sponsor within 1 working day.
The study blind should not be broken except in a medical emergency (medical events which the investigator/physician in charge of the subject feels cannot be treated without knowing the identity of the treatment) or regulatory requirement (e.g., for SUSARs or death). Any unblinding by study site personnel will be documented in the eCRF, and statistical analysis will examine the potential impact of the unblinding. If the blind is broken, the date, time, and reason must be recorded in the patient's eCRF and any associated AE report. All unblinding events will be reported to the Research Monitor and the sponsor.

If an investigator, study site personnel performing assessments, or participant is unblinded, the participant must be withdrawn from the study and procedures accompanying withdrawal will be performed. In cases where there are ethical reasons for the participant to remain in the study, the investigator must obtain specific approval from the sponsor or their designee for the patient to continue in the study.
Suspected unexpected SAEs, which are subject to expedited reporting, will be unblinded before submission to the regulatory authorities.

7.4 Trial schedule

The visit-by-visit overview of trial activities is summarized in Section 2.2 and detailed in Section 9.1.

Visit windows prior to, and including Challenge will be based on the V1, Day 0 date of enrolment. Subsequent visit windows will be based on the V5 date of Challenge.

All visits should be compliant with the windows specified in Section 2.2. Every attempt should be made to have each subject attending each visit as scheduled. However, if a subject is unable to attend a visit within the specified compliance times, the visit should be scheduled as closely as possible to these windows. Efforts should be made to avoid missed visits.

8.0 STUDY DRUG ADMINISTRATION

Administration of study drug (vaccine) or placebo must be started on the same day in which randomized treatment is assigned.

8.1 Flexyn2a candidate vaccine

GlycoVaxyn has developed Flexyn2a, a candidate bioconjugate vaccine which consists of the *S. flexneri 2a* polysaccharide O-antigen, conjugated to a detoxified EPA protein carrier. This bioconjugate is produced *in vivo* in *E. coli* and subsequently extracted and purified. Characterization of the bioconjugate is based on standard methods, such as nuclear magnetic resonance (NMR), high-performance liquid chromatography (HPLC) and mass spectrometry (MS).

For any additional information regarding the study product, please refer to the Investigator Brochure of Flexyn2a.

8.2 Study drug formulations and supply

Table 1 below presents the composition of the injection formulation that will be provided to the pharmacy of the study site.

Table 1: Injection formulation for each treatment arm

Arm	*Vaccine name*	*Formulation*	*Injection's volume*	*Quantity Doses / subject*
A	Flexyn2a	10 µg 2a-EPA	0.5 ml	2
B	Placebo	Saline buffer	0.5 ml	2

The research pharmacy will receive clinical supply material to prepare 2 injections for each subject and additional vials for each treatment arm to prepare extra injections as replacements in case of breakage, bad storage, loss or any other reason that would make the product unusable.
All delivered vials must be recorded and accounted for on the appropriate drug accountability log (included in the study-specific procedure, or SSP, for receipt and return of study product).

8.3 Study drug preparation

In order to keep investigators blinded on the treatment subjects do receive, study drug will be formulated by the assigned pharmacists of the study site and then provided to the study team as ready to use, blinded to treatment and identified with the study ID of the subject and the subject's initials. Both study drug and placebo are transparent and visually not distinguishable.
Briefly, before administration into subjects, vials should be taken out of the refrigerator and placed at ambient conditions, in a place protected from direct sunlight to warm up to room temperature. The subject identification (ID) number should be recorded on the used vials and on their box. After formulation, used vials are put back into the box in the refrigerator for accountability.
Vials containing Flexyn2a need to be diluted. The appropriate volume of vaccine from the vial stock solution must be transferred to the vial containing placebo to generate the correct vaccine formulation for injection. Before and after transfer, solutions should be gently mixed by inverting the vials several times.

In order to guide site personnel to the appropriate preparation of the study product, more detailed information is included in the SSP for formulation of study product.

8.4 Study drug administration

Subjects will be administered one dose (0.5 mL) of candidate vaccine or placebo at Day 0 and about Day 28. Both doses will be administered into the subject’s deltoid muscle of the same arm.

8.5 Study drug storage

All vials must be stored at the pharmacy of the study center in a safe and locked place with access restricted to authorized personnel only.
Vials must be stored within the predefined temperature range between +2 and +8 °C and protected from light.
The storage temperature of study drug vials will be under continuous automatic recording and will be monitored daily using validated temperature monitoring devices. Temperature measurements will be recorded on a dedicated log, during working days, preferably about the same time of the day. A SSP describes the procedure for reporting temperature deviation to the sponsor. Following exposure to a significant temperature deviation (as described in the SSP), the study vaccines will not be used until official approval is received from the sponsor.

8.6 Replacement of unusable vials

In addition to the vials provided for the planned number of subjects, additional vials will be provided to replace any unusable doses.

In case of a vial that is broken, unusable or lost, the pharmacist can replace it with a different vial. The pharmacist must notify the sponsor and the use of replacement vial must be recorded on the drug accountability log.

8.7 Labeling and packaging

Each vial and box containing the study drug or placebo will be labeled for identification with a primary and a secondary label, respectively. The extent of information that has to be written in each label is described in the Investigator Brochure of Flexyn2a.

A blank space is introduced in the primary label to record the subject identification number that has to be written with an indelible pen.
Vials will be provided in so called “subject packs”, which contain all solutions to prepare 2 doses of the same drug formulation (Flexyn2a or placebo) for each subject. The used vials will be stored back in the subject pack, to facilitate final accountability and reconciliation.

9.0 TRIAL CONDUCT

9.1 Trial visits

9.1.1 Recruitment of Subjects/Pre-screening

Healthy subjects will be recruited for this study. Potential subjects will respond to posted flyers, website postings, and newspaper advertisements by telephone call or email to the clinical trial site.

The CIR will initially use a screening protocol approved by the Johns Hopkins School of Public Health (JHSPH) Institutional Review Board (IRB) in recruiting volunteers for this study. The screening protocol is entitled “Screening of adult volunteers for eligibility to participate in clinical studies evaluating investigational vaccines, antimicrobial agents, or disease prevention measures or the pathogenesis of infectious agents” JHSPH IRB 200, JHSPH IRB H.22.04.02.19.A2.

Study staff will respond to inquiries by phone. Study staff will provide a brief, general description of the research study that includes planned start date, outpatient vaccinations followed by a 10 day inpatient phase, shigellosis-study and any significant exclusion criteria, in compliance with the CIR IRB approved JH200 screening protocol. Subjects who express interest in participating in the study will be asked to complete a telephone pre-screen to assess general health status and basic eligibility. Potential volunteers determined to be generally healthy and meeting basic eligibility requirements are scheduled for an in-person screening.

Volunteers will be made aware that the screening process may take several visits to complete.

The following screening procedures will be performed with each prospective volunteer.

1. Discuss the study screening process and obtain Screening Informed Consent from the subject.

2. Ensure that the subject has signed and received a copy of the ICF and has a clear understanding of the nature of the screening study by passing a brief comprehension examination.

3. Explain the HIV testing process and ensure that subject has signed the HIV Information sheet.

4. Complete medical history/exam and a series of clinical laboratory tests to rule out occult illness, pregnancy and prior possible infections with Shigella bacteria or travel in developing countries where Shigella is endemic. Laboratory tests will include, but are not limited to, *S. flexneri*-prior exposure serological testing.

Subjects will be provided a stool collection kit and instructions, and will be asked to bring a stool sample with them on the day of their study-specific-screening visit, collected within about 8 hours of presentation to the unit. Subjects may also provide the stool sample upon arrival. Priority for enrollment will include subjects who were able to produce a baseline-stool either at screening or at enrollment, however it will not be exclusionary for participation in the study.

Explain Trial, Comprehension Test, and Obtain Written Informed Consent

All subjects will undergo an informed consent process prior to the start of any study specific procedures. Informed consent is an ongoing process which includes the informed consent document. Subjects will receive an oral presentation of the study. Each prospective subject will be given the written, IRB-approved informed consent, allowed ample time to read the consent, allowed to ask questions about the study, have his/her questions answered, and given time to decide if he/she would like to participate in the study. To document subjects' understanding of informed consent, immediately before the consent is signed, the person obtaining consent will administer a brief quiz or comprehension test. Incorrect answers will be discussed with subjects to reinforce the consent and subjects will be given one additional opportunity to take the test. A final acceptable test score is 70% or more answered correctly. Subjects failing after 2 attempts are not eligible for study enrollment. No coercion or influence is allowed in obtaining subjects' consent. Before subjects participate in the study, consent forms will be signed and dated by subjects as well as by the PI or designee. Subjects will receive copies of the signed consent prior to participation. As part of the consent process, subjects will also be asked to read and sign a Medical Records/Lab Results Release, with an opportunity to ask questions, if relevant.
Any subject who, in the opinion of the study staff and/or PI, does not understand the study well enough to consider his or her consent truly informed will be excluded.

The informed consent process will include, briefing material, an informed consent document and a quiz. Throughout the study, the clinical team will address any questions and concerns of the subject.

9.1.2 Study Specific Screening (Days -30 to -2)

A study-specific screening visit is scheduled between -30 to -2 days before the first vaccination.

Every subject who provides informed consent will receive a Screening number. Basic subject information, plus the screening number will be recorded on the Screening Log.

The following evaluations/procedures will be carried out:

- Review Inclusion/Exclusion Criteria and ICF

The inclusion and exclusion criteria will be reviewed by the investigator or qualified designee as well as before each dose of vaccine and prior to challenge to ensure that the subject qualifies for the administration of the study drug (see section 7.3.1 and 7.3.2 for a detailed list of the criteria) or for challenge.

- Review/Obtain Medical History

A complete medical history will be obtained, reviewed and updated by study staff, and reviewed by the investigator or qualified designee with the subject and maintained in the subject' records.

- Stool collection for immune-assays baseline

Subjects will be provided a stool collection kit and instructions, and will be asked to bring a stool sample with them on the day of vaccination, collected within about 8 hours of presentation to the unit. Subjects may also provide the stool sample upon arrival. Priority for enrollment will include subjects who are able to produce a stool within this timeframe; however will not be exclusionary for participation in the study.

- Physical examination
- Vital Signs
- Laboratory analysis:
  - Complete blood count
  - Serum transaminases (ALT/AST)
  - Na+, K+, CL-, HCO3-, glucose, BUN and creatinine
- Urine toxicology
- Pregnancy test for women (serum β-hCG)
- HLA B27 antigen
- Total IgA
- Hepatitis B antigen, hepatitis C antibody, HIV-1 antibody
- IBS survey

9.1.3 Visit 1: Enrollment (Day 0) and 1st injection

During V1, the following steps will be performed:

- Re-confirmation of subject eligibility (review of inclusion/exclusion criteria)
- Recording of any concomitant medication/treatment and/or vaccination
- Review any new or change in medical history
- Physical examination and vital signs
- Collection of stool sample for baseline immune parameters
- Urine pregnancy test for female subjects
- Blood collection for:
  - chemistry and hematology
  - baseline serum antibody response against *S. flexneri* 2a
  - baseline mucosal immunity and cellular immune-response

- Randomization
- Injection

After re-assessment of the screened subjects and confirmation of eligibility a subject identification number (independent from the screening number) will be assigned and entered into the AdvantageEDC for randomization (refer to the AdvantageEDC User's Guide for instructions).

The subject identification number (ID) will not have the name of the participant and will be used to identify the subject during the trial on all trial documents and samples.

In the event that site access to the Internet is unavailable, the AdvantageEDC enrollment system is unavailable, or there is a problem with the function of the system, Back-Up Randomization procedures are provided in the Data Management Handbook. All subjects enrolled in the study will be entered into the AdvantageEDC Internet Data Entry System (IDES).

Randomization should take place just prior to product administration, but after the subject has signed the IRB/IEC-approved consent form, undergone the screening process, and met all of the inclusion and none of the exclusion criteria.

AdvantageEDC requires the entry of demographic information and completion of the Eligibility Checklist to confirm that the subject has met all of the inclusion criteria and none of the exclusion criteria. Through AdvantageEDC subjects will be assigned a sequence number and a treatment number, the treatment number will correspond with the product the subject will receive. Only the investigational pharmacist and pharmacy staff will have access to this information (see section 7.3.5).

Sequence Number and Treatment Number will be displayed on the Enrollment Confirmation screen. The Sequence Number and Treatment Number should be recorded next to the Subject ID on the Enrollment Log, and the Enrollment Confirmation screen should be printed and provided to the pharmacist who will be preparing the study product for administration. A copy of the Enrollment Confirmation screen should be maintained in the subject's source documents files.

The pharmacist will reference the Treatment Key (see section 7.3.5) to determine whether the subject was randomized to receive vaccine or placebo. Then, the pharmacist will record the Subject ID on the Treatment Key next to the Treatment Number, as well as the date for Dose 1. The pharmacist will prepare the product (vaccine or placebo), in a blinded fashion as per SSP and label the syringe with the subject ID and subject initials and provide to clinic staff.
When the subject returns for Dose 2, the pharmacist references the Treatment Key again and records the date for the Dose 2.

A vaccination-documentation will be completed by the clinical staff during the first and the second vaccination of each subject and will include at a minimum the following information:

- Subject ID
- Date
- Treatment Number
- Administration time and site

Injections will be administered intramuscularly, into the deltoid muscle, with either vaccine or placebo as per study specific procedure.

After vaccination all subjects will remain on the unit for monitoring for at least 30 minutes.

During this time, subjects will be provided a memory aid, measuring tool and thermometer and instructed on use. Study diaries are provided to record body temperature, solicited local and general AEs, and unsolicited AEs. The subject will return the memory aid at the following visit on Day 7.

After 30 minutes subjects will have vital signs taken and be assessed for any AE's. This information will be recorded in the subjects' records. Prior to discharge subjects will have questions answered, be provided with subject ID card (with contact numbers of the clinical site to call in case of questions) and a visit card with information regarding their follow up call and next planned visit.

9.1.4 Follow up calls (Day 1 and Day 29)

The day after each injection, participants will be contacted by phone to collect any new or change in signs or symptoms (AE's) they are experiencing and any new or change in medications, this will be recorded in the subject's record. They will be reminded to complete their diary, take their temperature and if febrile to call the coordinator. They will also be reminded of their next visit date.

9.1.5 Visit 2 (Day 7)

About seven days after the first vaccine dose, participants will return to the center and the following steps will be performed:

- Subject's memory aid will be collected, reviewed and signs or symptoms will be recorded (should the visit occur six days after first vaccine dose, the day 6 of the memory aid will be completed at the site during the visit)
- Clinical assessment including vital signs
- Review of any new or change in AE's and medications
- Bloods collection for chemistries, hematology, and immunological assessments as per schedule of events
- Date of next visit is scheduled
- Subjects are provided a stool collection kit and instructions, and are asked to bring a stool sample with them on the next visit (V3), collected within about 8 hours of presentation to the unit. Subjects may also provide the stool sample upon arrival.

9.1.6 Visit 3 (Day 28): 2nd injection

Subjects will return to the center for their second injection and the following steps will be performed:

- Pertinent inclusion/exclusion criteria will be reviewed to assure subjects remain eligible to continue in the study
- Check contraindications to vaccination (see section 9.3.4)

- Females will have urine pregnancy testing completed prior to vaccination and test must remain negative
- Subjects will undergo physical examination and vital sign assessment, review of any new or change in AE's and medications they are or have been taking
- Blood will be obtained for chemistries, hematology and immunological assessments as per schedule of events
- Stools will be collected for immunoassays

If the subject remains eligible, he/she will proceed to the second vaccination. The research pharmacist will prepare the investigational product for administration of either vaccine or placebo based on the treatment subject has been randomized on V1.

Subjects will be administered with either vaccine or placebo as per study specific procedure.

After vaccination all subjects will remain on the unit for monitoring for at least 30 minutes. Subjects will be provided a memory aid and instructed on use. After 30 minutes subjects will have vital signs taken and be assessed for any new AE's. This information will be recorded in the subjects' records. Prior to discharge from the unit subjects will have questions answered and be provided with information regarding their follow up call and next planned visit.

9.1.7 Visit 4 (Day 35)

About seven days after the second injection, subjects will return to the center and the following steps will be performed:

- Subject's memory aid will be collected, reviewed and signs or symptoms will be recorded (should the visit occur six days after the second injection, the day 6 of the memory aid will be completed at the site during the visit)
- Clinical assessment including vital signs
- Review of any new or change in AE's and medications
- Blood collection for chemistries, hematology and immunological assessments as per schedule of events
- Subjects will be provided stool collection supplies and collection process will be reviewed, subjects will be asked to bring a sample the day of admission collected within 8 hours of presentation for admission to the inpatient unit.
- Date of next visit is scheduled

9.1.8 Visit 5: Challenge

9.1.8.1 Admission Challenge, Day C-1

Before admission, a list with all subjects IDs to be included for the challenge (with vaccine and placebo recipients equally distributed if possible) will be provided to the study team by the CRO (detailed procedure will be given in dedicated SSP).

The day of admission, subjects will be evaluated to ensure no exclusionary conditions have arisen and baseline chemistries and hematology exams and laboratory evaluations will be obtained.

Subjects will undergo vital signs assessment, review of medical history, and physical examination. AE's and con-meds will be assessed.

A serum pregnancy test (βhCG) will also be obtained from women of childbearing potential. If the result of a serum pregnancy test is not available on day of challenge, a urine pregnancy test will be conducted.

Additionally, samples will be collected for assessment of immune outcome (and exploratory) measures as described in schedule of events.

Stool will be collected for bacteriology, immunology and exploratory assays as per schedule of events. In the event a subject is unable to produce a stool at admission, stool may be collected anytime the day of admission or on day C0, latest 4 hours after challenge. Subject's inability to produce a stool will not be exclusionary for challenge, but it will be considered a protocol deviation for this study.

One alternate may be admitted overnight to replace anyone who may not continue to meet eligibility criteria in the morning.

9.1.8.2 Challenge Day, Day C0

On the day of challenge, subjects will receive a light breakfast and then initiate at least a 90 minute fasting period.

Prior to challenge subjects will have vital signs, a physical exam and evaluation to assure that there are no changes from admission to ensure no exclusionary conditions have arisen.

Approximately 1 minute prior to challenge, subjects will drink 120 mL of bicarbonate buffer (buffer formulation: 13.35 gram of sodium bicarbonate in 1000 mL of sterile water for irrigation).

For challenge, subjects will drink a solution of virulent *Shigella flexneri* 2a bacteria (strain 2457T) suspended in 30 mL of bicarbonate buffer including inoculum dose. Subjects will continue fasting for at least an additional 90 minutes post challenge. Then there will be no dietary restrictions.

The alternate or a subject who does not continue to meet eligibility criteria will be discharged without challenge and follow the schedule for vaccinees only.

9.1.8.3 Inpatient monitoring

Subjects will remain at the inpatient facility under clinical observation. Vital signs will be assessed at least 3 times each day, once in the morning, in the afternoon and at bedtime.

On challenge day, vital signs will be assessed 4 times, once prior to challenge, once about 30 minutes after challenge, and then 2 additional times this day.

As demonstrated by previous challenge studies with this and similar bacterial strains the optimal care of the volunteers during the acute challenge period involves very close monitoring for signs and symptoms of volume loss, and aggressive hydration using both oral and IV fluids as necessary.

During the inpatient period a clinician will closely monitor the volunteers and conduct a daily focused PE and medical interview to assess health status, follow-up, monitor, and treat as indicated.

Blood, stool and urine will be collected as per schedule of events. Additional blood and urine samples may be obtained at the PI's discretion. As laboratory assessments (e.g. chemistries, blood counts) are delayed by several hours from the time of collection to result reporting, they are not generally as useful in the acute management of ill subjects. They are reserved for subjects for whom the course of illness is outside the expected range or for any follow up needed (for example, someone who presents with orthostatic hypotension that is not responsive to fluid or that takes longer to resolve than expected).

All stools will be collected for weighing and grading. Following Shigella challenge, up to 3 stool samples (or alternatively a rectal swab) will be collected daily for culture starting the day after challenge. The subject needs 2 consecutive stool cultures negative for Shigella and to have taken two doses of antibiotic with resolved or resolving clinical symptoms before discharge. Remaining doses of antibiotic will be given to the subject for self-administration. Once a subject has two negative stools for culture no further stool samples will be required for culture.

Routine discharge is scheduled about 8 days from challenge. Early discharge is permitted in cases where early antibiotic treatment has been initiated (at least two doses taken), the subject is feeling well (clinical symptoms are resolved or are resolving) and the subject has at least two negative stools for the Shigella. Vital signs and a physical exam will be completed prior to discharge. The subject will be provided any remaining doses of antibiotics and instructed use for completion of the 3 day dosing regimen.

If early discharged, subjects may be required to return at about Day C7 to complete a follow up visit which will include, review of AE's and con meds, vital signs, a focused physical examination, and blood and stool samples as per schedule of events.

9.1.8.4 Clinical Management

Antibiotic treatment

Antibiotic treatment after challenge will be administered according to criteria for early antibiotic treatment (described below) or 5 days after challenge if subjects do not meet the criteria for early treatment.

Early antibiotic treatment after challenge will commence when any of the following criteria are identified and a study physician considers it to be warranted:

• When volunteers meet the primary endpoint (clinical definition of shigellosis)

• Oral temperature ≥ 39°C

• Any other reason warranting the early treatment in the physician's opinion

Routine treatment will commence at about 120 hours (on the morning of day 5) post-challenge. If, because of illness, a subject is unable to take oral antibiotics, intravenous antibiotics may be given (IV ciprofloxacin at an appropriate dose based on weight and clinical status).

Subjects will be treated with ciprofloxacin (500 mg by mouth twice daily for 3 days), trimethoprim 160 mg / sulfamethoxazole (800 mg by mouth twice daily for three days), or amoxicillin (500 mg by mouth 3 times daily for 3 days).
Symptoms infrequently associated with ciprofloxacin include nausea, vomiting, diarrhea, abdominal pain, rash, headache or restlessness. Amoxicillin is generally well tolerated and the most frequently reported adverse reactions are diarrhea and rash.
Trimethoprim-sulfamethoxazole is also well tolerated; common adverse reactions are nausea, vomiting, diarrhea and loss of appetite may occur.

Treatment for vomiting
Subjects who are vomiting may be given ondansetron (Zofran) ODT or ondansetron IV for the management of vomiting.

Rehydration Procedures
Subjects passing grade 3-5 stools post-challenge will be offered Oral Rehydration Solution (ORS), an oral glucose/electrolyte solution or other oral rehydration solution i.e. Gatorade to prevent dehydration, at the same volume as their stool output.

ORS CeraLyte 50 (CeraProducts, Jessup, MD) is a rice-based oral electrolyte solution that will be used to help control/prevent dehydration among subjects experiencing diarrhea. CeraLyte 50 is packaged in 10 g sachets to be dissolved in 200 mL (~ 1 cup) tap water. The contents per 10 g sachet are as follows:

- Sodium chloride - 230 mg
- Potassium chloride - 156 mg
- Trisodium citrate - 378 mg
- Carbohydrates - 9.4 g

On a per-liter basis, CeraLyte 50 provides 50 mEq of sodium chloride and 20 mEq of potassium at a mOsm < 250.
For documentation purposes of concomitant medications, ORS or other oral rehydration solutions will not be considered a concomitant medication while IV fluids will.

A subject may be administered IV fluids (clinician discretion) if they:

- Experience abrupt onset of diarrhea, as determined by PI or designee
- Become hypovolemic, defined as confirmed supine systolic BP < 90 mmHg and associated symptoms, or significant lightheadedness on standing, with a confirmed postural change in BP or pulse. Postural vital signs will be measured lying and 2 minutes after standing. A significant change is a decrease in systolic BP of > 20 mmHg, or diastolic BP of > 10 mmHg or increase in pulse of > 30 beats/minute
- If determined necessary by the study physician (i.e. diarrhea with nausea/vomiting and unable to drink enough to keep up with output)

9.1.9 Visit 6: final study-visit

About 28 days after challenge, subjects will have outpatient visit in the clinic, on about day 84 (V6).

Any subject enrolled in the study but not challenged (excluding subjects that were withdrawn) will have a final study visit on Day 56 (corresponding for these subjects to outpatient V5), about 28 days from the second injection.

The final visit will include vital signs assessment, clinical checks with focused physical examination (if indicated according to PI's judgement), assessments of any new or ongoing AE's and concomitant medications, stool and blood sample collection for immunogenicity and exploratory outcome evaluation as per schedule of events. Females of childbearing potential will have a urine pregnancy test.

9.1.10 Follow up phone-call P3

Subjects enrolled in the study but not challenged, will have a final follow up phone call about 6 months from their last injection. All the challenged subjects will be contacted by phone approximately 6 months after challenge.

Subjects will be asked about any new chronic illnesses, vaccinations, new serious health events or hospitalizations.

In addition, subjects who received challenge will be asked about any symptoms of reactive arthritis and will be asked to complete Rome III questionnaire (IBS survey). Any subject with significant findings may also be contacted again for follow up by the investigator or designee.

This phone call is not considered to be part of the subjects' active study participation.

9.1.11 Visit 7: Optional long term follow-up visits (2 visits)

Subjects will be contacted to see if they would be willing to return for optional one or two long-term follow-up visits. At the initial visit, females will have urine pregnancy test, and in all subjects, blood will be drawn to check for CBC and the immune response against the Shigella LPS. Subjects selected according to their antibody titer who have a minimum hemoglobin of 12g/dL will be invited back for a second blood draw to collect additional blood and a stool sample if possible. No more than 450 mL of blood will be drawn between the 2 visits in order to collect serum and PBMCs to better characterize the antibodies of these subjects and also to create serum controls for future assays. If necessary, blood draws will be performed with the subjects on an exam table (similar to what occurs during blood donations). Subjects will be monitored by a nurse during these blood draws. Vital signs will be checked before and after the draw, and subjects will be encouraged to drink both before and after the blood draw. Vital signs will be recorded in the source documents of this visit.

9.1.12 Unscheduled visit (UV)

At any time during the study, the subject may be asked to visit the investigator for any reason. During this unscheduled visit, the investigator should perform the following steps:

- Review of pre-existing medical records (if indicated)
- Physical examination and vital signs (if indicated)

- Recording of AEs
- Recording of SAEs
- Recording of any concomitant medication/treatment or vaccination

The investigator will then record the reason of this unscheduled visit and the results of the visit assessments in the source files. The data will be then transcribed into the eCRF as required.

9.2 Trial Procedures

9.2.1 Stools grading

Stool will be graded based on a standard stool grading scale as follows:
Grade 1 = Fully formed (normal)
Grade 2 = Soft (normal)
Grade 3 = Thick liquid (diarrheal)
Grade 4 = Opaque watery (diarrheal)
Grade 5 = Rice-water (diarrheal)

During inpatient phase stools will be collected every day until discharge and immunology assays will be performed on 3 time-points.

9.2.2 Physical Examination

A general health focused physical examination will be performed prior to receipt of investigational products and daily during the inpatient phase, follow up visits may include a PE at the physician's discretion. All new or change in findings and their clinical significance will be reported.

9.2.3 Vital Signs

Vital signs, including oral temperature, heart rate and blood pressure, will be assessed and recorded at every clinic visit. Every subject will receive a digital thermometer to keep track of the oral temperature at home once a day, namely between Day 0 and Day 7, and between Day 28 and Day 35. Subjects will be instructed to retake temperature if found to be ≥ than 100.4 °F (38°C) to be confirmed after resting for about 20 minutes, with no eating, drinking or smoking. If in the opinion of the PI the repeat temperature is thought to be a more accurate reflection of the subject's temperature only the repeated temperature will be recorded in the eCRF. During the inpatient challenge, vital signs will be measured and recorded in the source documents at least 3 times a day, except on the day of challenge when they will be measured 4 times. Vital signs will be recorded in the eCRF for each outpatient visit until V6; for the inpatient visit, they will be recorded on the day of admission, the day of challenge prior to challenge and approximately 30 minutes after challenge, and prior to discharge. The remaining vital signs will not be recorded into the eCRF unless they are abnormal and judged by the investigator to be clinically significant.

9.2.4 Follow-up process

All subjects should stay in the study until the last follow up phone call. If a subject does not appear for an assigned visit, every effort should be made to contact him/her to

confirm that the subject is well and to re-schedule the missed visit. Acceptable attempts include at least 3 phone calls and/or text message attempts followed by sending of a certified letter. This effort must be accurately documented into the source documents and monitored by the CRO.

Subjects will be given the option to return for one or two long-term follow-up visits. These visits are specifically for immunology measurements. On the first visit, subjects will be invited back to the clinic, re-consented to the current protocol, if female, have a pregnancy test, and then have an initial blood draw. This blood draw will be done for CBC, chemistry, serology and collection of PBMCs. They will also have a blood type done and be screened for HIV, Hepatitis B and Hepatitis C. According to their levels of antibodies anti Shigella-LPS and hemoglobin, they may be requested to return for a second blood draw as discussed above.

9.2.5 Blood samples

Handling and analysis
Samples collected under this protocol will be used to conduct protocol-related safety and immunogenicity evaluations. Samples for immunogenicity will be collected at the JHU CIR and maintained until transport to laboratories at WRAIR or JHSPH. Storage at WRAIR or JHSPH of these biological samples will be handled according to appropriate procedures as per their standard operating procedures or per SSP's. All subjects will consent for the future use of their specimens.

Collection
Blood should be collected observing the appropriate conditions and procedures in use at the clinical site. The investigator must ensure that the study personnel and the laboratory staff are compliant with this requirement.

Labelling
Blood samples will be labelled with at least the following information using pre-printed special temperature safe labels and/or an indelible ink following the SSP:

- study number
- subject ID
- date of draw (DD-MMM-YYYY)
- visit number

Sorting and storage
The tubes must be appropriately labelled and placed into boxes for storage and shipment. The tubes containing blood or serum must be stored in a vertical position. A biological specimen log, specifying the samples being shipped for each individual subject at each time point of the study, must be maintained.
Serum samples will be stored at the facilities of the immunology laboratory for up to 15 years. Shipment tracking forms must be completed and copies kept in the study file as specified in the study-specific procedures. In addition, aliquots of immunological samples will be delivered to the sponsor under appropriate conditions. A more detailed description of procedures will be included in a SSP developed for this study.

9.2.6 Laboratory assays

Screening, safety and microbiology assays will be performed in qualified local laboratories using standardized and qualified procedures.

9.2.6.1 Safety Laboratory Test (Hematology and Biochemistry)

All safety laboratory tests will be performed locally in a qualified laboratory for the scheduled visits. If abnormal and clinically significant values are observed, a test can be repeated at the investigator's discretion. Printouts of the results of any laboratory test become automatically source documentation and need to be reviewed by the investigator (review must be confirmed by entering date and initials on the printout) and then transcribed in the subject's eCRF and made available to the sponsor or CRO for data verification at each monitoring visit.
Virology tests (HIV, HepB and C) and HLAB27 test will be performed only once during a screening visit.
Safety tests will be performed at time points indicated in the schedule of events (see section 2.2).
A pregnancy test is mandatory for all female subjects of childbearing potential and must be performed at Screening, repeated before every injection, on the day before challenge and at the last study visit. Negative pregnancy test results are a prerequisite for the investigator in order to enroll the subject in the study, inject the randomized dose and administer the challenge.

9.2.6.2 Immunology assays

Shigella exposure tests
Previous exposure to *Shigella flexneri* will be assessed during pre-screening activities, conducted under the JH200 protocol, by means of a serum IgG titer to *Shigella flexneri* LPS. Method will be described in a dedicated SSP.

Serum IgA deficiency tests
Immunosuppressive illness or immunoglobulin deficiency will be assessed at Screening by means of a serum IgA test.

Immunogenicity assays
Blood samples for immunology include obtaining serum and PBMCs by appropriate collection tubes and processing methodology.
Serum and stool samples will be assayed for the vaccine induced immune-response and qualitative (responder rates) and quantitative assessments (log transformed values) will be performed according to procedures described in SSPs.
Samples will also be collected to support additional exploratory evaluations in systems biology and immune profiling (i.e. transcriptomic, SBA, avidity, flow cytometry, memory B cells, and cytokine analysis). These will be performed according to procedures described in SSPs. Stool samples will be cryopreserved for future assays.
Time points for assessment of immune-response are described in the schedule of events (section 2.2).

9.2.7 Clinical Supply

Dosing
The date and time of administration of each treatment dose must be recorded in the source documentation and transcribed into the subject's eCRF. All baseline visit-related procedures should be performed prior to injection.

Vaccine Accountability Inventory
Vaccine accountability will be performed at the time of receipt and after every injection. The pharmacist will be responsible for accounting for all vaccine supplies dispensed and will accurately complete the vaccine accountability log provided by the sponsor.

Clinical supply complaint
A clinical supply complaint is defined as any communication concerning manufacturing, packaging, labeling, blinding or distribution (including adverse storage at site depots) of a vaccine supply that describes a potential defect related to its identity, strength, quality or purity after it is released and has left the control of a LimmaTech Biologics-approved facility for distribution. Examples include but are not limited to adverse storage of vaccine product at the trial site and dosing past expiration. Site errors/issues that do not put product disposition in question should not be reported.
The investigator shall take responsibility for and shall take all steps to maintain appropriate records and ensure appropriate supply, storage, handling, distribution and usage of investigational vaccine product in accordance with the protocol and any applicable laws and regulations. This responsibility includes reporting of all clinical supply complaints to the sponsor.
Clinical supply complaints, as defined above, must be reported to the sponsor within 1 business day of first becoming aware of the issue. The vials that are the object of the complaint must be kept at a secure place, clearly separate from the other vaccine supplies to prevent confusion. Sponsor contact information and related reporting details can be found in the investigator trial files.

9.2.8 Concomitant medication

At each study visit and follow-up contacts P1 and P2, the investigator or designee should ask the subject about any medication or vaccination taken. All concomitant medications administered at any time during the study until last study visit will be recorded into the eCRF. Only concomitant medications approved by the study physician will be used during the study period. Subjects taking regular medication (i.e., birth control pills) prior to enrollment in the trial will be allowed to continue to take this medication unless it is specifically excluded as part of the inclusion/exclusion criteria for the trial. Subjects needing to take unapproved or excluded medication (as listed in the exclusion criteria) will not be eligible for enrollment or continued eligibility in the study. Any medication ordered by the study physician during the course of the trial will be documented on appropriate source documents and in the eCRF. Approved medications being taken prior to enrollment and during the course of the trial will also be documented in this manner.

9.3 Study discontinuation

9.3.1 Study halting rules

The Principal Investigator, along with the Research Monitor, will review the safety data of the injected subjects. If any of the following events occurs, administration of the investigational product or challenge will be discontinued until a thorough review of the events is undertaken by the investigator and the Research Monitor:

During the vaccination phase (from enrolment to admission pre-challenge or last study visit for subjects not challenged):
1. Any life-threatening or fatal SAE occurring after vaccine administration
2. Any SAE that are probably or definitely related to study drug, such as anaphylactic shock reaction
3. Three or more of the same or similar (Grade 3 or higher) AEs in any treatment arm, regardless of whether the subject(s) is withdrawn by the investigator
4. Accumulation of SAEs and/or severe AEs which individually do not fulfill study halting rules, but which collectively raise a safety concern in the opinion of the investigator, Research Monitor or study sponsor.

The safety/laboratory data of the study will be reviewed on an ongoing basis by the PI. In the event that an untoward trend is observed and/or there are severe unanticipated AEs, the PI will in conjunction with the Research Monitor and sponsor, determine whether the study should be suspended pending further safety review. An outline of the extent of the safety assessment conducted and justification for the decision taken will be documented in the sponsor Trial Master File and Investigator study file.

From challenge to last study visit:
1. Any life-threatening or fatal SAE occurring after the *S. flexneri* 2a 2457T challenge dose
2. Accumulation of SAEs and/or severe AEs which individually do not fulfill study halting rules, but which collectively raise a safety concern in the opinion of the investigator, Research Monitor or study sponsor.

Based on prior experience with Shigella challenge studies, it is expected that there will be volunteers with severe adverse events (such as severe diarrhea or shigellosis-related symptoms). However, subjects will remain in the inpatient facility, which is staffed 24 hours a day with medical personnel to ensure appropriate monitoring and care of those volunteers. Volunteers experiencing severe adverse events expected as a result of Shigella inoculation will not be discontinued and the study will not be put on hold as a result of those adverse events.

9.3.2 Study termination criteria

The study can be terminated at any time for any reason by LimmaTech Biologics. In case of study termination, the investigators will be informed of the procedures to be followed to ensure that adequate consideration is given to the protection of the subject’s safety.

The investigator will be responsible for informing IRBs and the sponsor will be responsible for notifying the health authorities (FDA) of the early termination of the trial.

9.3.3 Study withdrawal

9.3.3.1 Study withdrawal criteria

Each subject may withdraw consent at any time during the study without penalty. Counselling about the subject's health will be provided if he/she decides to discontinue participation in the study. Medical advice regarding what is in the best interest of the subject will be provided.
If a subject withdraws or request to be taken off treatment, the investigator will make a reasonable effort to determine the reason for the request and to complete termination procedures. Telephone calls, registered letters, and email correspondence are considered reasonable effort. For subjects leaving the study, a final study visit should be performed (about 1 month after receipt of last investigational product and including all the activities listed in V6). If a subject wishes to withdraw or be taken off treatment after receiving the challenge, they will be encouraged to remain at inpatient until two negative cultures are obtained and after completing two doses of antibiotic. All subjects withdrawn after challenge will receive antibiotics for outpatient treatment and will be educated on the importance of complying with treatment. If possible, attempts will be made to follow-up with the subjects for safety at least 1 month after receipt of the challenge inoculum.

The PI may discontinue the subject’s activity without the subject’s consent if any of these criteria are met:
• A subject fails to comply with study procedures
• A subject’s safety or health may be compromised by further participation
• It is determined to be in the subject’s best interest

A subject may be withdrawn or taken off treatment for an adverse event (AE) or serious adverse event (SAE) resulting in a safety concern, or for noncompliance with protocol requirements. When a subject withdraws or decides to take off treatment due to an AE, or is taken off treatment or withdrawn by the PI due to an AE, the CRO and the sponsor must be notified within 24 hours from becoming aware. Investigators must follow specific policy at each institution regarding the timely reporting of AEs and SAEs to the local IRB. In all cases, the PI will make a reasonable effort to complete study termination procedures.

Immunogenicity assessments will be continued for all subjects presuming no undue risk to the subjects related to specimen collection. In general, for any subject taken off treatment a final study visit should be performed. If possible, for withdrawn subjects a final study visit should be completed prior to withdrawn.
If a subject meets withdrawal conditions for a concomitant medication violation or noncompliance, this should be clearly documented.
The sponsor reserves the right to request the withdrawal of a subject due to protocol violation, or for administrative or other reasons.
Data retrieved before the withdrawal of consent will be used as study data.

Acceptable reasons for discontinuing a subject include but are not limited to:

1. Use of any investigational or non-registered product other than the study vaccine during the study period.

2. Chronic administration of immunosuppressive or other immune-modifying drugs during the study period.

3. Subject develops a general medical condition that, in the opinion of the investigator, contraindicates continuing the study.

4. Subject lost to follow-up: Unable to locate the subject despite documented attempts to notify the subject by telephone and by regular and certified letter. A subject will not be considered lost to follow-up until the last scheduled follow-up visit.

5. Subject request to terminate: The subject withdraws consent to participate in the study (the investigator should document the reason for withdrawal of consent, if possible).

6. Subject's death: If possible, an autopsy report should be obtained to document the cause of death.

7. Investigator/sponsor termination: Investigator/sponsor may withdraw subjects for serious safety reasons based on medical judgment or other serious reasons in regards to the appropriate conduction of the study. The investigator/sponsor must document the reason for subject withdrawal.

9.3.3.2 Procedure for handling subjects discontinued

In the absence of a medical contraindication or significant protocol violation, every effort will be made by the investigator to keep the subject in the study. An excessive rate of withdrawals can render the study non-interpretable. If a subject has to be withdrawn, all efforts will be made to complete and report the trial observations as thoroughly as possible. If a treated subject needs to be taken off treatment or withdrawn, all efforts shall be made to follow safety of the subject as per protocol.

Subjects withdrawn from the study prior to injection, whether or not they have been randomized, will be replaced. No follow up of these subjects will be performed. Subjects will not be replaced if the first injection has been administered before they withdraw the consent to participation.

If any of the withdrawal criteria becomes applicable during the study, the subject may, after careful consideration by the investigator, be discontinued from the study or may be excluded from analysis.

When a subject is taken off treatment from the study before the planned end of the study period, all investigations scheduled for the end of study visit (V6) should be performed about 28 days from last vaccination or challenge, if the subject agrees, otherwise at time of the subject’s withdrawal.

End of study evaluation will be completed at the time of the subject’s withdrawal.

At a minimum the Investigator will collect the following information when a subject discontinues:

- The reason the subject discontinued

- The date of the last treatment administration
- The date of the last assessment and/or contact. A follow-up contact (telephone or visit) will be arranged as appropriate
- Any AE or (Serious) Adverse events, if any
- Update con meds if possible
- Final Assessments: Every effort should be made to ensure that all procedures and evaluations scheduled for the last study visit are performed (Section 2.2)

9.3.4 Contraindications for study drug injection

The following events constitute contraindications to administration of the study drug:

- Subject withdraws consent
- Anaphylactic reaction following the first administration
- Pregnancy
- Acute illness after first administration, which in the judgment of the investigator makes a subsequent administration not advisable
- Oral temperature above 38 °C. In this case the treatment administration can be rescheduled to a later time point.
- Any temporary immunodeficiency condition or ongoing AEs that place the subject at increased risk if treated. In this case the treatment administration can be rescheduled to a later time point.

In this trial, subjects discontinued from receiving the second administration, will continue to participate in the regularly scheduled activities (last scheduled visit for subjects not challenged, about Day 56), as long as they did not withdraw consent.

10.0 SAFETY ASSESSMENTS

The sponsor has an institutional responsibility to ensure safety of the subjects. This responsibility is vested in the Principal Investigator (PI) and the Research Monitor.

The recording of AEs is an important aspect of study conduct and must be appropriately documented. It is the responsibility of the investigator to appropriately document all AEs according to the detailed guidelines set out below. The subjects will be instructed to contact the investigator immediately should they manifest any signs or symptoms they perceive as severe.
The PI is responsible for the routine evaluation of safety aspects of the study. This includes the review of all available information. In addition, the PI is responsible for ensuring that procedures and expertise are kept at the necessary standard and available to cope with medical emergencies during the study. Emergency equipment and medications must be available within the clinical unit and their use in the context of the study has to be documented in the eCRF.

A qualified physician with previous experience in vaccine biology and infectious diseases will be appointed as Research Monitor. Contact information, references and responsibilities of the Research Monitor will be included in the Research monitor charter and in the Safety Management Plan (SMP). Briefly, Research Monitor will review any SAEs that occurred during the trial and any safety data if requested by the investigator or by the sponsor. In addition the Research Monitor will provide recommendations about continuing or stopping the study when holding rules are met and review the conditions with late onset reported until last follow up call (P3).

10.1 Definition of terms

Definitions in this section are in accordance with the International Conference on Harmonisation (ICH), Guideline for Good Clinical Practice E6 (R1).

10.1.1 Adverse event

An adverse event (AE) is defined as: "Any untoward medical occurrence in a patient or clinical investigation subject administered a pharmaceutical product and which does not necessarily have a causal relationship with this treatment. An adverse event (AE) can therefore be any unfavourable and unintended sign (including an abnormal laboratory finding), symptom, or disease temporally associated with the use of a medicinal (investigational) product, whether or not related to the medicinal (investigational) product".

10.1.2 Serious adverse event

Serious Adverse Event (SAE) is any untoward medical occurrence that at any dose:

- results in death or
- is life threatening or
- results in persistent or significant disability/incapacity or
- requires in-patient hospitalization or prolongation of existing hospitalization or
- is a congenital anomaly/birth defect in the offspring of a study subject

Life-threatening in the definition of a serious adverse event refers to an event in which the subject was at risk of death at the time of event; it does not refer to an event which hypothetically might have caused death if it was more severe.

In addition, important medical events that may jeopardize the patient or may require intervention to prevent one of the other outcomes listed above should be considered as serious (i.e. intensive treatment in an emergency room or at home for allergic bronchospasm; blood dyscrasias or convulsions that do not result in hospitalization; or development of drug dependency or drug abuse).

Hospitalization for either elective surgery related to a pre-existing condition which did not increase in severity or frequency following initiation of the study, or for routine clinical procedures (including hospitalization for "social" reasons) that are not the result of an adverse event, must not be recorded as SAE. If the hospitalization arises from a pre-existing condition, or was planned prior to the first injection, it should be recorded in the Medical History form of the Case Report Form (CRF). If the planned hospitalization or procedure is executed as planned, the record in the subject's medical history is

considered complete. However, if the event/condition worsens during the trial, it must be reported as an AE.

10.1.3 Adverse drug reaction

All noxious and unintended responses to the medicinal product related to any dose should be considered adverse drug reactions (causal relationship between the medicinal product and the adverse event cannot be ruled out).

10.1.4 Unexpected adverse event or suspected adverse reaction

An adverse event or suspected adverse reaction is considered "unexpected" if it is not listed in the investigator brochure or is not listed at the specificity or severity that has been observed, or, if an investigator brochure is not required or available, is not consistent with the risk information described in the general investigational plan or elsewhere in the current application, as amended.
For example, under this definition, hepatic necrosis would be unexpected (by virtue of greater severity) if the investigator brochure referred only to elevated hepatic enzymes or hepatitis.
"Unexpected," as used in this definition, also refers to adverse events or suspected adverse reactions that are mentioned in the investigator brochure as occurring with a class of drugs or as anticipated from the pharmacological properties of the drug, but are not specifically mentioned as occurring with the particular drug under investigation.

Detailed information about the expectedness of AEs is provided in the Investigator Brochure of Flexyn2a, where the safety data obtained in the phase I trial (Sf2a-1) are summarized.

10.2 Evaluation of AE and SAE

Each subject will be monitored for the occurrence of AEs immediately after having received the investigational product. Adverse events and SAEs which occur after informed consent is obtained, but prior to first injection, will not be documented as AEs but as medical history.

Starting with enrollment and for about 28 days after the second vaccination all AEs will be recorded and causality toward vaccination assessed. However, should an AE that occurs beyond 28 days after second vaccination appear to be related to vaccination, the AE will be recorded and causality toward vaccine will be assessed.
Starting with the challenge phase and until about 28 days after challenge (last study visit), causality of all the AEs collected will be assessed toward challenge.

At each visit/assessment, all adverse events reported by the subject, spontaneously or in response to a direct question, will be evaluated by the Investigator or one of his collaborators. The questioning of subjects with regard to the possible occurrence of adverse events will be generalized such as: "How have you been feeling since your last visit?”.
The nature of each event, date and time (where appropriate) of onset, outcome, intensity and relationship to vaccination should be established. Details of any corrective

treatment and follow-up results must be recorded in the appropriate page of the eCRF, as well as in the subject's source documentation.

Anticipated day-to-day fluctuations of pre-existing conditions that do not represent a clinically significant exacerbation do not need to be considered as adverse events. Discrete episodes of chronic conditions occurring during a study period should be reported as adverse events in order to assess changes in frequency or severity.

Adverse events should be documented in terms of a medical diagnosis. When this is not possible, the adverse event should be documented in terms of signs and symptoms observed by the Investigator or reported by the subject.

Laboratory tests values which are considered clinically significant and/or study drug related will be noted in the subject's source documentation and then transferred in the AE section of the eCRF. The investigator will record his/her medical opinion of the clinical significance of each laboratory value outside of the reference range. This decision shall be based upon the nature and degree of the observed abnormality.

The investigator may choose to repeat any abnormal result ONCE, in order to rule out any laboratory error.
Clinically relevant deviations of laboratory values occurring until last study visit should be reported. Repeated evaluations are mandatory until their normalization or until the time course and reason of the underlying process can clearly be assessed. In case of doubt, the Research Monitor must be contacted.

10.2.1 Solicited adverse events

Solicited adverse events are AEs reported by the subject upon direct question and/or detected by investigator according to the table below.
During the vaccination phase solicited AEs (table 2a) are expected to occur any time within the 7 days following injection. Local solicited adverse events are usually considered related to the injection.
In addition, subjects included in this phase 2b study are expected to have, following the challenge, several symptoms including severe adverse events (such as severe diarrhea or related symptoms (table 2b).

Table 2*:*

**Solicited Local AEs** (at the injection site)	**Solicited General AEs**
Pain/Tenderness<br>Erythema / Redness<br>Induration/Swelling	Headache<br>Diarrhea (loose/watery stools)<br>Vomiting<br>Fever<br>Myalgia (specify)<br>Arthralgia (specify)<br>Body rash<br>Nausea<br>Abdominal pain<br>Chills<br>Sweats

a) Solicited AEs following vaccination

Note: List of solicited AEs selected from the FDA Guidance on Vaccines in Healthy Volunteers

**Solicited post-challenge AEs**
Diarrhea of any severity<br>Fever<br>Dysentery<br>Constitutional and/or enteric symptoms (Nausea, Vomiting, Abdominal Cramps/Pain, Myalgia, Arthralgia, Rigors, Tenesmus and Fecal urgency)<br>Headache, excessive flatulence, anorexia and malaise.<br>Rash and restlessness

b) Solicited AEs following challenge

10.2.2 Unsolicited adverse events

Unsolicited AEs are symptoms reported spontaneously by the subjects or detected by investigator any time during the trial, starting at the point of the first injection through last clinic study visit.

10.3 Assessment of intensity

Intensity of the local (at the injection site) AEs should be assessed as described in the table below.

Table 3: Classification of intensity of local AEs

**Local reaction at injected site**	**Mild (Grade 1)**	**Moderate (Grade 2)**	**Severe (Grade 3)**	**Potentially Life-Threatening (Grade 4)**
Pain/Tenderness	Does not interfere with daily activity	Repeated use of non-narcotic pain reliever > 24 hours or interferes with daily activity	Any use of narcotic pain reliever or prevents daily activity	ER visit or hospitalization
Erythema/Redness *	2.5 – 5 cm	5.1 – 10 cm	> 10 cm	Necrosis or exfoliative dermatitis
Induration/Swelling **	2.5 – 5 cm and does not interfere with daily activity	5.1 – 10 cm or interferes with daily activity	> 10 cm or prevents daily activity	Necrosis

* In addition to grading the measured local reaction at the greatest single diameter, the measurement should be recorded as a continuous variable.

** Induration/Swelling should be evaluated and graded using the functional scale as well as the actual measurement at the greatest diameter.

For the classification of intensity of fever, maximum intensity will be assigned as follows:

**Oral Temperature** [1]	**Mild (Grade 1)**	**Moderate (Grade 2)**	**Severe (Grade 3)**	**Potentially Life Threatening (Grade 4)**
Fever (°C)<br>Fever (°F)	38.0–38.4<br>100.4–101.1	38.5–38.9<br>101.2–102.0	39.0–40<br>102.1–104	> 40<br>> 104
Vomiting	1 episode in 24 hours	2 episodes in 24 hours	> 3 episodes in 24 hours	Requires hospitalization for IV fluids

[1] Oral temperature; no recent hot or cold beverages or smoking. Oral temperature will be recorded once daily, preferably in the evening. Should additional temperature measurements be performed at other times of day, the highest temperature will be recorded. If temperature is ≥ 100.4 °F every attempt should be made to repeat the temperature in about 20 minutes after the subject has rested with no eating, no drinking or smoking. If the repeat temperature is WNL and the investigator feels the repeated temperature is a more accurate reflection of the subject's real temperature the repeated value will be entered into the eCRF.

For the classification of intensity of diarrhea during the outpatient phase:

**Diarrhea**	**Mild (Grade 1)**	**Moderate (Grade 2)**	**Severe (Grade 3)**	**Potentially Life Threatening (Grade 4)**
Loose stools are stools to be determined to be grade 3 - 5 by study team.	Outpatient 3 loose stools in 24 hours	Outpatient 4-5 loose stools in 24 hours	Outpatient 6 or more loose stools in 24 hours	Requires hospitalization for IVF's

For any other general adverse event maximum intensity should be assigned to one of the following categories listed in Table 4 below.

Table 4: Assessment of severity of general AEs

Grade		
1	Mild	Does not interfere with routine activities. Minimal level of discomfort.
2	Moderate	Interferes with routine activities. Moderate level of discomfort.
3	Severe	Unable to perform routine activities. Significant level of discomfort.
4	Potentially Life Threatening	Hospitalization or ER visit for potentially life-threatening event.

Heart rate, blood pressure, hematology and clinical chemistry parameters will be classified according to the measured value (respective to its normal range) and its clinical significance. Reference ranges for adverse events are in Appendix A.

As defined by the ICH guideline for Good Clinical Practice (GCP), the term “severe” is used to describe intensity (severity) of a specific event (as in mild, moderate, or severe myocardial infarction); the event itself, however, may be of relatively minor medical significance (such as severe headache). This is not the same as “serious,” which is based on subject/event outcome or action criteria usually associated with events that pose a threat to a subject’s life or functioning. Seriousness (not severity) serves as a guide for defining regulatory reporting obligations.

10.4 Assessment of causality

Every effort should be made by investigator to explain each adverse event and assess its causal relationship, if any, to administration of study product (vaccine or placebo) or challenge.

The degree of certainty with which an adverse event can be attributed to the vaccine or challenge administration (or alternative causes, e.g. natural history of an underlying disease, concomitant therapy, etc.) will be determined by how well the event can be understood in terms of one or more of the following parameters:

- Reaction of similar nature having previously been observed with this type of vaccine and/or formulation or challenge.
- The event having often been reported in the literature for similar types of vaccines or challenges.
- The event being temporally associated with vaccination (or reproduced on re-vaccination) or challenge.

All solicited local (injection site) reactions will be considered causally related to vaccine treatment, unless there is an alternative clear explanation for the event.
The principal investigator must assess the relationship of all other adverse events (including SAEs) to the treatment (vaccine or placebo) or to the challenge answering the following question:

Did the administered treatment (vaccine or challenge) possibly contribute to the adverse event?

**NO:**	**Not related:**	No relationship to the study product or challenge. Applies to those events for which evidence exists that there is an alternate etiology.
	**Unlikely:**	Likely unrelated to the investigational product or to challenge. Likely to be related to factors other than the study product or challenge but cannot be ruled out with certainty.
**YES:**	**Possible:**	An association between the event and the administration of the investigational product or challenge cannot be ruled out. There is a reasonable temporal association, but there may also be an alternative etiology such as the subject's clinical status or underlying factors including other therapy.
	**Probable:**	There is a high degree of certainty that a relationship to the investigational product or challenge exists. There is a reasonable temporal association, and the event cannot be explained by known characteristics of the subject's clinical state or factors including other therapy.
	**Definite:**	An association exists between the receipt of the investigational product or challenge and the event. An association to other factors has been ruled out.

If an event meets the criteria to be classified as "serious", it will be examined by investigator to an extent to be able to determine all contributing factors applicable to each SAE. Other possible contributors include but are not limited to:

- Medical history
- Other medication
- Protocol required procedure

- Erroneous administration
- Other cause (specify)

10.5 Follow up of AEs and SAEs and assessment of outcome

Investigators should follow subjects with SAEs until the event has resolved, subsided, stabilized, the event is otherwise explained, or the subject is lost to follow-up within a maximum of 30 days after the last scheduled study visit or 30 days after early termination. Either during or following the assessment the investigator may refer the subject to an appropriate medical specialist.

Clinically significant laboratory abnormalities, as well as any AE, will be followed up until they have returned to normal values, or stabilized or a satisfactory explanation has been provided within a maximum of 30 days after the last scheduled study visit. If after this period the AE is neither resolved nor otherwise explained, the investigator will perform a final assessment and refers the subject to an appropriate medical specialist.

Outcome during the vaccination phase should finally be assessed as:

1=Ongoing
2=Resolved
3=Lost to follow-up
4=Death

Outcome during the challenge phase should finally be assessed as:

1=Ongoing
2=Resolved
3=Lost to follow-up
4=Death
5=Severity change (based on calendar days with the exception of diarrhea and vomiting, as clarified in the SSP)

10.6 AEs Reporting Requirements

Adverse events which occur after informed consent is obtained, but prior to injection, will not be documented as AEs but as medical history, irrespective of severity.

At each visit/assessment, all adverse events, either observed by the Investigator or one of his clinical collaborators or reported by the subject spontaneously or in response to a direct question will be evaluated by the Investigator and recorded in the AE form within the subject's CRF irrespective of severity or whether or not they are considered vaccination or challenge-related. The nature of each event, date and time (where appropriate) of onset, outcome, intensity and relationship to injection should be evaluated. Details of any related treatment should be recorded in the appropriate section of the eCRF.

A detailed procedure on AEs reporting will be given in the “Safety Management Plan” document of the SF2A-2b study.

10.7 SAEs Reporting Requirements

10.7.1 SAEs

The investigator will report any SAE whether or not considered related to the study vaccine or to challenge to the Research Monitor, CRO and sponsor by entering data in the SAE form in the AdvantageEDC system.
In the event of a death or a SAE determined by investigator to be related to vaccination or to challenge, receipt of the AdvantageEDC form submission must be confirmed by a telephone call to the Investigator.

Data about the SAE should be entered as soon as possible, but not later than **24 hours** after becoming aware of the event. All SAEs should be documented on a SAE form, along with an explanation of any medical treatment administered. The investigator should not wait to receive additional information to fully document the event before notifying the SAE. At a minimum, the initial notification should include sufficient information to allow identification of:

- the reporter
- the subject
- investigational product/challenge
- date(s) of administration
- description of the event, as much detailed as possible
- date of onset

The initial SAE report should be followed by a full summary utilizing the Serious Adverse Event pages in the individual eCRF, detailing relevant aspects of the adverse event in question. Where applicable, every effort must be made to gather information from relevant hospital case records and autopsy reports and submit into the Advantage EDC system.
Instances of death, cancer or congenital abnormality in offspring, if brought to the attention of investigator AT ANY TIME after cessation of the study, should be reported to the sponsor.
In the event that the AdvantageEDC system is down, a paper SAE Reporting Form must be filled out and sent to the CRO, sponsor and Research monitor within 24 hours of awareness of the event, via fax or e-mail.
The sponsor has a legal responsibility to promptly notify, as appropriate, the regulatory agencies about the safety of a product under clinical investigation. It is the responsibility of the investigator to inform their IEC/IRB about SAEs or other events as required by their local procedures and in compliance with the applicable regulations. In case of an SAE leading to death, the investigator will immediately inform the IRB.

A detailed procedure on SAEs reporting and recording as well as the list of contacts is given in the "Safety Management Plan" document of the SF2A-2b study.

10.7.2 Suspected Unexpected Serious Adverse Reactions (SUSARs)

When an AE is judged to be related to the study drug administered (as described in 10.4), to be serious (as described in section 10.1.2) and also to be unexpected (as

described in 10.1.4), it is defined as a SUSAR (Suspected Unexpected Serious Adverse Reaction) and is subject to expedited reporting to IEC/IRB and the FDA.

The sponsor is responsible for the evaluation of SUSARs, once the investigator has judged the SAE to be related to the investigational product and unexpected.

SUSARs are reported to the IRB by investigator and to the FDA by the sponsor. SUSARs are reported even after the trial is over, if the sponsor, Research Monitor or principal investigator becomes aware of them.
The timelines of reporting SUSARs to the IRB and the FDA are as follows:

- **within 7 days** for lethal or life-threatening SUSARs,
- **within 15 days** for other events requiring notification.

In addition to single individual SUSAR notifications, an Annual Safety Report (a summary of the current status of knowledge describing the identified and potential risks of active substances / medicinal products during clinical trials) will be submitted.

10.8 Post-study Safety Follow-Up

In order to capture SAEs with late onset (events occurring after the last clinical visit), a phone call at about Day 240 (Day 208 vaccinees only) has been scheduled. During this last follow-up call Investigators will ask about the general health status of the subject, as well as about symptoms the subjects experienced.
In particular:
- Recording of new diagnoses (e.g. reactive arthritis or IBS for challenged subjects), or chronic illnesses
- Recording of SAEs
- Recording of any vaccination

The investigator is not obliged to further actively seek AEs or SAEs in former study participants. However, if the investigator learns of any SAEs, including death, at any time after a subject has been discharged from the study, and he/she considers the events reasonably related to the treatment administered, the investigator must promptly notify the sponsor.

10.9 Pregnancy

Women who become pregnant during the study must not receive additional doses of vaccine nor be challenged, but may continue other study procedures at the discretion of the investigator.

Women should be instructed to notify the investigator, if they become pregnant during the study. Although not considered an adverse event, pregnancy must be reported on a specific Pregnancy Report Form within 24 hours (1 calendar day) of the Investigator's becoming aware of the event to the Sponsor, CRO and Research Monitor. Pregnancy Report Forms are provided in the Investigator's study file. Each pregnancy must be reported immediately (within 72 hours of identification) by e-mail or fax to the IRB.

A pregnancy should be followed to term, any premature terminations reported, and the health status of the mother and child including date of delivery and the child's gender,

height and weight should be reported after delivery. Complications and/or abnormalities should be reported.
A pregnancy is reported as an AE or SAE only when there is suspicion that the vaccine product may have interfered with the effectiveness of contraception or there was a serious complication in the pregnancy including a spontaneous abortion or an elective termination for medical rationale.

11.0 DATA ANALYSIS

Primary endpoint will be assessed with efficacy data collected at the end of the in-patients phase (after all patients have been discharged).
Secondary endpoints on safety and immunogenicity will be assessed with data collected during vaccination phase.
The Clinical Study Report will be prepared from locked data collected until last study visit (V6), and then amended to include data from the post-study safety review conducted on the 6 month follow-up phone call (D240).

11.1 Analysis populations

This study plans to investigate one single candidate vaccine dosage in one active treatment group and one placebo control group, comprised of 36 subjects each respectively
The different analysis sets to be used in data analysis are the following:

- **Efficacy population:**
    - Intent-to-treat (ITT), defined as all subjects who were randomized, received the injection of investigational product and were challenged. Subjects are analyzed according to randomized treatment rather than received treatment.
    - Per-protocol (PP), defined as those evaluable randomized, administered and challenged subjects without any major protocol deviation, as defined prior to data base lock. A major protocol deviation is defined as a deviation that is considered to have an impact on the immunogenicity and/or efficacy results of the study. The major protocol deviations (e.g., violation of inclusion/exclusion criteria, forbidden concomitant medication, unjustified early antibiotic treatment, etc.) will be identified prior to the analysis and a clinical judgment might be necessary to classify each deviation as "major" or not.
- **Safety population** defined as all subjects who were randomized and received at least one injection of investigational product. Subjects are analyzed according to treatment received rather than randomized treatment arm.
- **Immunogenicity population** defined as all subjects who were randomized and received both injections of investigational product. Subjects are analyzed according to treatment received rather than randomized treatment arm.

11.2 Statistical analysis

Detailed statistical procedures, listings, table shells, and figures will be provided in a separate statistical analysis plan (SAP) written after protocol approval. The SAP will be finalized for data analysis prior to locking the data (last patient last visit V6), and then amended to include the analysis through study Day 240. The following key statistical components will be considered, and a detailed description will be documented in the SAP:

Primary and secondary endpoints and how they will be measured

Statistical methods and tests that will be used to analyze the endpoints

Indication of whether the comparisons will be one-tailed or two-tailed (with justification of the choice) and the level of significance to be used

Identification of whether any adjustments to the significance level or the overall p value will be made to account for any planned or unplanned subgroup analyses or multiple testing

Specification of potential adjusted analyses and a statement of which covariates or factors will be included

Planned exploratory analyses

11.3 Efficacy analysis

All efficacy analyses will be performed on the ITT population. Sensitivity analyses will be these analyses repeated using the PP population.
Evaluation on the primary endpoint of the *Shigella* candidate vaccine in protecting against shigellosis will be investigated by:

- Comparing the attack rate of shigellosis after challenge between the two arms, injected with candidate-vaccine or placebo

For the additional efficacy endpoints the following will be included in the analysis and compared between the two arms, injected with candidate-vaccine or placebo:

- Number of subjects with moderate to severe diarrhea
- Number of subjects with more-severe diarrhea
- Number of subjects with diarrhea of any severity
- Mean and medians of total weight of grade 3-5 stools passed per subject
- Mean and median number of grade 3-5 stools per subject
- Number of subjects with any constitutional or enteric symptoms rated as moderate to severe
- Number of subjects who indicate they would have reduced their daily activity if they had been vacationing or traveling for business because of their Shigella illness
- Mean and median time from challenge to onset of shigellosis
- Number of cfu’s of the challenge strain per gram of stool
- Number of subjects requiring early antibiotic treatment
- Number of subjects requiring IV fluids

- Mean and median duration of diarrhea
- Number of subjects with fever
- Highest recorded febrile temperature
- Number of stools with blood

The first efficacy analysis will be conducted on the PP and ITT population and test the following hypothesis:

H0: Rcandidate vaccine ≥ Rplacebo
H1: Rcandidate vaccine < Rplacebo

Where Rplacebo and Rcandidate vaccine are the true attack rates of shigellosis for the Placebo and Candidate Vaccine arms respectively.

11.4 Safety analysis

All subjects receiving at least one treatment will be included in the safety analysis.

Adverse Events

AEs data will be listed individually and summarized by body system and preferred terms within a body system for each treatment group. Serious and/or unexpected AEs will also be discussed on a case-by-case basis. For the tabulation of AEs by body system, a subject will be counted only once in a given body system. For example, a subject reporting nausea and diarrhea will be reported as one subject, but the symptoms will be listed as two separate AEs within the class. Therefore, the total number of AEs reported within a body system may exceed the number of subjects within the body system reporting AEs.

Rates of all AEs will be analyzed by Pearson's Chi-square test (or Fisher's exact test if assumptions are not met for Pearson's Chi-square) to compare groups/cohort. Summary tables will be created that will indicate the number of subjects who experienced events. AEs will be tabulated by study group. In addition, tables will be prepared to list each AE, the number of subjects in each treatment group who experienced an event at least once, and the proportion of subjects with AE(s). AEs will be divided into defined severity grades (see section 10.3) and on the relationship to the investigational product or to challenge.

Clinical Laboratory Data Analyses

Clinical and laboratory data regarding safety, which include vital signs and safety laboratory measures, will be included in the safety analysis. Changes in pulse rate, systolic and diastolic blood pressure will be compared within each group and among groups using analysis of variance procedures. For hematology and serum chemistry tests, the mean, mean change, median, median change, and range of all values for each test for each treatment group at baseline and for the final "on therapy" values will be tabulated.

A second table (a "shift table") will be made showing for each laboratory variable the percentage of subjects in each treatment group whose values decreased, stayed the

same, or increased between the baseline or pre-treatment period and the last clinic visit. A third table will be prepared displaying the numbers of subjects in each treatment group who had values below, within, and above the normal range at baseline and at the final visit.

These tables will be reviewed by the PI and Research Monitor to evaluate whether any significant trends in laboratory values occurred.

Analysis of demographics/baseline characteristics and concomitant treatments

All baseline data, including subject demographics, medical history, and concomitant medications, will be tabulated by study arm and in total. Age and BMI of the enrolled subjects, as a whole, and per group, will be summarized.
Medications will be coded according to the WHO dictionary and summarized by ATC class and preferred term. The disposition of subjects and exposure to one or two injections will also be tabulated.

11.5 Immunogenicity analysis

All subjects who received both doses of the investigational products will be included in the immunogenicity analyses.
Standard descriptive statistics will be provided for anti-Flexyn2a IgG and IgA antibody titers (from serum and stools, respectively) as measured by ELISA prior to and after each dose, prior to and after challenge, and at study end (Day 84 or Day 56). Geometric mean titers will be calculated along with 95% confidence intervals or standard deviation. Between-group comparisons will be examined with nonparametric tests (Kruskal-Wallis for continuous data and Fisher's exact test for categorical data) unless assumptions are fulfilled for Student's t or $\chi 2$ tests.

Additional comparisons will be made using repeated measures analysis of variance with study groups as the between-subject factor and sample collection time-points as the repeated factor.

A similar statistical evaluation might be provided (as exploratory analysis) for anti-Flexyn2a mucosal response as measured by i.e. anti-2a specific PBMCs isolated prior to vaccination as well as thereafter. Measured values and differences from baseline and between the groups will be presented.

In addition, as exploratory evaluations, functionality of the vaccine-induced immune response and the level of post-challenge immune response (i.e. 2a-specific PMBCs, SBA) might be compared between the two arms injected with candidate vaccine or placebo.

A study specific procedure will be in place to define the workflow of all immunogenicity assessments.
Duplicate aliquots of all samples will be stored at -20°C or -80°C (serum and PBMCs) at the immunology laboratory and at least one aliquot will be shipped at the sponsor facility.

11.6 Protocol deviations

Protocol deviations will be listed and summarized but will not necessarily always lead to exclusion from the analysis population. More details will be presented in the Statistical Analysis Plan (SAP), which will be finalized prior to database lock.
If any of the following treatments are prescribed during the study, they need to be recorded in the eCRF:

- Use of any investigational or non-registered product, drug or vaccine other than the study vaccine or placebo formulation during the study
- Administration of a vaccine not foreseen by the study protocol during the period starting from 30 days before first administration and until last study visit
- Treatment with immunosuppressive drugs including systemic corticosteroids, which means prednisone or equivalent, ≥ 0.5 mg/kg/day for more than 1 week within 4 weeks after injection
- Use of immune-stimulators
- Administration of any blood products or immunoglobulins during the study period

11.7 Sample Size calculation

This is a phase 2b trial whose primary objective is to demonstrate the efficacy of the Flexyn2a bioconjugate vaccine.

The null hypothesis for the study is that the incidence of shigellosis in the vaccine group is the same or higher than in the placebo group; the alternative being that the incidence of shigellosis will be lower in the vaccine recipients.

For sample size estimation, based on published data from previous challenge studies performed with the same Shigella strain used for challenge in this trial, we assume a rate of diarrhea of:

- 70% in the Placebo arm
- 30% in the Vaccine arm

Based on the above assumptions and using a Chi square Test (lower bound of 95% confidence interval around point estimate of efficacy of > zero), a sample size of N= 60 evaluable subjects (30 in the Placebo arm and 30 in the Vaccine arm) will provide 80% power to demonstrate that the attack rate in the Vaccine arm tends to be smaller than that in the Placebo arm (equivalent to >57% protective efficacy). 1 alternate will be asked to come to the in-patient clinic on the first overnight in the event that a planned subject is unable to participate in the challenge planned for the following day.

To account for 10% lost-to-follow-up during vaccination phase, 6 additional subjects will be enrolled in each group, reaching a total of N= 36 randomized subjects per group or N = 72 in total.

11.8 Accounting for Missing, Unused, and Spurious Data

All missing data will be treated as missing at random and no imputation will be performed. Non-analyzable data will be documented in the deviations.

11.9 Adjudication Board

This is a double-blinded, placebo-controlled study in both the vaccination and challenge phases. In a continued effort to obtain an unbiased determination of the efficacy outcomes, an independent outcome adjudication board, the members of which will also be blinded as to the vaccination status of the challenge volunteers, will evaluate challenge outcome data following last patient last study-visit (V6).

The board will be comprised of experts on diarrheal illness case identification and pathogen diagnosis, independent of the study sponsor and investigative team. The board will also include an unblinded study statistician who will lead and coordinate the committee but will have a non-voting role in deliberations.

The board voting members will review all potential efficacy-related cases and endpoint data but will be blinded as to the vaccination status of cases.
Among the committee's responsibilities, they will:

- review and confirm all primary endpoint cases
- review all protocol-specified entry criteria, adherence, and compliance issues to ascertain classification in the per-protocol and other study populations
- provide guidance regarding secondary and other endpoint classifications to include agreement on objective criteria for classification of endpoints.

Specific duties and responsibilities will be outlined by charter prior to data lock.

11.10 Quality assurance

This study will be performed according to the ICH GCP guidelines. All specified data collected will be entered from source documents into a validated computerized clinical data management system (eCRF). Analysis of the data will only be performed after all queries have been resolved using an appropriate software for analysis. For the quality control and assurance of the study, following procedure will be kept in place:

- Feasibility study and pre-study visits for the selection of the appropriate study site

- Training of the study site personnel in study procedures

- Involvement of the Research Monitor and of an Adjudication Board for the safety and efficacy assessments

- Regular monitoring visits including:

  - Source data verification
  - Control of adherence to the protocol
  - Control of adherence to the GCP guidelines and national laws

- Site audits will be performed by the quality department of LimmaTech Biologics whenever identified necessary during the study.

12.0 ADHERENCE TO ETHICAL, REGULATORY, AND ADMINISTRATIVE CONSIDERATIONS

The trial must be conducted in accordance with GCP as outlined in the International Conference on Harmonization of Technical Requirements for Registration of Pharmaceuticals for Human Use (ICH) Guidelines, E6 Good Clinical Practice: Consolidated Guidance and other applicable laws and regulations. In addition, the trial must be conducted in accordance with the USA Code of Federal Regulations (CFR), as this trial is conducted under a USA IND, regardless of the sponsoring country involved.

12.1 Independent Ethics Committee or Institutional Review Board

Prior to initiation of the trial site, the trial, including the protocol, informed consent, and other trial documents must be approved by an appropriate Institutional Review Board (IRB) or Independent Ethics Committee (IEC). The IRB/IEC must be constituted according to applicable regulatory requirements. As appropriate, amendments to the protocol must also be approved by the IRBs/IECs before implementation at the sites, unless warranted to eliminate an immediate hazard. The IRB/IEC approval should be obtained in writing, clearly identifying the trial, the documents reviewed (including their version), and the date of the review. The trial as described in the protocol (or amendment), informed consent, and other trial documentation may be implemented only after all the necessary approvals have been obtained and the sponsor has confirmed that it is acceptable for the investigator to do so.
In the event that the IRB/IEC requires changes in the protocol, the sponsor shall be advised and must approve the changes prior to implementation. The investigator shall not modify the trial described in the protocol once finalized and after approval by the IRB/IEC without the prior written approval of sponsor.

In countries where the investigator submits the trial protocol and statement of informed consent to the IRB/IEC, the investigator or qualified designee will forward the approvals to the sponsor.

12.2 Subject information and consent

The details of the protocol must be provided in written format and discussed with each potential subject, and written informed consent must be obtained for all subjects before any trial-related procedure is performed. In obtaining informed consent, the information must be provided in language and terms understandable to the subject.
The subjects must give their written consent to participate in the trial. The signed and dated consent form itself must be retained by the investigator as part of the trial records. A copy of the signed and dated consent form must be given to the subject. The consent form must include all of the required elements of informed consent in accordance with ICH Guidelines E6 and local laws. In addition, the sponsor specifically requests that the consent form identify it as the sponsor and state that use of the candidate vaccine product is experimental and the side effects of the candidate vaccine are not completely known. The consent form must be approved by the appropriate IRB/IEC and sponsor before trial initiation at a trial site. Any subsequent changes to the approved informed consent form must be reviewed and approved by the appropriate IRB/IEC and sponsor before implementation.

12.3 Subject ID contact card

A Subject ID Contact Card is provided to each subject to carry on his or her person at all times while the subject is participating in the trial. The card must be provided to the subject as soon as investigational product is dispensed. The card is to be shown to caregivers in the event of an emergency. At a minimum, the card must contain the following information:

- Subject ID
- A statement identifying the card-carrier as a participant in a clinical trial (e.g., "This person is participating in a clinical research trial.")
- Contact information in the event of an emergency or hospitalization. The contact information on the card should indicate the investigator or designated site contact information.

As with any other information provided to subjects, the card must be approved by the IRB/IEC. The investigator will inform subjects to carry the cards with them for their whole participation in the trial.
Investigator/site personnel should collect the cards at their last in-person contact with the subject and retain them along with other clinical trial documents. If a subject loses the card or is unable to return the card at the final visit, it will not be considered a protocol violation.

12.4 Compensation for Participation

Volunteers will be compensated for their time and effort in this study. Compensation for participation will occur as detailed below. Compensation will be provided only for completed study procedures designated for compensatory payment. If a Subject is eligible to participate in the investigational protocol after screening, and s/he completes all study visits, procedures and follows all the rules s/he will receive the following compensation:

$80 total for screening (only if enrolled in the study or presents as an alternate)
$150 for each vaccination day
$80 for other outpatient visit day
$50 for each phone call following vaccination
$2,000 for the inpatient challenge phase
$80 for the end of study phone call
$600 bonus for completing the entire study, including vaccination and challenge

If a volunteer is not eligible for discharge on day 8 because of illness or not having 2 consecutive negative stool culture results s/he will receive $200 per additional inpatient day. Volunteers will not be paid for missed outpatient visits, and may forfeit some or all of the bonus as a result of missed visits or non-compliance.

If a volunteer agrees to return for the late blood draws and consents to the current protocol, they will receive:

$80 for the initial visit
$120 if they are eligible and return for the second blood draw.

Maximum compensation is $3,600 for participating in the entire trial and following all of the rules.

If a volunteer participates in the vaccination phase and does not continue to challenge, s/he will receive the following compensation:
$80 total for screening (only if enrolled in the study or presents as an alternate)
$150 for each vaccination day
$80 for other outpatient visit day
$50 for each phone call following vaccination
$80 for the end of study phone call
$300 bonus for completing the entire study, including vaccination and challenge.

Maximum compensation is $1,300 if the volunteer participates in the vaccination phase but does not proceed to challenge, but is willing to come back for the long-term follow-up visit (V7).

Alternate volunteers will be invited to the first vaccination and to admission to the unit to act as replacements in the instance that one of the scheduled participants does not show or is no longer eligible. If a volunteer is an alternate for a vaccination day and is not chosen, s/he will receive $80 as well as $80 for screening. If an alternate is admitted to the unit but not chosen for challenge, s/he will receive $200 for each day they are on the unit.

12.5 Registration of the trial

The trial will be registered by the sponsor on a publicly accessible database.

12.6 Data collection forms

The sponsor will provide the site with data collection forms, in the form of electronic Case Report Forms (eCRF), diaries, Electronic Data Capture (EDC) screens, or other appropriate data collection forms as the trial requires. The investigator is to provide subject data according to the sponsor’s instructions, in the designated data collection form, compliant with GCP practices. All completed data collection forms and the databases from the trial are the exclusive property of the sponsor.
The investigator must maintain records and data during the trial in compliance with all applicable legal and regulatory requirements. Each data point must be supported by a source document at the trial site. Any records or documents used as the source of information (called the “subject source data”) are to be retained for review by authorized representatives of the sponsor or a regulatory agency.

The investigator will ensure that there are sufficient time, staff, and facilities available for the duration of the trial to conduct and record the trial as described in the protocol and according to all applicable guidelines, laws, and regulations. All dates appearing on the sponsor's subject data collection forms for laboratory tests, examinations, and other data collected, must be the dates on which the specimens were obtained, or the procedures performed.

12.7 Electronic Case Report Forms for randomized subjects

An eCRF must be completed for all subjects who have been randomized and have received study treatment. The sponsor must not collect subject names, initials, or other personal identifiable information from any subject that is beyond the scope of the trial. Subjects are not to be identified by name or initials on the eCRF. The only acceptable identification for a subject who may appear on a CRF is the unique subject ID number. The investigator must maintain contact information for each participant so that all can be quickly contacted by the investigator, if necessary.
All entries into eCRFs are the responsibility of the investigator and must be completed by the investigator or a qualified designee. After all data entered into eCRF have been completed and all queries resolved, prior to database lock the Principal Investigator will attest in writing that he has verified the accuracy of the recorded data by signing an Investigator eCRF Sign-off Documentation form.

12.8 Data collection for screening failure subjects

Data are to be collected from the time the informed consent form is signed until the subject is determined to have failed screening. In case of screening failure the corresponding data will be recorded in the source data only. Demographic data may be entered in the eCRF, but other subject data will be entered into the eCRF, only if the subject is eligible and has been randomized.

13.0 ADMINISTRATIVE MATTERS

To comply with Good Clinical Practice important administrative obligations relating to the investigator's responsibilities, monitoring, archiving data, audits, confidentiality and publications must be fulfilled. For additional details, please refer to the Clinical Trial Agreement between Sponsor and study center.

13.1 Sponsor

The sponsor of this trial is indicated in Section 1.0, Title Page.

13.2 Investigator

The Principal Investigator for this trial has been indicated in Section 1.0.

Responsibility of the investigator
The investigator is responsible for the following:

- To ensure that he/she has enough time to dedicate to the conduct of the study in compliance with the protocol, GCP and regulatory requirements
- To prepare and maintain adequate subject source data or raw data designed to record observations and any data pertinent to the study.
- To cooperate with the study monitor in the monitoring process of the study and in the timely resolution of queries about the data
- To follow the IRB/IEC and FDA requirements
- To submit any SAE to the Research Monitor, CRO and Sponsor of the study within 24 hours
- To submit and update his/her curriculum vitae
- To maintain all information provided by the sponsor and all data generated by the site as part of the study strictly confidential. This information and data will not be

used by investigator or other site personnel for any purpose other than conducting the study.
- For all additional investigators' responsibilities, publications policy and financial aspects please refer to clinical trial agreement.

Financial disclosure
In relation to the clinical trial described in this protocol, the investigator certifies to have read and answered a Financial Disclosure Form or equivalent document truthfully and to the best of his knowledge. The Principal Investigator is also responsible to certify that sub-investigators have read and answered the Financial Disclosure Form as a condition of their participation in the trial.
If any financial interest reported on the Financial Disclosure Form changes during the course of the trial or within 1 year after the last subject has completed the trial as specified in the protocol, the Principal Investigator and sub-investigators are obligated to inform the sponsor about the change.

13.3 Clinical study report
A Clinical Study Report (CSR) will be prepared by the sponsor or its qualified designee to describe the results of the trial. The CSR will describe the study results based on the data collected through the last follow-up phone call (D240).

13.4 Clinical research organization
To support the sponsor in conducting the trial some tasks will be delegated to a CRO. The delegated tasks are clearly defined in a Task Delegation Form in written agreement with the contracted CRO.

13.5 Monitoring
The monitoring activities at the investigational site will be delegated to a CRO. Study monitors will contact and visit the clinical site regularly and will be allowed, on request, to inspect the various records of the study, provided that subject confidentiality is maintained in accord with local requirements. During the Study Initiation Visit (SIV) a representative of the CRO will visit the clinical site to:

- Determine the adequacy of the facility
- Discuss with investigator(s) and other personnel involved with the study their responsibilities with regard to protocol adherence, and the responsibilities of sponsor or its representatives.

During the study, the monitor or another representative of the sponsor will establish regular contacts with the investigational site, with the intent to:

- Provide information and support to investigator(s).
- Confirm that facilities and equipment remain adequate for the conduct of the study.
- Confirm that the investigational team is conducting the study in adherence to the protocol, data are being accurately and timely recorded in the eCRFs, and vaccine accountability logs are kept up to date.
- Confirm that relevant GCP guidelines and subject's rights are respected.

- Perform source data review.
- Perform source data verification. This will require direct access to all original records for each subject (e.g. clinic charts, laboratory test reports, correspondence from general practitioners, etc.). A detailed description of source data will be given in the monitoring plan.

The monitor or another representative of the sponsor will be available between visits if investigator(s) or other staff at the site needs information and advice. The investigator shall agree to co-operate with the study monitor to ensure that any issue detected in the course of these monitoring visits are resolved in a timely manner. Direct access to all study-related site and source data/documents is mandatory for the purpose of the monitoring review. The Principal Investigator must ensure provision of reasonable time, space and adequate qualified personnel during the monitoring visits.

13.6 Quality assurance audit

Independent auditors designated by the sponsor may conduct a systematic examination of study related activities, documents and site to assess whether the evaluated study activities were conducted, and data were recorded, analyzed and accurately reported according to approved protocol, standard operating procedures, current Good Clinical Practice, and the applicable regulatory requirements. Audit observations and findings will be documented and communicated to appropriate study personnel and management. A corrective and preventive action plan will be requested and documented in response to any audit observations.

13.7 Access to original study data

Members of the Institutional Review Board or Independent Ethic Committee, sponsor representatives, as well as the FDA have the right to access the site and all original study-related source data or documents pertaining to the conduct of this study.

13.8 Data collection and record retention

For the purpose of monitoring and auditing the study, source documentation may consist of existing medical records and/or study records developed and maintained by the investigator or provided by the sponsor as agreed with investigator.
Data recorded on source documents will be transcribed onto eCRF provided by the CRO. A copy of each completed eCRF will be retained at the clinical site as part of the study records.
The study will be monitored regularly by the CRO. All study records (source documents, signed informed consent forms, copies of CRFs, IRB/IEC correspondence and approval letters, candidate vaccine management records, etc.) will be kept secured for the time period described in section 13.12. Investigator will ensure that study records are not disposed of or removed from the clinical site without prior notification and approval from the sponsor.

13.9 Insurance for candidate vaccine-induced injury

The sponsor assumes liability for and will indemnify all injuries that occur to trial subjects whenever a causal relationship can be established between the event and the clinical trial procedure or the candidate vaccine under study if the following can be demonstrated:

- The event resulted from the candidate vaccine, provided that the substance was administered according to the approved trial protocol.
- The event occurred as a consequence of non-routine / study-specific diagnostic procedures performed according to the trial protocol.
- The event resulted from therapeutic or diagnostic measures legitimately required as a consequence of unexpected events caused by the candidate vaccine or by diagnostic procedures called for by the trial protocol.

The sponsor is not liable for events that occur solely as a consequence of any underlying illness of the subject or for events resulting from diagnostic or therapeutic measures not specifically required by the protocol, or for events resulting from negligence (including failure to act according to accepted medical practice, or to comply strictly with the protocol or the terms of this Agreement) of investigators or any other involved and/or related clinical staff and facilities.

This indemnity provided by the sponsor shall further apply as follows:

- The sponsor is to be informed as soon as possible of any complaint, action or suit of proceeding giving rise to the right of indemnification, and investigators agree to cooperate fully with the sponsor in the defense or disposition of all such cases.
- The sponsor will be permitted, at its costs and discretion, to handle and control the defense or disposition of all such cases.

No case will be settled without the prior written consent of the sponsor.

13.10 **Early trial termination**

It is the intention of all that this trial is carried out to its conclusion, but investigators must be aware that for a number of reasons, the trial may need to be stopped prior to the official end date. The sponsor, therefore, reserves the right to terminate this Agreement:

- Upon a substantial breach of the terms either of the mutual Agreement or the conduct of the protocol.
- In the event of irregularities in the method by which the study is carried out and although capable of being rectified, are not rectified within thirty (30) days of notice from the sponsor requiring this.
- If this is necessary in the interests of health and safety of the study subjects, or as a result of an order of any government authority or court of law.
- Based on any internal decision.

In the event of early termination, investigators will cease use of the candidate vaccine immediately and perform a final evaluation of the subjects' safety. In case of any active SAE, these will be followed up until the outcome is a) resolved, b) death, c) lost to follow-up, within a maximum of 30 days after early termination. If after this 30 day-period the outcome is neither resolved, death, lost to follow-up, the investigator will perform a final assessment and refers the subject to an appropriate medical specialist. All CRFs outstanding must be completed and returned to the sponsor together with completed study drug inventory, records and remaining trial material.

13.11 Publications and other rights

In accord with standard editorial and ethical practice, the Sponsor will support publication of this trial. All rights and duties related to publication are regulated as described in the Clinical Trial Agreement with the clinical site.

13.12 Trial documents and records retention

During the trial and after termination of the trial – including after early termination of the trial – the investigator must maintain copies of all documents and records relating to the conduct of the trial. This documentation includes, but is not limited to, protocols, CRFs and other data collection forms, advertising for subject participation, AE reports, subject source data, correspondence with the FDA and IRBs/IECs, consent forms, investigator's curricula vitae/biosketch, monitor visit logs, laboratory reference ranges, and laboratory certification or quality control procedures and laboratory director curriculum vitae. Subject files and other source data must be kept for the maximum period of time permitted by the hospital, institution or private practice, or as specified below. The sponsor must be consulted, if the investigator wishes to assign the files to someone else, remove them to another location, or is unable to retain them for the specified period.

The investigator must retain trial records for the time specified by applicable laws and regulations. At a minimum, trial records must be retained for the amount of time specified by ICH Guidelines, the EU Good Clinical Practices Directive, or applicable local laws, whichever is longer:

- The ICH Guidelines specify that records must be retained for a minimum of 2 years after a marketing application for the indication is approved (or not approved) or 2 years after notifying the appropriate regulatory agency that an investigation is discontinued.
- The European Union (EU) Commission Directive 2003/63/EC which requires that Essential Documents (including Case Report Forms) other than subjects' medical files, are retained for at least fifteen (15) years after completion or discontinuation of the trial, as defined in the protocol.

All trial documents shall be made available if required by the FDA. The investigator should consult with the sponsor prior to discarding trial and/or subject files.

The sponsor will retain all sponsor-required documentation pertaining to the trial for the lifetime of the investigational product. Archived data may be held on microfiche or electronic record, provided that a back-up exists and that a paper copy can be obtained from it, if required.

14.0 REFERENCES

1. **Abu-Elyazeed, R. R., T. F. Wierzba, R. W. Frenck, S. D. Putnam, M. R. Rao, S. J. Savarino, K. A. Kamal, Peruski, L. F., Jr., Abd-El Messih, I. A., S. A. El-Alkamy, A. B. Naficy, and J. D. Clemens.** 2004. Epidemiology of Shigella-associated diarrhea in rural Egyptian children. Am J Trop Med Hyg **71**:367–372.
2. **Adams, M. M., G. E. Allison, and N. K. Verma.** 2001. Type IV O antigen modification genes in the genome of Shigella flexneri NCTC 8296. Microbiology (Reading, Engl.) **147**:851–860.
3. **Ahmad, H., and E. K. Chapnick.** 1999. Conjugated polysaccharide vaccines. Infectious disease clinics of North America **13**:113-33, vii.
4. **Ahmed, S. F., M. S. Riddle, T. F. Wierzba, I. A. Messih, M. R. Monteville, J. W. Sanders, and J. D. Klena.** 2006. Epidemiology and genetic characterization of Shigella flexneri strains isolated from three paediatric populations in Egypt (2000-2004). Epidemiol Infect **134**:1237–1248.
5. **Bercion, R., M. Demartin, C. Recio, P.-M. Massamba, T. Frank, J. M. Escribà, F. Grimont, Grimont, Patrick A D, and F.-X. Weill.** 2006. Molecular epidemiology of multidrug-resistant Shigella dysenteriae type 1 causing dysentery outbreaks in Central African Republic, 2003-2004. Trans. R. Soc. Trop. Med. Hyg. **100**:1151–1158.
6. **Cohen, D., S. Ashkenazi, M. Green, Y. Lerman, R. Slepon, G. Robin, N. Orr, D. N. Taylor, J. C. Sadoff, C. Chu, J. Shiloach, R. Schneerson, and J. B. Robbins.** 1996. Safety and immunogenicity of investigational Shigella conjugate vaccines in Israeli volunteers. Infection and immunity **64**:4074–4077.
7. **Cohen, D., S. Ashkenazi, M. S. Green, M. Gdalevich, G. Robin, R. Slepon, M. Yavzori, N. Orr, C. Block, I. Ashkenazi, J. Shemer, D. N. Taylor, T. L. Hale, J. C. Sadoff, D. Pavliakova, R. Schneerson, and J. B. Robbins.** 1997. Double-blind vaccine-controlled randomised efficacy trial of an investigational Shigella sonnei conjugate vaccine in young adults. Lancet **349**:155–159.
8. **Cohen, D., R. Bassal, S. Goren, T. Rouach, D. Taran, B. Schemberg, N. Peled, Y. Keness, S. Ken-Dror, V. Vasilev, I. Nissan, V. Agmon, and T. Shohat.** 2014. Recent trends in the epidemiology of shigellosis in Israel. Epidemiol. Infect.:1–12.
9. **Formal, S. B., E. V. Oaks, R. E. Olsen, M. Wingfield-Eggleston, P. J. Snoy, and J. P. Cogan.** 1991. Effect of prior infection with virulent Shigella flexneri 2a on the resistance of monkeys to subsequent infection with Shigella sonnei. J. Infect. Dis. **164**:533–537.
10. **Fries, L. F., A. D. Montemarano, C. P. Mallett, D. N. Taylor, T. L. Hale, and G. H. Lowell.** 2001. Safety and immunogenicity of a proteosome-Shigella flexneri 2a lipopolysaccharide vaccine administered intranasally to healthy adults. Infection and immunity **69**:4545–4553.
11. **Gardner, P., L. K. Pickering, W. A. Orenstein, A. A. Gershon, and K. L. Nichol.** 2002. Guidelines for quality standards for immunization. Clin Infect Dis **35**:503–511.
12. **Hatz, Christoph F R, B. Bally, S. Rohrer, R. Steffen, S. Kramme, C.-A. Siegrist, M. Wacker, C. Alaimo, and V. G. Fonck.** 2015. Safety and immunogenicity of a candidate bioconjugate vaccine against Shigella dysenteriae type 1 administered to healthy adults: A single blind, partially randomized Phase I study. Vaccine **33**:4594–4601.

13. **Jones, F. R., J. L. Sanchez, R. Meza, T. M. Batsel, R. Burga, E. Canal, K. Block, J. Perez, C. T. Bautista, J. Escobedo, and S. E. Walz.** 2004. Short report. High incidence of shigellosis among Peruvian soldiers deployed in the Amazon River basin. Am J Trop Med Hyg **70**:663–665.
14. **Levine, M. M., K. L. Kotloff, E. M. Barry, M. F. Pasetti, and M. B. Sztein.** 2007. Clinical trials of Shigella vaccines. two steps forward and one step back on a long, hard road. Nature reviews **5**:540–553.
15. **Lindsay, B., J. B. Ochieng, U. N. Ikumapayi, A. Toure, D. Ahmed, S. Li, S. Panchalingam, M. M. Levine, K. Kotloff, D. A. Rasko, C. R. Morris, J. Juma, B. S. Fields, M. Dione, D. Malle, S. M. Becker, E. R. Houpt, J. P. Nataro, H. Sommerfelt, M. Pop, J. Oundo, M. Antonio, A. Hossain, B. Tamboura, and O. C. Stine.** 2013. Quantitative PCR for Detection of Shigella Improves Ascertainment of Shigella Burden in Children with Moderate-to-Severe Diarrhea in Low-Income Countries. Journal of Clinical Microbiology **51**:1740–1746.
16. **Lu, C. L., C. C. Hung, Y. C. Chuang, W. C. Liu, C. T. Su, Y. C. Su, S. F. Chang, S. Y. Chang, and S. C. Chang.** Jan 4. Serologic response to primary vaccination with 7-valent pneumococcal conjugate vaccine is better than with 23-valent pneumococcal polysaccharide vaccine in HIV-infected patients in the era of combination antiretroviral therapy. Hum Vaccin Immunother **9**.
17. **McKenzie, R., R. I. Walker, G. S. Nabors, Van De Verg, L. L., C. Carpenter, G. Gomes, E. Forbes, J. H. Tian, H. H. Yang, J. L. Pace, W. J. Jackson, and A. L. Bourgeois.** 2006. Safety and immunogenicity of an oral, inactivated, whole-cell vaccine for Shigella sonnei. preclinical studies and a Phase I trial. Vaccine **24**:3735–3745.
18. **Murray, Christopher J L, T. Vos, R. Lozano, M. Naghavi, A. D. Flaxman, C. Michaud, M. Ezzati, K. Shibuya, J. A. Salomon, S. Abdalla, V. Aboyans, J. Abraham, I. Ackerman, R. Aggarwal, S. Y. Ahn, M. K. Ali, M. A. AlMazroa, M. Alvarado, H. R. Anderson, L. M. Anderson, K. G. Andrews, C. Atkinson, L. M. Baddour, A. N. Bahalim, S. Barker-Collo, L. H. Barrero, D. H. Bartels, M.-G. Basáñez, A. Baxter, M. L. Bell, E. J. Benjamin, D. Bennett, E. Bernabé, K. Bhalla, B. Bhandari, B. Bikbov, A. B. Abdulhak, G. Birbeck, J. A. Black, H. Blencowe, J. D. Blore, F. Blyth, I. Bolliger, A. Bonaventure, S. Boufous, R. Bourne, M. Boussinesq, T. Braithwaite, C. Brayne, L. Bridgett, S. Brooker, P. Brooks, T. S. Brugha, C. Bryan-Hancock, C. Bucello, R. Buchbinder, G. Buckle, C. M. Budke, M. Burch, P. Burney, R. Burstein, B. Calabria, B. Campbell, C. E. Canter, H. Carabin, J. Carapetis, L. Carmona, C. Cella, F. Charlson, H. Chen, A. T.-A. Cheng, D. Chou, S. S. Chugh, L. E. Coffeng, S. D. Colan, S. Colquhoun, K. E. Colson, J. Condon, M. D. Connor, L. T. Cooper, M. Corriere, M. Cortinovis, de Vaccaro, Karen Courville, W. Couser, B. C. Cowie, M. H. Criqui, M. Cross, K. C. Dabhadkar, M. Dahiya, N. Dahodwala, J. Damsere-Derry, G. Danaei, A. Davis, D. de Leo, L. Degenhardt, R. Dellavalle, A. Delossantos, J. Denenberg, S. Derrett, Des Jarlais, Don C, S. D. Dharmaratne, M. Dherani, C. Diaz-Torne, H. Dolk, E. R. Dorsey, T. Driscoll, H. Duber, B. Ebel, K. Edmond, A. Elbaz, S. E. Ali, H. Erskine, P. J. Erwin, P. Espindola, S. E. Ewoigbokhan, F. Farzadfar, V. Feigin, D. T. Felson, A. Ferrari, C. P. Ferri, E. M. Fèvre, M. M. Finucane, S. Flaxman, L. Flood, K. Foreman, M. H. Forouzanfar, Fowkes, Francis Gerry R, M. Fransen, M. K. Freeman, B. J. Gabbe, S. E. Gabriel, E. Gakidou, H. A. Ganatra, B. Garcia, F. Gaspari, R. F. Gillum, G. Gmel, D. Gonzalez-Medina, R. Gosselin, R. Grainger, B. Grant, J.**

Groeger, F. Guillemin, D. Gunnell, R. Gupta, J. Haagsma, H. Hagan, Y. A. Halasa, W. Hall, D. Haring, J. M. Haro, J. E. Harrison, R. Havmoeller, R. J. Hay, H. Higashi, C. Hill, B. Hoen, H. Hoffman, P. J. Hotez, D. Hoy, J. J. Huang, S. E. Ibeanusi, K. H. Jacobsen, S. L. James, D. Jarvis, R. Jasrasaria, S. Jayaraman, N. Johns, J. B. Jonas, G. Karthikeyan, N. Kassebaum, N. Kawakami, A. Keren, J.-P. Khoo, C. H. King, L. M. Knowlton, O. Kobusingye, A. Koranteng, R. Krishnamurthi, F. Laden, R. Lalloo, L. L. Laslett, T. Lathlean, J. L. Leasher, Y. Y. Lee, J. Leigh, D. Levinson, S. S. Lim, E. Limb, J. K. Lin, M. Lipnick, S. E. Lipshultz, W. Liu, M. Loane, S. L. Ohno, R. Lyons, J. Mabweijano, M. F. MacIntyre, R. Malekzadeh, L. Mallinger, S. Manivannan, W. Marcenes, L. March, D. J. Margolis, G. B. Marks, R. Marks, A. Matsumori, R. Matzopoulos, B. M. Mayosi, J. H. McAnulty, M. M. McDermott, N. McGill, J. McGrath, M. E. Medina-Mora, M. Meltzer, Z. A. Memish, G. A. Mensah, T. R. Merriman, A.-C. Meyer, V. Miglioli, M. Miller, T. R. Miller, P. B. Mitchell, C. Mock, A. O. Mocumbi, T. E. Moffitt, A. A. Mokdad, L. Monasta, M. Montico, M. Moradi-Lakeh, A. Moran, L. Morawska, R. Mori, M. E. Murdoch, M. K. Mwaniki, K. Naidoo, M. N. Nair, L. Naldi, Narayan, K M Venkat, P. K. Nelson, R. G. Nelson, M. C. Nevitt, C. R. Newton, S. Nolte, P. Norman, R. Norman, M. O'Donnell, S. O'Hanlon, C. Olives, S. B. Omer, K. Ortblad, R. Osborne, D. Ozgediz, A. Page, B. Pahari, J. D. Pandian, A. P. Rivero, S. B. Patten, N. Pearce, R. P. Padilla, F. Perez-Ruiz, N. Perico, K. Pesudovs, D. Phillips, M. R. Phillips, K. Pierce, S. Pion, G. V. Polanczyk, S. Polinder, C. A. Pope, S. Popova, E. Porrini, F. Pourmalek, M. Prince, R. L. Pullan, K. D. Ramaiah, D. Ranganathan, H. Razavi, M. Regan, J. T. Rehm, D. B. Rein, G. Remuzzi, K. Richardson, F. P. Rivara, T. Roberts, C. Robinson, De Leòn, Felipe Rodriguez, L. Ronfani, R. Room, L. C. Rosenfeld, L. Rushton, R. L. Sacco, S. Saha, U. Sampson, L. Sanchez-Riera, E. Sanman, D. C. Schwebel, J. G. Scott, M. Segui-Gomez, S. Shahraz, D. S. Shepard, H. Shin, R. Shivakoti, D. Silberberg, D. Singh, G. M. Singh, J. A. Singh, J. Singleton, D. A. Sleet, K. Sliwa, E. Smith, J. L. Smith, N. J. C. Stapelberg, A. Steer, T. Steiner, W. A. Stolk, L. J. Stovner, C. Sudfeld, S. Syed, G. Tamburlini, M. Tavakkoli, H. R. Taylor, J. A. Taylor, W. J. Taylor, B. Thomas, W. M. Thomson, G. D. Thurston, I. M. Tleyjeh, M. Tonelli, J. A. Towbin, T. Truelsen, M. K. Tsilimbaris, C. Ubeda, E. A. Undurraga, van der Werf, Marieke J, J. van Os, M. S. Vavilala, N. Venketasubramanian, M. Wang, W. Wang, K. Watt, D. J. Weatherall, M. A. Weinstock, R. Weintraub, M. G. Weisskopf, M. M. Weissman, R. A. White, H. Whiteford, N. Wiebe, S. T. Wiersma, J. D. Wilkinson, H. C. Williams, S. R. M. Williams, E. Witt, F. Wolfe, A. D. Woolf, S. Wulf, P.-H. Yeh, A. K. M. Zaidi, Z.-J. Zheng, D. Zonies, and A. D. Lopez. 2012. Disability-adjusted life years (DALYs) for 291 diseases and injuries in 21 regions, 1990–2010: a systematic analysis for the Global Burden of Disease Study 2010. The Lancet **380**:2197–2223.

19. **Osorio, M., M. D. Bray, and R. I. Walker.** 2007. Vaccine potential for inactivated shigellae. Vaccine **25**:1581–1592.
20. **Passwell, J. H., S. Ashkenazi, E. Harlev, D. Miron, R. Ramon, N. Farzam, L. Lerner-Geva, Y. Levi, C. Chu, J. Shiloach, J. B. Robbins, and R. Schneerson.** 2003. Safety and immunogenicity of Shigella sonnei-CRM9 and Shigella flexneri type 2a-rEPAsucc conjugate vaccines in one- to four-year-old children. Pediatr Infect Dis J. **22**:701–706.

21. **Passwell, J. H., S. Ashkenzi, Y. Banet-Levi, R. Ramon-Saraf, N. Farzam, L. Lerner-Geva, H. Even-Nir, B. Yerushalmi, C. Chu, J. Shiloach, J. B. Robbins, and R. Schneerson.** 2009. Age-related efficacy of Shigella O-specific polysaccharide conjugates in 1-4-year-old Israeli children. Vaccine **28**:2231–2235.
22. **Pazhani, G. P., B. Sarkar, T. Ramamurthy, S. K. Bhattacharya, Y. Takeda, and S. K. Niyogi.** 2004. Clonal multidrug-resistant Shigella dysenteriae type 1 strains associated with epidemic and sporadic dysenteries in eastern India. Antimicrob. Agents Chemother. **48**:681–684.
23. **Porter, C. K., N. Thura, R. T. Ranallo, and M. S. Riddle.** 2013. The Shigella human challenge model. Epidemiol Infect:223–232.
24. **Rahman, K. M., S. E. Arifeen, K. Zaman, M. Rahman, R. Raqib, M. Yunus, N. Begum, M. S. Islam, B. M. Sohel, M. Rahman, M. Venkatesan, T. L. Hale, D. W. Isenbarger, P. J. Sansonetti, R. E. Black, and A. H. Baqui.** 2011. Safety, dose, immunogenicity, and transmissibility of an oral live attenuated Shigella flexneri 2a vaccine candidate (SC602) among healthy adults and school children in Matlab, Bangladesh. Vaccine **29**:1347–1354.
25. **Riddle, M. S., R. W. Kaminski, C. Williams, C. Porter, S. Baqar, A. Kordis, T. Gilliland, J. Lapa, M. Coughlin, C. Soltis, E. Jones, J. Saunders, P. B. Keiser, R. T. Ranallo, R. Gormley, M. Nelson, K. R. Turbyfill, D. Tribble, and E. V. Oaks.** 2011. Safety and immunogenicity of an intranasal Shigella flexneri 2a Invaplex 50 vaccine. Vaccine **29**:7009–7019.
26. **Robbins, J. B., C. Chu, and R. Schneerson.** 1992. Hypothesis for vaccine development: Protective immunity to enteric diseases caused by nontyphoidal salmonellae and shigellae may be conferred by serum IgG antibodies to the O-specific polysaccharide of their lipopolysaccharides. Clin Infect Dis **15**:346–361.
27. **Shears, P.** 1996. Shigella infections. Apr;90(2):105-14. Ann Trop Med Parasitol **90**:105–114.
28. **Venkatesan, M., and R. T. Ranallo.** 2006. Live-attenuated Shigella vaccines. Expert Rev Vaccines **5**:669–686.
29. **Vos, T., A. D. Flaxman, M. Naghavi, R. Lozano, C. Michaud, M. Ezzati, K. Shibuya, J. A. Salomon, S. Abdalla, V. Aboyans, J. Abraham, I. Ackerman, R. Aggarwal, S. Y. Ahn, M. K. Ali, M. A. AlMazroa, M. Alvarado, H. R. Anderson, L. M. Anderson, K. G. Andrews, C. Atkinson, L. M. Baddour, A. N. Bahalim, S. Barker-Collo, L. H. Barrero, D. H. Bartels, M.-G. Basáñez, A. Baxter, M. L. Bell, E. J. Benjamin, D. Bennett, E. Bernabé, K. Bhalla, B. Bhandari, B. Bikbov, A. B. Abdulhak, G. Birbeck, J. A. Black, H. Blencowe, J. D. Blore, F. Blyth, I. Bolliger, A. Bonaventure, S. Boufous, R. Bourne, M. Boussinesq, T. Braithwaite, C. Brayne, L. Bridgett, S. Brooker, P. Brooks, T. S. Brugha, C. Bryan-Hancock, C. Bucello, R. Buchbinder, G. Buckle, C. M. Budke, M. Burch, P. Burney, R. Burstein, B. Calabria, B. Campbell, C. E. Canter, H. Carabin, J. Carapetis, L. Carmona, C. Cella, F. Charlson, H. Chen, A. T.-A. Cheng, D. Chou, S. S. Chugh, L. E. Coffeng, S. D. Colan, S. Colquhoun, K. E. Colson, J. Condon, M. D. Connor, L. T. Cooper, M. Corriere, M. Cortinovis, de Vaccaro, Karen Courville, W. Couser, B. C. Cowie, M. H. Criqui, M. Cross, K. C. Dabhadkar, M. Dahiya, N. Dahodwala, J. Damsere-Derry, G. Danaei, A. Davis, D. de Leo, L. Degenhardt, R. Dellavalle, A. Delossantos, J. Denenberg, S. Derrett, Des Jarlais, Don C, S. D. Dharmaratne, M. Dherani, C. Diaz-Torne, H. Dolk, E. R. Dorsey, T. Driscoll, H. Duber, B. Ebel, K. Edmond, A. Elbaz, S. E. Ali, H. Erskine, P. J. Erwin, P. Espindola, S. E. Ewoigbokhan, F. Farzadfar, V.**

Feigin, D. T. Felson, A. Ferrari, C. P. Ferri, E. M. Fèvre, M. M. Finucane, S. Flaxman, L. Flood, K. Foreman, M. H. Forouzanfar, Fowkes, Francis Gerry R, R. Franklin, M. Fransen, M. K. Freeman, B. J. Gabbe, S. E. Gabriel, E. Gakidou, H. A. Ganatra, B. Garcia, F. Gaspari, R. F. Gillum, G. Gmel, R. Gosselin, R. Grainger, J. Groeger, F. Guillemin, D. Gunnell, R. Gupta, J. Haagsma, H. Hagan, Y. A. Halasa, W. Hall, D. Haring, J. M. Haro, J. E. Harrison, R. Havmoeller, R. J. Hay, H. Higashi, C. Hill, B. Hoen, H. Hoffman, P. J. Hotez, D. Hoy, J. J. Huang, S. E. Ibeanusi, K. H. Jacobsen, S. L. James, D. Jarvis, R. Jasrasaria, S. Jayaraman, N. Johns, J. B. Jonas, G. Karthikeyan, N. Kassebaum, N. Kawakami, A. Keren, J.-P. Khoo, C. H. King, L. M. Knowlton, O. Kobusingye, A. Koranteng, R. Krishnamurthi, R. Lalloo, L. L. Laslett, T. Lathlean, J. L. Leasher, Y. Y. Lee, J. Leigh, S. S. Lim, E. Limb, J. K. Lin, M. Lipnick, S. E. Lipshultz, W. Liu, M. Loane, S. L. Ohno, R. Lyons, J. Ma, J. Mabweijano, M. F. MacIntyre, R. Malekzadeh, L. Mallinger, S. Manivannan, W. Marcenes, L. March, D. J. Margolis, G. B. Marks, R. Marks, A. Matsumori, R. Matzopoulos, B. M. Mayosi, J. H. McAnulty, M. M. McDermott, N. McGill, J. McGrath, M. E. Medina-Mora, M. Meltzer, Z. A. Memish, G. A. Mensah, T. R. Merriman, A.-C. Meyer, V. Miglioli, M. Miller, T. R. Miller, P. B. Mitchell, A. O. Mocumbi, T. E. Moffitt, A. A. Mokdad, L. Monasta, M. Montico, M. Moradi-Lakeh, A. Moran, L. Morawska, R. Mori, M. E. Murdoch, M. K. Mwaniki, K. Naidoo, M. N. Nair, L. Naldi, K. V. Narayan, P. K. Nelson, R. G. Nelson, M. C. Nevitt, C. R. Newton, S. Nolte, P. Norman, R. Norman, M. O'Donnell, S. O'Hanlon, C. Olives, S. B. Omer, K. Ortblad, R. Osborne, D. Ozgediz, A. Page, B. Pahari, J. D. Pandian, A. P. Rivero, S. B. Patten, N. Pearce, R. P. Padilla, F. Perez-Ruiz, N. Perico, K. Pesudovs, D. Phillips, M. R. Phillips, K. Pierce, S. Pion, G. V. Polanczyk, S. Polinder, C. A. Pope, S. Popova, E. Porrini, F. Pourmalek, M. Prince, R. L. Pullan, K. D. Ramaiah, D. Ranganathan, H. Razavi, M. Regan, J. T. Rehm, D. B. Rein, G. Remuzzi, K. Richardson, F. P. Rivara, T. Roberts, C. Robinson, De Leòn, Felipe Rodriguez, L. Ronfani, R. Room, L. C. Rosenfeld, L. Rushton, R. L. Sacco, S. Saha, U. Sampson, L. Sanchez-Riera, E. Sanman, D. C. Schwebel, J. G. Scott, M. Segui-Gomez, S. Shahraz, D. S. Shepard, H. Shin, R. Shivakoti, D. Silberberg, D. Singh, G. M. Singh, J. A. Singh, J. Singleton, D. A. Sleet, K. Sliwa, E. Smith, J. L. Smith, N. J. C. Stapelberg, A. Steer, T. Steiner, W. A. Stolk, L. J. Stovner, C. Sudfeld, S. Syed, G. Tamburlini, M. Tavakkoli, H. R. Taylor, J. A. Taylor, W. J. Taylor, B. Thomas, W. M. Thomson, G. D. Thurston, I. M. Tleyjeh, M. Tonelli, J. A. Towbin, T. Truelsen, M. K. Tsilimbaris, C. Ubeda, E. A. Undurraga, van der Werf, Marieke J, J. van Os, M. S. Vavilala, N. Venketasubramanian, M. Wang, W. Wang, K. Watt, D. J. Weatherall, M. A. Weinstock, R. Weintraub, M. G. Weisskopf, M. M. Weissman, R. A. White, H. Whiteford, S. T. Wiersma, J. D. Wilkinson, H. C. Williams, S. R. M. Williams, E. Witt, F. Wolfe, A. D. Woolf, S. Wulf, P.-H. Yeh, A. K. M. Zaidi, Z.-J. Zheng, D. Zonies, A. D. Lopez, and C. J. L. Murray. 2012. Years lived with disability (YLDs) for 1160 sequelae of 289 diseases and injuries 1990–2010: a systematic analysis for the Global Burden of Disease Study 2010. The Lancet **380**:2163–2196.

30. **Wetter, M., M. Kowarik, M. Steffen, P. Carranza, G. Corradin, and M. Wacker.** 2013. Engineering, conjugation, and immunogenicity assessment of Escherichia coli O121 O antigen for its potential use as a typhoid vaccine component. Glycoconj J **30**:511–522.

31. **WHO.** 2005. Guidelines for the control of shigellosis, including epidemics due to Shigella dysentaeriae type 1, Geneva, Switzerland.
32. **Zinkernagel, R. M.** 2012. Immunological memory ≠ protective immunity. Cell. Mol. Life Sci. **69**:1635–1640.

This confidential document is the property of LimmaTech Biologics AG

Appendix A

Laboratory Values Guidance

Hematology Toxicology Table

Test	Quest Normal	Mild (Grade 1)	Moderate (Grade 2)	Severe (Grade 3)	Potentially Life Threatening
Hemoglobin (g/dL)	M: LLN = 13.2<br>F: LLN = 11.7	M: 11.0-12.5<br>F: 9.5-10.7	M: 9.0-10.9<br>F: 8.0-9.4	M: <9.0<br>F: <8.0	Potentially Life Threatening
Neutrophils (cells/mm3)	1,500-7,800	750-999	500-749	<500	Potentially Life threatening
Leukocytes (white blood cells)	3,800-10,800				
Leukopenia		2,500-3,300	1,500-2,499	1,000-1,499	< 1,000
Leukocytosis		11,500-13,000	13,001-15,000	>15,000	Potentially Life threatening
Lymphocytes (cells/mm3)	850-3,900	750-849	500-749	250-499	< 250
Eosinophils (cells/mm3)	15-500	551-1,500	1,501-5,000	> 5,000	Hypereosinophilic
Platelets decreased –<br>103/mm3	140,000-400,000	100,000-125,000	75,000-99,000	25,000-74,999	< 25,000

Chemistry Toxicology Table

**Test**	**Quest Normal**	**Mild (Grade 1)**	**Moderate (Grade 2)**	**Severe (Grade 3)**	**Potentially Life Threatening (Grade 4)**
Sodium	135-146 (mmol/L)				
Hyponatremia		132-134	130-131	125-129	< 125
Hypernatremia		147-148	149-150	151-152	> 152
Potassium	3.5-5.3 (mmol/L)				
Hypokalemia		3.3-3.4	3.1-3.2	2.9-3.0	< 2.9
Hyperkalemia		5.6-5.7	5.8-5.9	6.0-6.1	> 6.1
Chloride	98-110 mmol/L	90-97	80-89	70-79	<70
Bicarbonate	19-30 mmol/L	16-18	13-15	10-12	<10

Glucose, Random	65-139 (mg/dL)				
Hyperglycemia		140-155	156-200	> 200	Insulin requirements
Hypoglycemia		60-64	55-59	45-54	< 45
SGOT/AST (elevation)	M: 10-40 U/L F: 10-30 U/L	M: 50-100<br>F: 38-75	M: 101-200<br>F: 76-150	M: 201-400<br>F: 151-300	M: > 400<br>F: > 300
SGPT/ALT (elevation)	M: 9-46 U/L F: 6-29 U/L	M: 61-150<br>F: 41-100	M: 151-300<br>F: 101-200	M: 301-600<br>F: 201-400	M: > 600<br>F: > 400
BUN (elevation)	7-25	26-28	29-31	> 31	Requires dialysis
Creatinine (elevation)	M: 0.6-1.35<br>F: 0.5-1.1	1.1 to < 1.3 x ULN	1.3 to <1.8 x ULN	1.8 to <3.4 x ULN	≥3.4 x ULN

References Ranges and Adverse Coding for Vital Signs Parameters

Vital Signs	Mild (Grade 1)	Moderate (Grade 2)	Severe (Grade 3)	Potentially Life Threatening (Grade 4)
**Heart rate**				
Tachycardia	101–115	116-130	>130	ER visit or hospitalization for arrhythmia
Bradycardia	50-54[a]	45–49	<45	ER visit or hospitalization for arrhythmia
**Blood Pressure**				
Hypertension (systolic, mm Hg)	140-160	161 - 180	>180	Potentially Life threatening, ER visit/hospitalization for malignant hypertension
Hypertension (diastolic, mm Hg)	90-100	101 – 110	>110	Potentially Life threatening, ER visit/hospitalization for malignant hypertension
Hypotension (systolic, mm Hg)[b]	85–89	80 – 84	<80	ER visit/hospitalization for hypotensive shock

[a] Grade 1 bradycardia will not be considered an abnormality for this study unless judged to be clinically significant by the PI or the PI in consultation with the Research Monitor and sponsor.

[b] If a subject has a baseline systolic BP in the 90's then a decrease in BP < 10 without associated clinical symptoms will not be considered an abnormality for this study unless judges to be clinically significant by the PI.
